# Oral hygiene practices and dental caries experience in Nigeria: A systematic review and meta-analysis

**DOI:** 10.1371/journal.pone.0342631

**Published:** 2026-08-11

**Authors:** Adetayo Aborisade, Amina Mohammed Ali, Chizoba Okolo, Titilola Abike Gbaja-Biamila, Folahanmi Tomiwa Akinsolu, Abideen Olurotimi Salako, Ifeoluwa Eunice Adewole, Mobolaji Timothy Olagunju, Olunike Rebecca Abodurin, George Uchenna Eleje, Ucheoma Catherine Nwaozuru, Adebola Oluyemisi Ehizele, Joanne Marie Lusher, Maha El Tantawi, Oliver Chukwujekwu Ezechi, Morẹ́nikẹ Oluwátóyìn Foláyan

**Affiliations:** 1 Department of Oral Diagnostic Sciences, Bayero University, Kano, Nigeria; 2 Department of Oral Maxillofacial Surgery, Bayero University, Kano, Nigeria; 3 Department of Child Dental Health, Bayero University, Kano, Nigeria; 4 Oral Health Initiative, Center for Reproductive and Population Health Studies, Nigerian Institute of Medical Research, Yaba, Lagos, Nigeria; 5 Department of Clinical Sciences, Nigerian Institute of Medical Research, Lagos, China; 6 Department of Public Health, Faculty of Basic Medical and Health Sciences, Lead City University, Ibadan, Nigeria; 7 Department of Epidemiology and Health Statistics, Nanjing Medical University, China; 8 Lagos State Health Management Agency, Lagos, China; 9 Department of Obstetrics and Gynaecology, Nnamdi Azikiwe University Teaching Hospital, Nnewi, Nigeria; 10 Effective Care Research Unit, Department of Obstetrics and Gynaecology, Nnamdi Azikiwe University, Awka, Nigeria; 11 Department of Community Medicine and Primary Healthcare, Nnamdi Azikiwe University Teaching Hospital, Nnewi, Nigeria; 12 Wake Forest School of Medicine, North Carolina, United States of America; 13 Department of Periodontics, School of Dentistry, College of Medical Sciences, University of Benin, Benin City, Nigeria; 14 Provost’s Group, Regent’s University London, United Kingdom; 15 Department of Pediatric Dentistry and Dental Public Health. Faculty of Dentistry, Alexandria University, Egypt; 16 Department of Child Dental Health, Obafemi Awolowo University, Ile-Ife, Nigeria; Innovative Aid, CANADA

## Abstract

**Background:**

Oral hygiene is linked with dental caries experience. This systematic review and meta-analysis assessed the associations between oral hygiene status, the frequency of tooth brushing, and the prevalence of dental caries in Nigeria. It also examined how the geographical zones and dentition type moderate these associations, and identified the tools commonly used for oral hygiene maintenance.

**Methods:**

The systematic review and meta-analysis was registered with PROSPERO (CRD42022367763). A search was conducted in PubMed, Web of Science, Scopus, African Journals Online, African Index Medicus, and Google Scholar in January 2026 for studies conducted between January 2001 and December 2025. Observational studies and clinical trials reporting baseline caries prevalence were included. There was no language restriction. Studies were excluded if they did not provide information on the sample size, had no study outcome data, featured duplicate samples, or were review articles, meta-analyses, case reports, case series, in vitro studies, commentaries/letters (editorials, opinion pieces) devoid of primary data. Pooled odds ratios (ORs) were estimated using random-effects models. Subgroup analyses were conducted by dentition type, geopolitical zone, study design, publication year, mean age, and sample size.

**Results:**

Twenty-three cross-sectional studies were included, of which 20 (86.9%) were conducted in Southern Nigeria. Pooled data from 10 studies suggested poor oral hygiene was associated with 100% higher odds of dental caries (95% CI: 1.26–3.96; p = 0.003). After removing an influential outlier, the analysis showed a 76% increase in dental caries prevalence associated with poor oral hygiene (95% CI: 1.41–2.18; p = 0.003; I² = 0.00%). Brushing at least twice daily was associated with reduced dental caries (p < 0.001). Dentition type and publication year were significant moderators. The most common oral cleaning tool was a toothbrush with toothpaste, though chewing sticks, cotton wool, and other traditional tools were also reported.

**Conclusion:**

Poor oral hygiene was linked to increased odds for dental caries, while twice-daily tooth brushing was protective in Nigeria. The type of cleaning tool was not significantly associated with dental caries risk, highlighting the importance of brushing frequency over tool type. Studies reporting on oral hygiene and dental caries in Northern Nigeria are needed to improve the national representativeness of the data.

## Introduction

Oral hygiene is essential for oral and general health. It encompasses a range of preventative practices that individuals adopt to maintain the cleanliness and overall health of the oral cavity [1–3]. Oral hygiene tools include the use of dental floss, interdental cleaning, antimicrobial rinses, and tongue cleaning to control plaque accumulation [4,5]. Furthermore, cleaning of the dorsum of the tongue using a tongue scraper or a toothbrush helps to eliminate debris that can be an oral bacterial reservoir [6–8]. Key oral hygiene method, however, is the regular tooth brushing with a toothbrush and toothpaste, which removes dental plaque and food particles, and prevents dental caries. [9]. Besides toothbrushing, the use of dental floss, interdental cleaning, antimicrobial rinses, and tongue cleaning controls plaque accumulation.

The link between oral hygiene and dental caries is well-established, as dental plaque initiates and advances dental caries lesions [10,11]; therefore, efforts to control plaque would positively affect the prevention of dental caries [12]. Regular toothbrushing with fluoridated toothpaste, and dental floss helps remove plaque and reduce the availability of fermentable carbohydrates for bacterial acid production [5,13–15]. Oral hygiene practices can reduce the risk of developing dental caries when combined with professional preventive treatments such as scaling and polishing [11,14,16,17]. Proper oral hygiene removes plaque from tooth surfaces [10,18], which prevents the initiation of enamel demineralization caused by acid produced by bacterial fermentation of carbohydrates in the oral cavity [3,19].

Globally, untreated dental caries in permanent teeth remains the most prevalent health condition, affecting an estimated two billion people in 2022 [20]. Despite this burden, there is limited context-specific evidence from Nigeria. The most recent national survey on dental caries in Nigeria was conducted in 1995 [21], and a national survey on oral hygiene practices was last conducted in 2014 [22]. Since then, major socio-economic [23] and demographic [24] shifts, rapid urbanization [25,26], dietary changes (including increased sugar consumption) [27], and disparities in access to preventive oral health services [27] have likely altered the relationship between oral hygiene practices and dental caries. Furthermore, regional diversity in cultural practices, such as the use of chewing sticks alongside toothbrushes, necessitates evidence that reflects both modern and traditional practices [28]. Without updated, nationally representative evidence, policymakers and oral health professionals lack the data needed to design effective, culturally relevant, and equitable interventions for dental caries prevention. The evidence also needs to support planning for northern and southern Nigeria, where differences in health expenses and disease burden [29], oral hygiene behaviors [21], service availability, and socio-demographic conditions [30] demand region‑specific strategies.

In the absence of national surveillance, a systematic review can address the critical evidence gap and offer insights to guide oral health policies, prevention strategies, and public health programs in line with the United Nations Sustainable Development Goal 3 on good health and well-being [31]. This systematic review and meta-analysis therefore aimed to synthesize available evidence on the association between oral hygiene practices and dental caries in Nigeria and to provide a national estimate from published observational studies.

## Methods

### Study protocol

This systematic review and meta-analysis were initially registered with PROSPERO (CRD42022367763) in 2022 and updated on the 24^th^ of January 2026. The study was reported in accordance with the Preferred Reporting Items for Systematic Reviews and Meta-analyses (PRISMA) statement and checklist [32,33] (S1 File). Two blinded reviewers performed each review stage, and disagreements were resolved through discussions with a third reviewer.

### Research questions

The following research questions guided the study: 1) Is there an association between oral hygiene status and dental caries? 2) Is there an association between the frequency of tooth brushing and the prevalence of dental caries? 3) What tools were used by participants for maintaining oral hygiene?

### Search strategy

Five databases (PubMed, Web of Science, Scopus, African Journals Online, and African Index Medicus) and Google Scholar were searched for relevant articles published with no language restriction. The initial search syntax was developed for PubMed and later adapted to fulfill the unique search criteria of the other databases (S2 File).

To ensure a comprehensive search, grey literature was sought from the libraries of the National Postgraduate Medical College of Nigeria and the West African College of Surgeons. The libraries of the National Postgraduate Medical College of Nigeria and the West African College of Surgeons provide the biggest local library of theses and postgraduate dissertations. In addition, the reference lists of all retrieved articles, systematic reviews, and meta-analyses were examined. To identify further sources, study investigators were contacted directly for inaccessible publications and for clarification on missing data, theses, and unpublished manuscripts.

### Inclusion and exclusion criteria

All published and unpublished studies, including hand searches conducted in Nigeria between January 2001 and December 2025, that reported on associations between oral hygiene and the prevalence of dental caries were eligible for study inclusion. Study designs eligible for inclusion were cross-sectional, cohort, and case-control studies. Clinical trials that provided relevant baseline prevalence data were also included. Studies were also included if they presented available data for at least one of the primary outcomes indicated in Table 1, whose reported odds ratio (OR) and 95% confidence interval (CI) were analyzed by univariate or multivariate analyses, and studies whose data could be analyzed to generate variables for the current study.

**Table 1:** Eligibility criteria using PECOS (Population, Exposure, Comparisons, Outcomes, Studies) framework.

Population	Individuals residing in Nigeria
Exposure	Oral hygiene practices (tooth brushing frequency and tooth cleaning methods – toothbrush, chewing stick, or other devices), and oral hygiene status.
Comparators	Variations in oral hygiene practices (e.g., brushing ≥2 times/day vs. once/day; good vs. poor hygiene; toothbrush vs. chewing stick).
Outcome	Presence of dental caries.
Studies	Studies reporting prevalence data.

Studies were excluded if they did not provide information on the sample size, had unavailable outcome data, or featured duplicate samples. Review articles, systematic reviews and meta-analyses, case reports or case series, in vitro studies, commentaries/letters (editorials, opinion pieces), devoid of primary data, were excluded. Studies with overlapping data from other included studies were also excluded. Excluded studies were reported in S3 Table.

### Selection of studies

Studies were screened using the PECOS framework (Population, Exposure Comparators, Outcomes, Time, Studies). Table 1 presents the PECOS framework used for this study [34]. In this review, dental caries was the outcome of interest. Oral hygiene practices were considered the exposures, and different categories of practices served as comparators. This distinction was made explicit to ensure clarity in the analytic framework and to avoid misinterpretation of dental caries as an exposure or risk factor.

Three authors (AA, AMA, and CO) independently reviewed the titles and abstracts of each study that met the inclusion criteria after removing duplicates and downloading them to the reference management software EndNote 7.8. Studies that did not meet the inclusion criteria and those where the full text was unavailable were excluded. Two reviewers (AA and AMA) independently assessed the eligibility of the retrieved manuscripts, and any disagreements were resolved by discussion or recourse to a third reviewer (MOF).

### Data extraction

Four independent reviewers (AA, TAG, IEA, and ORA) used a pretested data extraction form prepared in Microsoft Excel to independently extract information related to the author’s name and year of publication. In addition, specific details about the study design, location, and study setting were captured. Details about the study participants – sample size, age distribution, sex of participants, and other unique characteristics – were extracted. Information about participants’ oral hygiene practices was also extracted. These details included measures of oral hygiene status, frequency and duration of hygiene practices, and the tools or methods employed for oral hygiene. Simultaneously, dental caries assessment data were cataloged, highlighting each study’s tools or methods for assessing dental caries. The percentage of participants with dental caries was also extracted. Lastly, the results or association measures, whether in the form of prevalence or statistical ratios indicating the associations between oral hygiene practices and dental caries, were recorded. Any discrepancies were resolved by a fifth reviewer (MOF).

### Quality and risk of bias assessment

Four independent reviewers (AA, TAG, IEA, and ORA) assessed the methodological quality and risk of bias in the included studies, with discrepancies resolved by a fifth reviewer (FTA) using an adapted version of the risk of bias tool for prevalence studies according to the modified Joana Briggs Institute Assessment for Risk of Bias [35]. This risk of bias tool, designed for prevalence studies, was applied as the most appropriate quality assessment approach because the Odds Ratios (ORs) synthesized in the meta-analysis were calculated directly from raw prevalence counts (number of events and non-events) reported in the included cross-sectional studies. The validity of these ORs is fundamentally dependent on the methodological quality of the prevalence data*.* The total score ranged from 0 to 9, with the overall score categorized as follows: 0–3: “high risk,” 4–6: “moderate risk,” and 7–9: “low risk” of bias.

### Certainty of evidence

The certainty of evidence for each outcome using the Grading of Recommendations Assessment, Development, and Evaluation (GRADE) approach was not conducted because, under this framework, cross-sectional studies are rated as low certainty evidence for questions of association because of the inherent risk of bias from temporality and unmeasured confounding [36,37]. As no randomized or longitudinal studies were available for any of the comparisons, applying GRADE would uniformly label all pooled estimates as very low certainty, which does not meaningfully discriminate between outcomes. Instead, we presented the risk of bias assessment and the sensitivity analyses as the primary indicators of result robustness.

### Sensitivity analysis

A sensitivity analysis was performed with leave‑one‑out meta‑analysis for the primary outcome of oral hygiene status and caries prevalence, and influence diagnostics were performed using Cook’s distance and DFBETAS to identify outliers [38,39]. We also removed studies with a high risk of bias (as assessed by the modified JBI tool) to test the stability of the pooled estimates [40].

### Assessment for publication bias

Symmetry of the funnel plot was planned to assess the publication bias of the articles included in the systematic review. Egger’s regression test was also utilized to assess publication bias objectively with significance at p < 0.05.

### Statistical analysis

All analyses were conducted using R and R Studio Version 4.5.1 (“Great Square Root”). A descriptive analysis was first undertaken to show the distribution of studies by year of publication, study design, geopolitical region, number of male and female participants, and type of dentition studied: primary (0–5 years), mixed (6–11 years), and permanent (≥12 years). We also described the number of studies reporting an association between oral hygiene practices and dental caries prevalence by geographic region and dentition type.

This meta-analysis applied strict inclusion criteria, selecting only studies that reported definitive, binary data on the presence or absence of dental caries. This ensured an objective and direct assessment of the relationship between oral hygiene practices and caries, aligning with standard epidemiological methods for evaluating disease prevalence and risk factors. Pooled estimates were generated using the Mantel-Haenszel method. Heterogeneity was evaluated using Cochran’s Q test, the I² statistic, and inspection of forest plots. The independent variables were oral hygiene practices (tooth cleaning method, brushing frequency, and oral hygiene status), while the dependent variable was dental caries prevalence. Odds ratios (ORs) with 95% confidence intervals (CIs) were used as the summary measure, categorizing outcomes into dental caries present versus absent. These ORs were calculated from extracted frequencies and denominators reported in the studies, rather than adopting published effect sizes. Because the synthesis was based on prevalence data, a risk of bias tool designed for prevalence studies was applied as the most appropriate quality assessment approach. A p-value <0.05 for the Q test was considered statistically significant, and I² values above 50% indicated substantial heterogeneity. Negative I² values were treated as zero [41,42], following Cochrane Handbook guidance [43,44]. Studies with no events in either arm were excluded from the meta-analysis [40–44].

Associations between oral hygiene practices and dental caries prevalence were evaluated using moderator analysis, including subgroup analysis and univariate meta-regression. When study heterogeneity is high, subgroup analyses were conducted based on the type of dentition (primary, mixed, permanent), geopolitical zone (South and Northern), and study design (population-based, school-based, or other). Univariate meta-regression examined the year of publication, study sample size, and participants’ mean age in each study. Sensitivity analyses involved excluding studies with a high risk of bias and sequentially removing individual studies to assess their influence on pooled estimates. Publication bias was assessed using funnel plots and Egger’s test when at least ten studies reported the same outcome [45,46].

### Ethical approval

Ethical approval was not required for this systematic review as the research was based on information retrieved from published studies.

## Results

### Selection of studies

**Fig 1:**
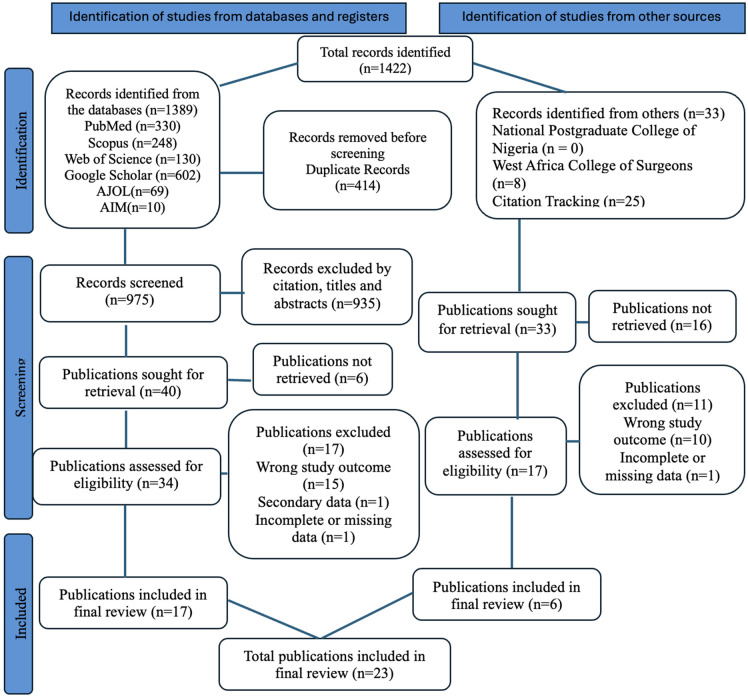
PRISMA flow diagram for the search results.

As shown in Fig 1, 1422 records were retrieved. After removing 414 duplicates, 1008 records remained for eligibility screening. Of these, 935 studies were excluded based on their titles and abstracts. After reviewing the full-text records, 23 met the inclusion criteria [4,7,15,18,47–65].

### Characteristics of included studies

The characteristics of the 23 included studies are detailed in Table 2. The participant population size in each study ranged from 124 to 2,107 individuals, culminating in a combined total of 15,797 participants. The aggregated sample comprised 7838 males and 7959 females with participants’ ages ranging from six months to 64 years.

**Table 2:** Characteristics of included studies.

S/No	Study Reference	Title	Study Design	City/State	Region	Sample Size	Age Range	Gender Distribution (M/F)
1	Sowole et al., 2007 [15]	Dental Caries Pattern and Predisposing Oral Hygiene Related Factors in Nigerian Preschool Children	Cross-sectional	Lagos	Southwest	389	6-71 months	217 / 172
2	Okoye et al., 2011 [47]	Prevalence of Dental Caries in a Nigerian Rural Community: A Preliminary Local Survey	Cross-sectional	Enugu	Southeast	301	11-16 years	100 / 201
3	Ajayi and Abidemi-Solanke, 2014 [48]	Socio-behavioural risk factors of dental caries among selected adolescents in Ibadan, Nigeria	Cross-sectional	Ibadan	Southwest	914	10-19 years	451 / 463
4	Folayan et al., 2015 [49]	Prevalence And Early Childhood Caries Risk Indicators in Preschool Children in Suburban Nigeria	Cross-sectional	Osun	Southwest	497	6-71 months	266 / 231
5	Olatosi et al., 2015 [50]	The Prevalence of Early Childhood Caries and Its Associated Risk Factors Among Preschool Children Referred to a Tertiary Institution	Cross-sectional	Lagos	Southwest	202	6-71 months	44 / 158
6	Olabisi et al., 2015 [7]	Prevalence Of Dental Caries and Oral Hygiene Status of a Screened Population in Port Harcourt, Rivers State, Nigeria	Cross-sectional	Rivers	South-South	288	15-64 years	133 / 155
7	Onyejaka and Amobi, 2016 [51]	Risk Factors of Early Childhood Caries among Children in Enugu, Nigeria	Cross-sectional	Enugu	Southeast	429	9 months – 5 years	226 / 203
8	Akinyamoju et al., 2018 [18]	Dental Caries and Oral Hygiene Status: Survey of Schoolchildren in Rural Communities, Southwest Nigeria	Cross-sectional	Ogun	Southwest	778	7-17 years	424 / 354
9	Oyedele et al., 2018 [52]	Impact of Oral Hygiene and Socio-demographic Factors on Dental Caries in a Suburban Population in Nigeria	Cross-sectional	Osun	Southwest	2,107	8-16 years	982 / 1125
10	Kolawole et al., 2019 [4]	Association Between Malocclusion, Caries, And Oral Hygiene in Children 6–12 Years Old Residents in Suburban Nigeria	Cross-sectional	Osun	Southwest	495	6-12 years	242 / 253
11	Abiola et al., 2019 [53]	Dental Caries Occurrence and Associated Oral Hygiene Practices Among Rural and Urban Nigerian Preschool Children	Cross-sectional	Lagos	Southwest	404	18-60 months	208 / 196
12	Nwathor et al., 2019 [54]	Oral Hygiene Status, Practices, and Awareness of Medium Security Prison Inmates in Northeastern Nigeria	Cross-sectional	Bauchi	Northeast	280	<30->50 years	276 / 4
13	Disa et al., 2019 [55]	Predictors of dental caries among adults and adolescents in a dental clinic in North-eastern Nigeria	Case-control	Yobe	Northeast	124	15-63 years	59 / 65
14	Oyapero et al., 2020 [56]	Association Between Dental Caries, Odontogenic Infections, Oral Hygiene Status, and Anthropometric Measurements of Children in Lagos, Nigeria	Cross-sectional	Lagos	Southwest	278	1-15 years	132 / 146
15	Arowolo, 2020 [57]	Determination Of the Association Between Nutritional Status and Dental Caries In 6–16-Year-Old School Children in Ile – Ife	Cross-sectional	Osun	Southwest	1,502	6-16 years	689 / 813
16	Olatosi et al., 2020 [58]	Disparities in Caries Experience and Socio-Behavioural Risk Indicators Among Private School Children in Lagos, Nigeria	Cross-sectional	Lagos	Southwest	592	5-16 years	307 / 285
17	Folayan et al., 2021 [59]	Associations Between a History of Sexual Abuse and Dental Anxiety, Caries Experience, and Oral Hygiene Status Among Adolescents in Sub-Urban South West Nigeria	Cross-sectional	Osun	Southwest	1,056	10-19 years	598 / 458
18	Onyejaka et al., 2021 [60]	Prevalence and Associated Factors of Dental Caries Among Primary School Children in Southeast Nigeria	Cross-sectional	Enugu	Southeast	657	5-17 years	316 / 341
19	Idowu et al., 2021 [61]	Dental Caries Prevalence, Restorative Needs and Oral Hygiene Status in Adult Population: A Cross-sectional Study among Nurses in Jos University Teaching Hospital, Jos, Nigeria	Cross-sectional	Jos	North-central	251	9–64 years	77 / 174
20	Oyedele et al., 2021 [62]	Comparison of Dental Caries and Oral Hygiene Status of Children in Suburban areas with those in the Rural Population of Southwestern Nigeria	Cross-sectional	Osun/Ogun	Southwest	1,397	8-12 years	706 / 691
21	Folayan et al., 2021 [63]	Individual and familial factors associated with caries and gingivitis among adolescent’s resident in a semi-urban community in South Western Nigeria	Cross-sectional	Osun	Southwest	1,472	10-19 years	846 / 626
22	Oyedele et al., 2022 [64]	Associations Between Dental Caries, Oral Hygiene Status, And Oral Health Practices of First-Year Undergraduates in A Private University in Nigeria	Cross-sectional	Ogun	Southwest	1,164	15-23 years	443 / 721
23	Afolabi, 2023 [65]	Comparative assessment of the oral health status of children aged 4–16 years with and without sickle cell anaemia resident in Ile-Ife and Ilesha, Osun State, Nigeria	Case-control	Osun	Southwest	220	6-16 years	96 / 124

The publication timeline of the included studies spans 16 years, from 2007 to 2023, and can be categorized into distinct periods. (1) Early Period (2007–2015): Six studies (26.1%) were published during this foundational phase [7,15,47–50]. (2) Middle Period (2016–2020): Ten studies (43.5%) were published, indicating an increase in research activity [4,18,51–58]. (3) Recent Period (2021–2023): Seven studies (30.4%) were published in the most recent years, demonstrating sustained interest in the topic [59–65]. All 23 studies employed an observational design. Twenty-one studies (91.3%) were cross-sectional surveys [4,7,15,18,47–54,56–64], while two studies (8.7%) utilized a case-control design [55,65].

Geographically, the studies showed a significant concentration in Southern Nigeria. Twenty studies (86.9%) were conducted in the southern region, compared to three studies (13.1%) from Northern Nigeria. Within Southern Nigeria, the Southwest zone was the most researched, with 16 studies (69.6% of the total). These were conducted across Lagos [15,50,53,56,58], Osun [4,49,52,57,59,62,63,65], Ogun [18,64], 62], and Oyo (Ibadan) [48] states. Three studies (13.0%) were conducted in the Southeast (Enugu) [47,51,60], and one study (4.3%) was from the South-South (Rivers) region [7]. In Northern Nigeria, two studies were from the Northeast (Bauchi and Yobe) [54,55], and one study was from the North-central (Jos) zone [61].

### Oral hygiene status, frequency, and devices used for oral hygiene

#### Oral hygiene status assessment.

Oral hygiene status was assessed in 14 (60.9%) of the 23 studies for association with the prevalence of dental caries. The Simplified Oral Hygiene Index (OHI-S) was the predominant tool, used in 12 (85.7%) of the studies [4,15,48,49,51–53,60–62,64,65], and one study used the Plaque Index [63]. Most studies that used the OHI-S reported the distribution of participants across categories of good, fair, and poor oral hygiene. One study did not specify its tool for OHI measurement [52], while another presented mean OHI-S scores stratified by urban/rural setting, gender, and age [53].

#### Measures of oral hygiene practices.

Studies assessed various dimensions of oral hygiene behaviour:

1. **Frequency of Mouth Cleaning:** Reported in 17 studies [15,18,47–50,52–54,56–58,60,62–65]. The reported frequencies ranged from ‘none’ [15,51], rarely [54], and ‘occasionally [18,47] to once daily, twice daily, and more than twice daily [15,18,47–50,52–54,56–58,60,62–65].
2. **Devices Used for Mouth Cleaning:** Documented in 14 studies [15,18,47,48,50,53–55,57,59,61,63–65]. A wide array of devices was reported, including toothbrushes with toothpaste (most common), chewing sticks, cotton wool, dental floss, gauze, fingers, herbs, and traditional agents like glycerine or ground glass.
3. **Other Behavioural Variables:** Several studies explored additional factors, including the individual responsible for cleaning (child or caregiver) [50], supervision of brushing [50,53], age at onset of oral cleaning [50], timing of cleaning (e.g., before/after meals) [54,61,64], and the frequency of changing cleaning devices [18,53,55].

### Dental caries assessment

All 23 studies used the DMFT/dmft indices to assess caries prevalence and severity. Two studies additionally employed the PUFA/pufa index to measure the clinical consequences of untreated caries [4,55]. The assessed dentitions were: primary dentition (7 studies) [15,49–51,53,57,65], mixed dentition (8 studies) [4,18,48,52,56,58,60,62], and permanent dentition (9 studies) [7,47,54,55,59,61,63–65]. The reported caries prevalence ranged widely from 2.2% to 79.1%.

### Association between oral hygiene status and dental caries

Of the 19 studies that assessed oral hygiene status, 14 (73.7%) investigated its association with caries [4,15,48,49,51–53,56,60–65]. A consistent finding was that poor oral hygiene status was significantly associated with a higher prevalence or severity of caries in eight of these studies [4,15,51,52,56,57,64,65]. For example, poor oral hygiene increased the odds of caries by factors ranging from 1.8 to 15.5 [4,57,64]. Conversely, good [60] and fair [51,52,60] oral hygiene were associated with a lower risk for caries in several studies. Fair oral hygiene was also associated with increased odds of caries [52,64]. Some studies reported a dose-response relationship, where the risk of caries increased with higher (worse) OHI-S scores [4,53]. Four studies found no significant association [48,51,63,65].

### Association between oral hygiene practices and dental caries

The evidence for associations between specific oral hygiene behaviours and caries was less consistent Table 3:

- **Frequency of Cleaning:** Most studies (8/10) found no significant association between brushing frequency (e.g., once vs. twice daily) and caries prevalence [18,47,48,54,58,59,63,65]. Two studies reported protective effects for brushing at least twice daily [55,57].
- **Cleaning Devices:** The use of a toothbrush with fluoride toothpaste was generally associated with lower caries experience compared to traditional methods like using glycerine, which was linked to significantly higher odds of caries (OR: 17.7; 95% CI: 3.0–103.7) [50]. One study found that chewing stick users had more caries than toothbrush users among males [47]. However, most studies found no significant association for the use of toothbrushes, chewing sticks, floss, or other specific devices when analyzed independently [18,48,54,63].
- **Other behavioural Variables:** Factors such as the brushing supervision, age of onset of cleaning, timing of cleaning [54], and frequency of changing devices [18] were not associated with caries prevalence. One study found that not cleaning after every meal was protective [55].

### Risk of bias assessment of included studies

All but one of the included studies [55] were determined to be of low risk of bias, scoring 7–9 on the quality scale [4,7,15,18,47–54,56–65] (See S4 Table).

**Table 3:** Summary of oral hygiene practices and associations with dental caries in included studies.

Study Author, Year	Oral Hygiene Status (Tool)	Frequency of Mouth Cleaning	Types of Oral Hygiene Devices Used	Caries Prevalence (Assessment Tool)	Key Associations Reported
Sowole et al., 2007 [15]	OHI-S (Good: 78.6%, Fair: 10.5%, Poor: 10.9%)	None: 5%; Once: 71%; Twice: 22.7%; Thrice: 1.3%	Toothbrush (51.4%), Cotton wool (31.4%), Foam (9.6%), Nothing (4.7%), Hand towel (2.9%)	10.6% (dmft)	Higher OHI-S score increased the odds of caries (OR: 2.58, 95% CI:1.03–6.46).
Okoye et al., 2011 [47]	Not assessed	Occasionally: 7%; Once: 73.1%; Twice: 19.9%	Toothbrush/paste (55.8%), Chewing stick (44.2%)	35.5% (DMFT)	Chewing stick users (males) had more caries (P = 0.004). Fluoridated toothpaste users had less caries (P < 0.0001). The frequency of cleaning and types of mouth cleaning devices are not associated with caries prevalence.
Ajayi & Abidemi, 2014 [48]	OHI-S (Good: 66%, Fair: 21.6%, Poor: 12.4%)	Once: 50.2%; Twice: 44.7%; Other: 5.1%	Toothbrush/paste (92.3%), Chewing stick/paste (5%)	10.6% (dmft/DMFT)	No association for frequency, device, or oral hygiene status.
Folayan et al., 2015 [49]	OHI-S (Good: 69.6%, Fair: 28.2%, Poor: 2.2%)	<Twice: 81.1%;> Twice: 8.9%	Not specified	6.6% (dmft)	Fair oral hygiene had a lower caries risk (AOR: 0.05; 95% CI: 0.005–0.10)
Olatosi et al., 2015 [50]	Not disclosed	Once: 90.4%; Twice: 9.6%	Toothbrush/paste (51.3%), Salt water/cloth (18.9%), Ground glass (16.2%), Glycerin (13.6%)	21.2% (dmft/dmfs)	Glycerine use increased caries odds vs. fluoridated toothpaste (OR: 17.7; 95% CI: 3.0–103.7).
Olabisi et al., 2015 [7]	OHI-S (Fair: 68.4%, Good: 12.5%, Poor: 19.1%)	Not specified	Not specified	35.1% (DMFT)	Not reported.
Onyejaka and Amobi, 2016 [51]	OHI-S (Fair: 86.2%, Good: 10.5%, Poor: 3.3%)	None: 0.5%; Once: 81.6%; Twice: 16.6%; > Thrice: 1.4%	Not specified	9.8% (dmft)	Poor oral hygiene (p > 0.001) was associated with dental caries.
Akinyamoju et al., 2018 [18]	OHI-S (Fair: 57.7%, Good: 34.8%, Poor: 7.5%)	Occasionally: 7.1%; Once: 74.1%; > Twice: 18.8%	Toothbrush/paste (65.8%), Both^ (24.8%), Chewing stick (6.2%), Other (3.2%)	2.2% (DMFT/dmft)	No association for frequency or device.
Oyedele et al., 2018 [52]	OHI-S (Poor: 44.7%, Fair: 38.1%, Good: 17.2%)	Not specified	Not specified	12.2% (DMFT/dmft)	Fair (AOR: 1.73; 95% CI: 1.28–2.35) and poor (AOR: 2.13; 95% CI: 1.47–3.07). Poor oral hygiene increased caries odds.
Kolawole et al., 2019 [4]	OHI-S (Fair: 56.2%, Good: 39.4%, Poor: 4.4%)	Not specified	Not specified	14.9% (dmft/DMFT, PUFA/pufa)	Poor oral hygiene increased the odds of dental caries (OR: 1.83; 95% CI: 1.05–3.18).
Abiola et al., 2019 [53]	OHI-S (Mean scores reported) OHI-S urban:1.20 OHI-S rural: 1.40 OHI-S male: 1.36 OHI-S female: 1.21 OHI-S < 3years: 1.26 OHI-S > 3years: 1.38	Once: 68.3%; Twice: 28.5%; Varies: 3.2%	Cotton wool/paste (45.5%), Toothbrush/paste (33.7%), Cotton wool (29.7%), Glycerine (5.4%)	10.9% (dmft)	Each unit increase in OHI-S score increased caries odds by 64%.
Nwhator et al., 2019 [54]	Not assessed	2-3x/day: 38.2%; > 3x/day: 38.6%; Once: 13.2%; Rarely: 9.3%	Water/finger (36.1%), Chewing stick (27.1%), Toothbrush/paste (27.1%)	62.1% (DMFT)	No association for frequency, timing, or device.
Disa et al., 2019 [55]	Not assessed	>Twice: 65.3%; < Twice: 34.7%	Toothbrush/paste (81.5%), Chewing stick (15.3%)	47.6% (DMFT))	Cleaning ≥Twice daily (OR: 0.33; 95% CI: 0.11–0.97) and not after every meal (OR: 0.28; 95% CI: 0.10–0.78) were protective.
Oyapero et al., 2020 [56]	Poor OH: 26.6% (Tool not specified)	Not specified	Not specified	79.1% (dmft/DMFT, PUFA/pufa)	Positive correlation between poor oral hygiene status and caries severity (ρ = 0.307).
Arowolo, 2020 [57]	OHI-S (Fair: 82.7%, Good: 7.7%, Poor: 9.6%)	Twice: 49%; Once: 50.1%; Don’t: 1.1%; > Twice: 0.8%	Toothbrush (97.5%), Chewing stick (1.4%)	6.9% (dmft)	Poor oral hygiene status increased caries odds (AOR: 15.51; 95% CI: 2.80–85.81). Brushing once daily was protective vs. not brushing (AOR: 0.05; 95% CI: 0.02–0.12).
Olatosi et al., 2020 [58]	Not assessed	>Twice: 43.1%; Once: 47.3%; Not daily: 9.6%	Not specified	16.0% (dmft/DMFT)	No association for frequency.
Folayan et al., 2021 [59]	OHI-S (Good: 44.6%, Fair/Poor: 55.4%)	>Twice: 59.7%; < Once: 40.3%	Daily floss use: 11.6%	3.7% (DMFT)	Brushing frequency and floss use are not associated with caries.
Onyejaka et al., 2021 [60]	OHI-S (Fair: 85.2%, Good: 12.6%, Poor: 2.6%)	Not specified	Not specified	22.7% (dmft/DMFT)	Good (OR: 0.18; 95% CI: 0.036–1.003) and air (OR: 0.58; 95% CI: 0.345–0.993) oral hygiene had lower odds of dental caries than poor oral hygiene.
Idowu et al., 2021 [61]	OHI-S (Categories reported)	Once: 68.1%; Twice: 31.9%	Floss use: 10.4%	43.8% (DMFT)	No association between oral hygiene status and caries.
Oyedele et al., 2021 [62]	OHI-S (Fair: 41.4%, Good: 34.9%, Poor: 23.7%)	Not specified	Not specified	17.8% (dmft/DMFT)	Poor oral hygiene status (OR: 1.79; 95% CI: 1.21–2.65) is significantly associated with caries in suburban areas.
Folayan et al., 2021 [63]	Plaque Index	Once: 91.3%; ≥ Twice: 8.7%	Daily floss use: 16.1%	3.4% (DMFT)	No association for brushing frequency, floss use, or oral hygiene status.
Oyedele et al., 2022 [64]	OHI-S (Fair: 58.9%, Good: 30.4%, Poor: 10.7%)	Once: 57.6%; Twice: 41.7%; Thrice: 0.5%	Never floss: 48.6%; Rarely floss: 36.9%	10.6% (DMFT)	Poor (OR: 2.5; 95% CI: 1.3–4.8) and fair (OR: 1.8; 95% CI: 1.1–3.0) oral hygiene associated with caries.
Afolabi, 2023 [65]	OHI-S (Fair: 61.4%, Good: 36.4%, Poor: 2.3%)	Once: 94.7%; Twice: 4.9%; > Twice: 0.4%	Toothbrush/paste: 100%	Primary: 13.5%; Permanent: 5% (dmft/DMFT)	No association for oral hygiene status or frequency in either dentition.

**Note:** “Both” refers to the use of both toothbrush/paste and a chewing stick.**Abbreviations:** OH: Oral Hygiene; OHI-S: Simplified Oral Hygiene Index; dmft: decayed, missing, filled teeth (primary); DMFT: decayed, missing, filled teeth (permanent); PUFA/pufa: pulpal involvement, ulceration, fistula, abscess index; OR: Odds Ratio; AOR: Adjusted Odds Ratio.

### Association between oral hygiene status and dental caries

**Fig 2:**
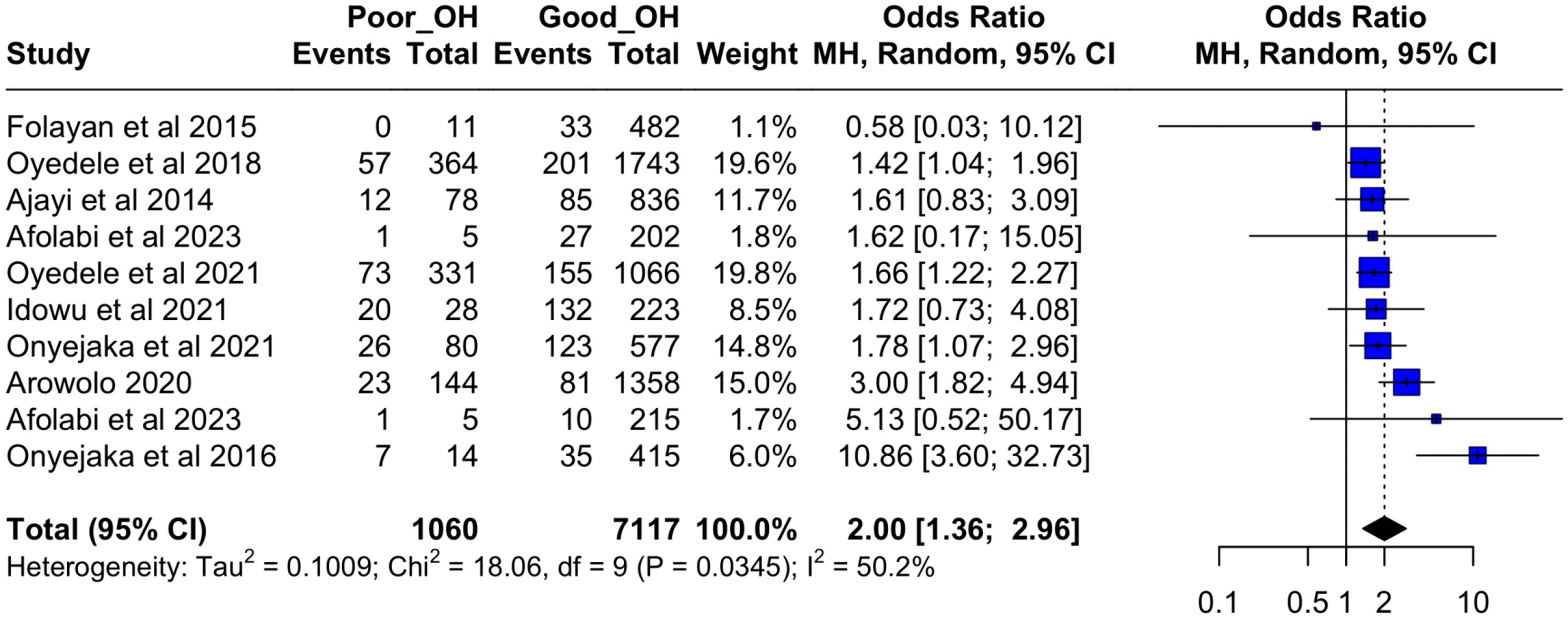
Forest plot showing the meta-analysis of oral hygiene status and caries prevalence.

**Fig 3:**
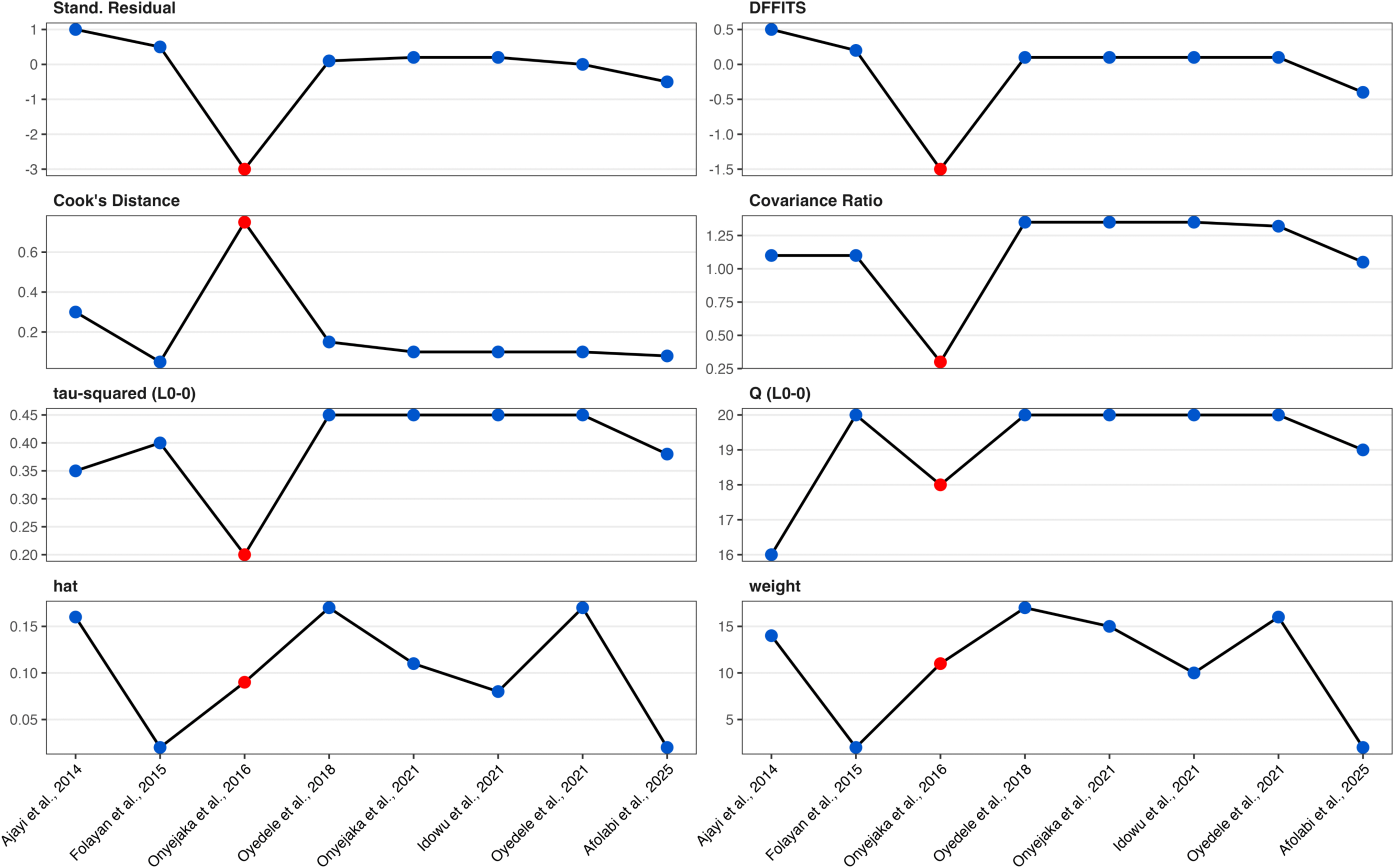
Influential plot showing Onyejaka et al. [60] was an influential study.

Pooled data from 10 studies initially suggested poor oral hygiene was associated with 100% higher caries odds (OR 2.00, 95% CI: 1.26–3.96; p = 0.003) as shown in Fig 2. After removing a study that was an influential outlier [51] (Fig 3), the analysis showed a significant 76% increase in caries prevalence associated with poor oral hygiene (95% CI 1.41–2.18; p = 0.003), with no heterogeneity (I² = 0.00%).

### Sensitivity analysis report

The influential plot (Fig 3) presents the impact of each study on the pooled effect estimate, with Onyejaka et al. [51] identified as an influential outlier. In the plot, this study lies outside the confidence interval of the pooled estimate, indicating that its exclusion would substantially alter the overall effect size. Onyejaka et al. [51] exceeded the thresholds for both the Cook’s distance (0.44) and DFBETAS (0.63). After omitting Onyejaka et al. [51], the direction and significance of the effect remained consistent. The meta‑analysis result is thus moderately robust and the overall conclusion (poor oral hygiene increases caries risk) remains unchanged.

### Subgroup analyses

**Fig 4:**
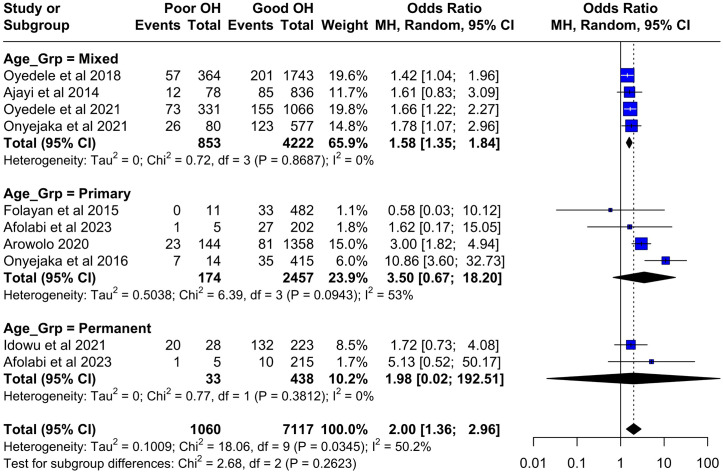
Forest plot showing subgroup analysis by type of dentition for pooled prevalence and oral hygiene status.

Subgroup analysis using the type of dentition showed that the type of dentition was not a significant moderator in the association of caries prevalence with oral hygiene status (chi^2^ = 18.06, p = 0.26). Good oral hygiene status was associated with a 250% (95% CI: 0.67–18.2) reduction in the odds for dental caries in the primary dentition, a 58% (95 CI: 1.35–1.84) reduction in the odds of dental caries in the mixed dentition, and a 98% (95 CI: 0.02–192.5) reduction in the odds of dental caries in the permanent dentition (Fig 4).

Subgroup analysis with study design also shows a non-significant moderating effect of study design (p = 0.31). Due to limited study in Southeastern (n = 2) and Northern (n = 1) regions, subgroup analyses across geopolitical zones could not be conducted.

### Univariate meta-regression

**Fig 5:**
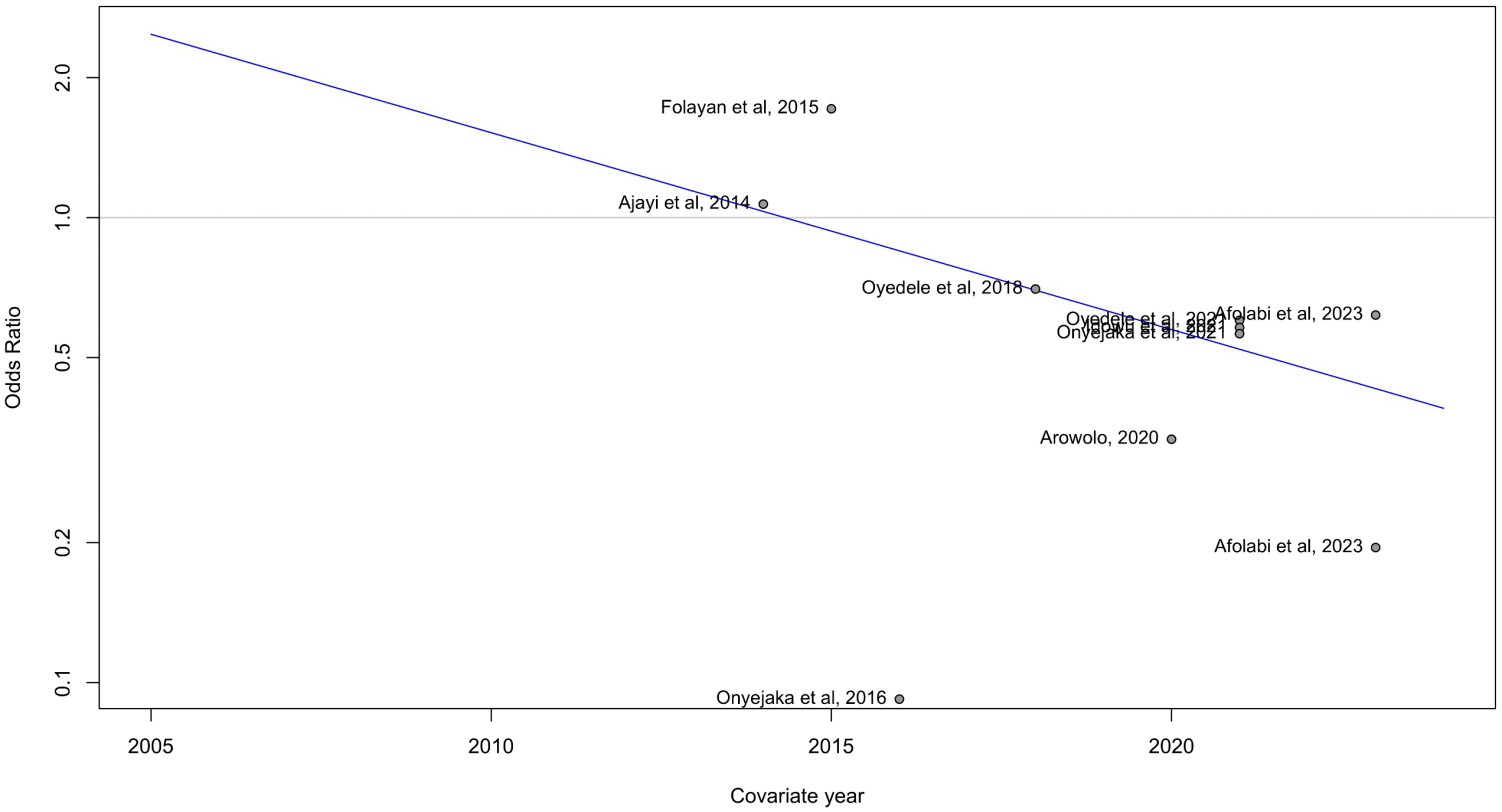
Bubble plot showing the year of publication and caries prevalence.

Univariate meta-regression using the year of publication showed that the year of publication was significant in the association between oral hygiene status and caries prevalence (p = 0.02), Fig 5. R^2^ showed that the year of publication accounts for 75% of all the variance in study heterogeneity.

Meta-regression also showed that the individual study sample size (p = 0.31) and the mean age of participants (p = 0.86) had no significant moderating effect on oral hygiene status and caries prevalence in included studies

### Publication bias

A lack of statistical power from the small number of included studies limits the ability to assess publication bias for oral hygiene status and caries prevalence [45].

### Association between frequency of tooth brushing and dental caries

**Fig 6:**
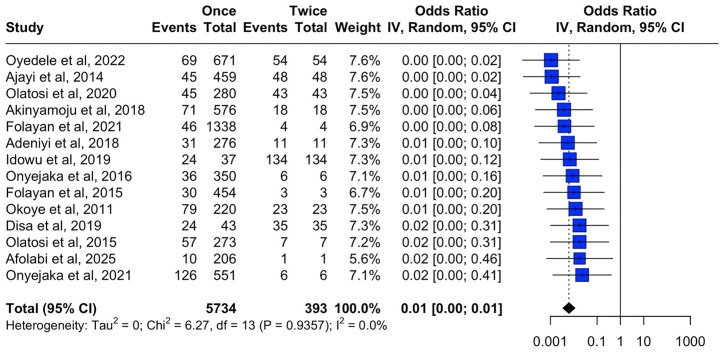
Meta-analysis showing the association of tooth brushing frequency and dental caries.

Analysis of 16 studies showed that brushing teeth at least twice daily was associated with a 99% reduction in the odds of dental caries compared to brushing once daily (OR 0.01, 95% CI < 0.00–0.01; p < 0.001). Results were consistent across studies (I^2^ = 0%) Fig 6.

### Subgroup analysis

Subgroup analysis shows no significant differences in the pooled odds ratio for tooth cleaning frequency and caries prevalence across geopolitical zones (p = 0.11), type of dentition (p = 0.24), and study design (p = 0.83).

### Publication bias

**Fig 7:**
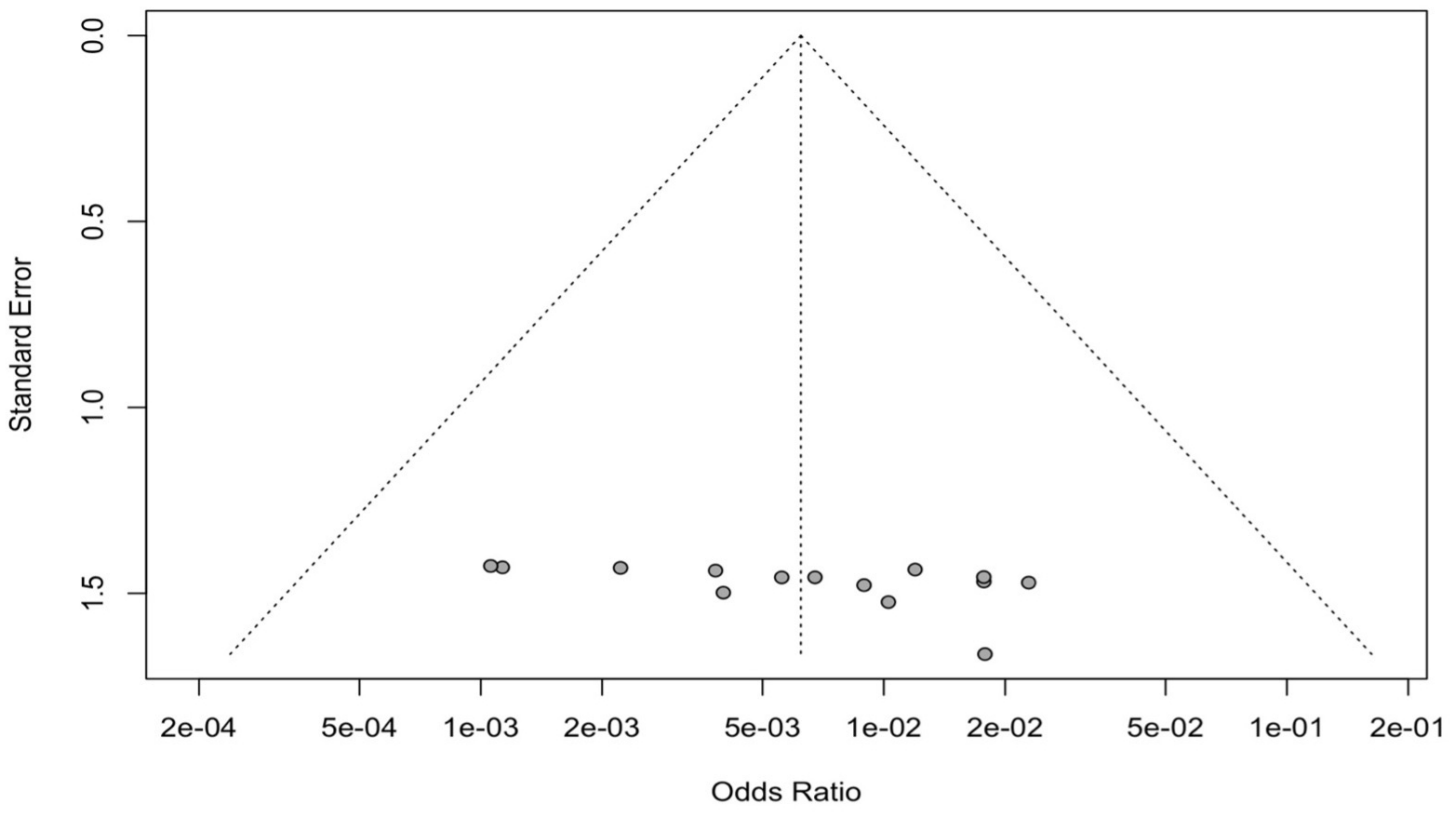
Funnel plot showing plot symmetry and lack of publication bias in the studies showing the association of tooth brushing frequency and dental caries.

Funnel plot, as shown in Fig 7, showed no funnel plot asymmetry to indicate publication bias; objectively, Egger’s test also corroborated the absence of publication bias in studies reporting dental caries prevalence and frequency of tooth brushing.

### Association between tooth cleaning methods and dental caries

**Fig 8:**
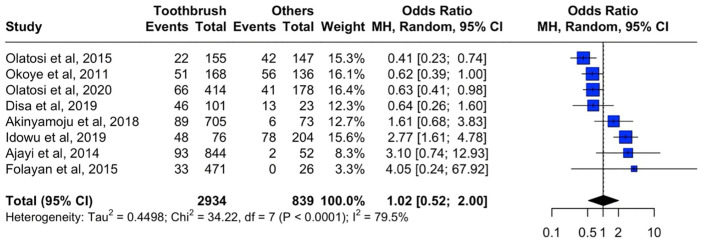
Meta-analysis showing the association of tooth brushing methods and dental caries.

Analysis of eight studies found no significant association between the type of tooth cleaning device and caries prevalence (OR 1.02, 95% CI 0.52–2.00; p = 0.95) as shown in Fig 8. Heterogeneity was high (I² = 79.5%). Sensitivity analysis indicated one influential study; its removal yielded a non-significant 19% reduction in caries odds (OR 0.81, 95% CI 0.42–1.54).

### Subgroup analyses

Toothbrushing was significantly associated with reduced caries prevalence in primary (42% reduction) and permanent (36% reduction) dentition, but with a 92% higher caries prevalence in mixed dentition (p = 0.0015). The geopolitical zones (p = 0.58) and study design (p = 0.13) exerted no significant moderating effect on the association between pooled prevalence of dental caries and tooth cleaning methods Table 4.

**Table 4:** Subgroup analysis for dentition for Toothbrushing and Dental Caries: Results for subgroups (random effects model).

Dentition	OR	95%-CI	tau^2	tau	Q	I^2
**Primary**	0.58	[0.34; 0.99]	0.02	0.15	3.39	11.6%
**Mixed**	1.92	[0.05; 75.12]	0	0	0.58	0.0%
**Permanent**	0.64	[0.26; 1.60]	--	--	--	--
**Overall**	0.81	[0.42; 1.54]	0.28	0.53		53.1%

**Fig 9:**
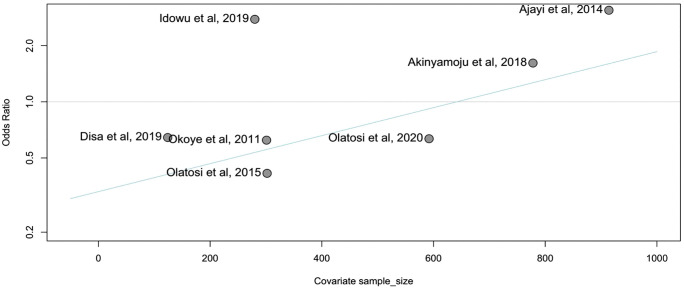
Bubble plot showing the association of tooth brushing methods and sample sizes of studies.

Univariate meta-regression using sample size (p = 0.043) of individual studies showed that study sample sizes were a significant moderating factor in the association between tooth cleaning methods and caries prevalence (Fig 9). R^2^ showed that sample sizes accounted for 79.8% of the study heterogeneity in the included studies.

The year of publication (p = 0.30) and mean age of participants (p = 0.88) had no significant moderating effect on caries prevalence and tooth cleaning methods.

## Discussion

This systematic review and meta-analysis present the first nationally estimated data on the associations between dental caries and oral hygiene practices. The study indicated that oral hygiene status was most commonly measured using the OHI-S, while the prevalence of caries was mainly assessed using the DMFT/dmft indices. However, the metrics for measuring oral hygiene practices were diverse, and indicators lacked uniformity across the included studies. The dental caries experience was higher when oral hygiene was poor, but this relationship was most significant in the primary dentition, and less so in the permanent or mixed dentition. In addition, toothbrushing twice daily or more was associated with a reduction in the odds of having dental caries compared to brushing once daily or less. However, there was no difference in the frequency of dental caries experience among individuals using toothbrushes and toothpaste compared to those using other tooth-cleaning devices.

A strength of the study is the methodological rigor and the transparency of the meta-analytic process, enhancing reproducibility. However, the review has several limitations that should inform the interpretation of findings and guide future research. The cross-sectional design of all included studies precludes causal inference. Unmeasured confounders such as dietary sugar intake, socioeconomic status, fluoride exposure, and access to dental care may influence the observed associations. In addition, there was a significant geographic imbalance, with most studies conducted in Southern Nigeria, particularly the Southwest. This limits the generalizability of findings to Northern Nigeria, where cultural, dietary, and socio-economic contexts may differ substantially. Future studies must prioritize this region to enable nationally representative conclusions. Despite these limitations, the study provides important information.

The first insightful finding from this systematic review and meta-analysis is the inverse relationship between oral hygiene status and the prevalence of dental caries. The relationship is explained by the Ecological Plaque Hypothesis that focuses on biofilm disruption and goes beyond simple sugar removal [66]. The goal of oral hygiene practices is to prevent a detrimental ecological shift in the oral microbiome. In a healthy state, the oral biofilm exists in homeostasis. Beneficial, non‑aciduric bacteria predominate. Salivary flow and buffering capacity help maintain a neutral pH [67]. Frequent consumption of fermentable carbohydrates disrupts this balance. Cariogenic bacteria, notably *Streptococcus mutans*, metabolise sugars to organic acids. This acidifies the environment. It then selects for acidogenic and aciduric species. As pH falls, health‑associated bacteria are inhibited. A self‑perpetuating cycle of acid production and enamel demineralisation begins. This increases the risk for dental caries [68].

Effective oral hygiene restores balance. It physically disrupts the biofilm through toothbrushing and interdental cleaning. This prevents the maturation of a pathogenic ecosystem. It also removes the nutrient source for cariogenic bacteria, thereby suppressing their overgrowth and acid production [69]. Regular disruption further facilitates remineralization: removing the acidic barrier allows saliva to buffer pH, and allows fluoride ions to penetrate enamel, thereby promoting the repair of early carious lesions [70]. The goal is to manage, not eliminate, the biofilm. This steers the microbiome toward a stable, health‑associated composition and keeps cariogenic populations low [67].

The impact of oral hygiene on dental caries experience seems to be highest in the primary dentition. Primary teeth are structurally more vulnerable to rapid dental caries progression than permanent teeth due to key anatomical differences. The broad contact points between molars trap food and plaque, allowing for early caries formation. Their enamel and dentine are thinner and less mineralized, enabling faster penetration by caries [71]. While most studies focus on *Streptococcus mutans* and caries aetiology, the broader plaque microbiome and its ecological shifts (dysbiosis) in response to hygiene and diet in early childhood are an evolving area of study [72]. Further exploration of this association must move beyond reaffirming the link and focus on developing and implementing culturally sensitive, equitable, and evidence-based strategies that disrupt this association at the individual, family, and community levels.

The second insight from this systematic review and meta-analysis is the affirmation that twice-daily brushing is protective from dental caries in the study population. Brushing teeth twice daily enhances plaque control by consistently disrupting the oral biofilm [73,74]. This aligns with the conclusions made in the literature on the importance of regular brushing as a preventive measure against dental caries [73,75]. The observed effect size is, however, small. This extreme point estimate should be interpreted with caution. A conservative interpretation is that twice‑daily brushing is strongly protective, but the precise magnitude of the effect is likely overestimated due to the analytic method and the low baseline caries prevalence in the higher‑frequency brushing group. Future studies with larger sample sizes and standardised exposure categories are needed to obtain a more stable and plausible effect estimate.

The efficacy of twice-daily tooth brushing can be enhanced by considering the optimal timing of brushing, either before or after meals [76,77]. In addition, using dental floss for interdental plaque removal and bacteriostatic mouthwashes can augment the effectiveness of toothbrushing [78–80]. However, none of the included studies reinforced the value of using adjuncts in reducing the dental caries experience, thereby creating a knowledge gap. This gap can be bridged through future studies.

Also, although prior studies had indicated that the complementary use of fluoride-containing toothpaste with toothbrushing can further enhance the efficacy of twice-daily tooth brushing in caries control [81,82], and studies conducted in the country had reinforced this [47,83], the current study did not find such an association. This result should be interpreted cautiously and not as a contradiction of established evidence. Rather, the finding may reflect contextual specificities or methodological limitations. The widespread use of fluoridated toothpaste in Nigeria, with over 90% of the population using it daily [72], likely created a ceiling effect in the primary studies, preventing the detection of a differential effect between comparison groups. The current study finding suggests that in a high-fluoride-toothpaste-use setting, the additional benefit of fluoride toothpaste may be obscured, and that other co-factors, such as dietary habits, may become more salient modifiers of dental caries risk [84].

Beyond oral hygiene practices, other risk factors must be considered, particularly differential exposure to dietary sugars across socioeconomic groups. In Nigeria, a low-middle-income country, a distinct socio-economic gradient influences oral health risk profiles: urban and higher-income populations are experiencing a nutrition transition, characterized by increased consumption of processed foods and sugary snacks and beverages [27] as seen in other transiting economies [85–89]. While these groups typically have better access to toothbrushes, toothpastes and dental service utilisation (leading to better OHI-S scores), their high-frequency exposure to fermentable carbohydrates creates a persistent cariogenic challenge [84,90,91]. Here, even fair oral hygiene may be insufficient to counteract a high-sugar diet, leading to dental caries despite relatively better plaque scores. On the other hand, rural and low-income populations may have more traditional, less processed diets with lower between-meal sugar intake [92]. However, they face barriers to accessing modern oral hygiene tools and preventive dental care [93]. These populations, therefore, have a higher risk for poor oral hygiene due to plaque accumulation from less frequent or less effective cleaning methods. Yet, the lower dietary sugar exposure may result in a less cariogenic oral environment, potentially leading to lower odds of dental caries. This aligns with the substrate limitation principle in cariology.

The current study did not explore the interaction between socioeconomic status, oral hygiene practices, and sugar consumption in relation to dental caries risk, leaving unclear the relative contribution of poor oral hygiene versus high sugar exposure to caries prevalence across Nigerian populations. Future research should therefore adopt a multi‑factorial design that simultaneously quantifies oral hygiene status, dietary sugar intake (both total and between‑meal frequency), and socioeconomic indicators within the same population. Longitudinal cohort studies are also needed to establish temporal relationships and to determine whether improving oral hygiene alone suffices for high‑sugar consumers or whether dietary interventions are essential. Moreover, future studies should validate simple, culturally appropriate dietary assessment tools for Nigerian settings and examine regional variations across the six geopolitical zones, given differences in urbanisation, food environments, and access to dental care.

Third, a culturally significant finding of this meta-analysis is that toothbrushing with toothpaste was not superior to other devices in reducing the risk for caries. A popular tooth cleaning tool in Nigeria is the chewing stick, drawn from plants such as *Salvadora persica* and Neem [94]. It holds deep cultural and religious significance and is traditionally believed to possess cleansing and medicinal properties [95]. The affordability and widespread availability of chewing sticks make them a sustainable alternative to toothbrushes in a resource-limited context. This study finding suggests that effective oral hygiene does not require expensive tools but rather relies on consistency. This raises a future study research question: Does a well-used chewing stick clean more effectively than a poorly used toothbrush? The finding may challenge the longstanding assumption that modern tools are inherently superior and shift the discussion toward a more evidence-based and culturally competent understanding of oral hygiene practices.

For clinicians, this finding may necessitate a shift in counselling that focuses on the appropriate use of toothbrushes. Rather than dismissing alternative oral hygiene practices in favour of the use of toothbrushes, oral health professionals should engage patients with respect, asking what they use, how often, and demonstrate effective techniques for its use. The counselling message should emphasize that the goal is efficient plaque removal, achievable with both toothbrushes and other tooth cleaning tools, while reinforcing the importance of brushing twice daily. For patients reliant on other tooth cleaning tools like chewing sticks, clinicians can encourage proper preparation and use, while complementing them with fluoride exposure through alternatives such as fluoridated mouthwash, where feasible [96]. At the community and public health level, the study evidence supports the need for campaigns that empower rather than prescribe. Messages can build on existing practices rather than attempting to replace them. This culturally sensitive approach acknowledges traditional methods while amplifying the most important public health message about frequency and thoroughness rather than the tool itself, which resonates with cultural realities and socioeconomic conditions. Further studies on using culturally appropriate oral hygiene practices are warranted to enhance our understanding of effective strategies for caries prevention in diverse populations.

Finally, the diversity observed in the array of measures employed to assess oral hygiene practices in the studies included in this systematic review may have contributed to the reported heterogeneity. It is, however, essential to study these assessment tools and adopt context-specific relevant tools for dental caries risk assessment. Adopted tools for use in Nigeria should enable laypersons and healthcare practitioners to screen individuals at risk for dental caries.

On the other hand, we observed that the studies included in this systematic review and meta-analysis assessed dental caries using the DMFT/dmft indices [97], a reliable and user-friendly tool for capturing dental caries experiences, with some limitations to its use [98,99], one of which is its inability to detect enamel caries (caries in their reversible form) [100,101]. Thus, the prevalence of caries would likely be underestimated in the studies included in this meta-analysis. Future research conducted in Nigeria should consider transitioning to tools capable of identifying enamel caries, such as ICDAS, Cardiogram, and CAMBRIA, among others [102].

In conclusion, this study offers a nuanced understanding of oral hygiene practices and their association with the prevalence of dental caries in Nigeria. It affirms that poor oral hygiene status is linked with an increased risk for dental caries, and more so in the primary dentition. It also highlights that twice-daily brushing is protective from dental caries in the study population. This implies that within the Nigerian context, the efficacy of oral hygiene may depend more on the frequency and consistency of the practice than on a specific cleaning device. Future research is needed to demonstrate how to promote behavioral consistency, while being respectful of cultural practices to reduce the burden of dental caries in Nigeria.

## Supporting information

S1 File PRISMA 2020 checklist. (DOCX)

S2 File Search strategy. (DOCX)

S3 Table Table of excluded studies. (XLSX)

S4 Table Assessment of risk of bias. (DOCX)
