# Supplementary material for: Oral hygiene practices and dental caries experience in Nigeria: A systematic review and meta-analysis
Source: PLoS One. 2026 Aug 11;21(8):e0342631. doi: 10.1371/journal.pone.0342631 (PMC13460621; doi:10.1371/journal.pone.0342631)
Supplement: Supplementary file 1 [file pone.0342631.s005.pdf]

PLOS One

Oral Hygiene Practices and Dental Caries Experience in Nigeria: A Systematic Review and Meta-Analysis

--Manuscript Draft--

**Manuscript Number:**	PONE-D-26-04211
**Article Type:**	Research Article
**Full Title:**	Oral Hygiene Practices and Dental Caries Experience in Nigeria: A Systematic Review and Meta-Analysis
**Short Title:**	Oral Hygiene Practices and Dental Caries Experience in Nigeria
**Corresponding Author:**	Adetayo Aborisade<br>Bayero University College of Health Sciences<br>Kano, NIGERIA
**Keywords:**	Oral hygiene practices; dental caries; tooth brushing; Nigeria; Meta-analysis
**Abstract:**	Background: Oral hygiene is linked with dental caries experience. This systematic review and meta-analysis assessed the associations between oral hygiene status, the frequency of tooth brushing, and the prevalence of dental caries in Nigeria.Tools used for maintaining oral hygiene were also identified.<br>Methods: Registered with PROSPERO (CRD42022367763), this review searched PubMed, Web of Science, Scopus, African Journals Online, African Index Medicus, and Google Scholar in January 2026. Observational studies and clinical trials reporting baseline caries prevalence were included. There was no language restriction. Studies were excluded if they did not provide information on the sample size, had no study outcome data, or featured duplicate samples, and if they were review articles, systematic reviews and meta-analyses, case reports, case series, in vitro studies, commentaries/letters (editorials, opinion pieces) devoid of primary data. Pooled odds ratios (ORs) were estimated using random-effects models. Subgroup analyses were conducted by dentition type, geopolitical zone, study design, publication year, mean age, and sample size.<br>Results: Twenty-three cross-sectional studies were included, of which 20 (86.9%) were conducted in Southern Nigeria. After removing an influential outlier, poor oral hygiene was associated with a 38% reduction in caries odds (OR 0.62, 95% CI 0.46–0.84). Brushing at least twice daily was strongly associated with reduced caries (OR 0.01, 95% CI 0.00–0.01). No significant association was found between the type of cleaning device and caries prevalence. Subgroup analyses identified dentition type and publication year as significant moderators. Heterogeneity ranged from low to moderate, and no publication bias was detected for primary associations. The most common cleaning tool was a toothbrush with toothpaste, though chewing sticks, cotton wool, and other traditional tools were also reported.<br>Conclusion: Twice-daily tooth brushing is strongly associated with lower caries prevalence in Nigeria. Poor oral hygiene was linked to reduced caries odds in pooled analysis, a finding that may reflect socio-economic and dietary confounding. The type of cleaning tool was not significantly associated with caries risk, highlighting the importance of brushing frequency and technique over tool type. Future research should prioritize Northern Nigeria to address the geographic skewness of the data and improve national representativeness.
**Order of Authors:**	Adetayo Aborisade
	Amina Mohammed Ali
	Chizoba Okolo
	Titilola Abike Gbaja-Biamila
	Folahanmi Tomiwa Akinsolu
	Abideen Olurotimi Salako
	Ifeoluwa Eunice Adewole
	Mobolaji Timothy Olagunju

Powered by Editorial Manager® and ProduXion Manager® from Aries Systems Corporation

	Olunike Rebecca Abodurin
	George Uchenna Eleje
	Ucheoma Catherine Nwaozuru
	Adebola Oluyemisi Ehizele
	Joanne Marie Lusher
	Maha El Tantawi
	Morẹ́nikẹ́ Oluwátóyìn Foláyan
**Additional Information:**	
**Question**	**Response**
**Financial Disclosure**<br><br>Enter a financial disclosure statement that describes the sources of funding for the work included in this submission. Review the submission guidelines for detailed requirements. View published research articles from *PLOS ONE* for specific examples.<br><br>This statement is required for submission and **will appear in the published article** if the submission is accepted. Please make sure it is accurate.<br><br>**Funded studies**<br>Enter a statement with the following details:<br>• Initials of the authors who received each award<br>• Grant numbers awarded to each author<br>• The full name of each funder<br>• URL of each funder website<br>• Did the sponsors or funders play any role in the study design, data collection and analysis, decision to publish, or preparation of the manuscript?<br><br>Did you receive funding for this work?	Yes
Please add funding details.<br>as follow-up to "**Financial Disclosure**<br><br>Enter a financial disclosure statement that describes the sources of funding for the work included in this submission. Review the submission guidelines for detailed requirements. View published research articles from *PLOS ONE* for specific	The study received a funding with Grant Number: 5NM-ADJGT-22-0082 of $130.00 from the Nigerian Institute for Medical Research. The funders play any role in the study design, data collection and analysis, decision to publish, or preparation of the manuscript?

examples.<br><br>This statement is required for submission and **will appear in the published article** if the submission is accepted. Please make sure it is accurate.<br><br>**Funded studies**<br>Enter a statement with the following details:<br>• Initials of the authors who received each award<br>• Grant numbers awarded to each author<br>• The full name of each funder<br>• URL of each funder website<br>• Did the sponsors or funders play any role in the study design, data collection and analysis, decision to publish, or preparation of the manuscript?<br><br>Did you receive funding for this work?"	
Please select the country of your main research funder (please select carefully as in some cases this is used in fee calculation).<br>as follow-up to "**Financial Disclosure**<br><br>Enter a financial disclosure statement that describes the sources of funding for the work included in this submission. Review the submission guidelines for detailed requirements. View published research articles from *PLOS ONE* for specific examples.<br><br>This statement is required for submission and **will appear in the published article** if the submission is accepted. Please make sure it is accurate.	NIGERIA - NG

**Funded studies**<br>Enter a statement with the following details:<br>• Initials of the authors who received each award<br>• Grant numbers awarded to each author<br>• The full name of each funder<br>• URL of each funder website<br>• Did the sponsors or funders play any role in the study design, data collection and analysis, decision to publish, or preparation of the manuscript?<br><br>Did you receive funding for this work?"	
**Competing Interests**<br><br>Use the instructions below to enter a competing interest statement for this submission. On behalf of all authors, disclose any competing interests that could be perceived to bias this work—acknowledging all financial support and any other relevant financial or non-financial competing interests.<br><br>This statement is required for submission and **will appear in the published article** if the submission is accepted. Please make sure it is accurate and that any funding sources listed in your Funding Information later in the submission form are also declared in your Financial Disclosure statement.<br><br>View published research articles from *PLOS ONE* for specific examples.	The authors have declared that no competing interests exist

**NO authors have competing interests**<br><br>Enter: *The authors have declared that no competing interests exist.*<br><br>**Authors with competing interests**<br><br>Enter competing interest details beginning with this statement:<br><br>*I have read the journal's policy and the authors of this manuscript have the following competing interests: [insert competing interests here]*<br><br>* typeset	
**Ethics Statement**<br><br>Enter an ethics statement for this submission. This statement is required if the study involved:<br><br>• Human participants<br>• Human specimens or tissue<br>• Vertebrate animals or cephalopods<br>• Vertebrate embryos or tissues<br>• Field research<br><br>Write "N/A" if the submission does not require an ethics statement.<br><br>General guidance is provided below. Consult the submission guidelines for detailed instructions. **Make sure that all information entered here is included in the Methods section of the manuscript.**	N/A

**Format for specific study types**<br><br>**Human Subject Research (involving human participants and/or tissue)**<br>• Give the name of the institutional review board or ethics committee that approved the study<br>• Include the approval number and/or a statement indicating approval of this research<br>• Indicate the form of consent obtained (written/oral) or the reason that consent was not obtained (e.g. the data were analyzed anonymously)<br><br>**Animal Research (involving vertebrate animals, embryos or tissues)**<br>• Provide the name of the Institutional Animal Care and Use Committee (IACUC) or other relevant ethics board that reviewed the study protocol, and indicate whether they approved this research or granted a formal waiver of ethical approval<br>• Include an approval number if one was obtained<br>• If the study involved *non-human primates*, add *additional details* about animal welfare and steps taken to ameliorate suffering<br>• If anesthesia, euthanasia, or any kind of animal sacrifice is part of the study, include briefly which substances and/or methods were applied<br><br>**Field Research**<br><br>Include the following details if this study involves the collection of plant, animal, or other materials from a natural setting:<br>• Field permit number<br>• Name of the institution or relevant body that granted permission	
**Data Availability**<br><br>Authors are required to make all data underlying the findings described fully available, without restriction, and from the time of publication. PLOS allows rare exceptions to address legal and ethical concerns. See the PLOS Data Policy and FAQ for detailed information.	No - some restrictions will apply

Powered by Editorial Manager® and ProduXion Manager® from Aries Systems Corporation

A Data Availability Statement describing where the data can be found is required at submission. Your answers to this question constitute the Data Availability Statement and **will be published in the article**, if accepted.<br><br>**Important:** Stating 'data available on request from the author' is not sufficient. If your data are only available upon request, select 'No' for the first question and explain your exceptional situation in the text box.<br><br>Do the authors confirm that all data underlying the findings described in their manuscript are fully available without restriction?	
**Describe where the data may be found in full sentences. If you are copying our sample text, replace any instances of XXX with the appropriate details.**<br><br>• If the data are **held or will be held in a public repository**, include URLs, accession numbers or DOIs. If this information will only be available after acceptance, indicate this by ticking the box below. For example: *All XXX files are available from the XXX database (accession number(s) XXX, XXX.*).<br>• If the data are all contained **within the manuscript and/or Supporting Information files**, enter the following: *All relevant data are within the manuscript and its Supporting Information files.*<br>• If neither of these applies but you are able to provide **details of access elsewhere**, with or without limitations, please do so. For example:<br><br>*Data cannot be shared publicly because of [XXX]. Data are available from the XXX Institutional Data Access / Ethics Committee (contact via XXX) for researchers who meet the criteria for access to confidential data.*<br><br>*The data underlying the results presented in the study are available from (include the name of the third party*	All datasets generated and analyzed, including the study protocol, search strategy, list of included and excluded studies, data extracted, analysis plans, and quality assessment, are available in the article and upon request from the corresponding author.

and contact information or URL).<br>• This text is appropriate if the data are owned by a third party and authors do not have permission to share the data.<br><br>* typeset	
Additional data availability information:	

Powered by Editorial Manager® and ProduXion Manager® from Aries Systems Corporation

Manuscript

Click here to access/download;Manuscript;Manuscript_Oral Hygiene_PLOS.docx

Oral Hygiene Practices and Dental Caries Experience in Nigeria: A Systematic Review and Meta-Analysis

Adetayo Aborisade[1], Amina Mohammed Ali[2], Chizoba Okolo[3], Titilola Abike Gbaja-Biamila[4,5], Folahanmi Tomiwa Akinsolu[4,5,6], Abideen Olurotimi Salako[4,5,6], Ifeoluwa Eunice Adewole[4,6] Mobolaji Timothy Olagunju[7], Olunike Rebecca Abodurin[4,6,7,8] George Uchenna Eleje[4,9,10,11] Ucheoma Catherine Nwaozuru,[4,12] Adebola Oluyemisi Ehizele[4,13], Joanne Marie Lusher[4,14] Maha El Tantawi,[4,15] Oliver Chukwujekwu Ezechi[4,5,6], Morẹ́nikẹ́ Oluwátóyìn Foláyan[4,16]

1. Department of Oral Diagnostic Sciences, Bayero University, Kano, Nigeria
2. Department of Oral Maxillofacial Surgery, Bayero University, Kano, Nigeria
3. Department of Child Dental Health, Bayero University, Kano, Nigeria
4. Oral Health Initiative, Center for Reproductive and Population Health Studies, Nigerian Institute of Medical Research, Yaba, Lagos, Nigeria
5. Department of Clinical Sciences, Nigerian Institute of Medical Research, Lagos
6. Department of Public Health, Faculty of Basic Medical and Health Sciences, Lead City University, Ibadan
7. Department of Epidemiology and Health Statistics, Nanjing Medical University, China
8. Lagos State Health Management Agency, Lagos
9. Department of Obstetrics and Gynaecology, Nnamdi Azikiwe University Teaching Hospital, Nnewi, Nigeria
10. Effective Care Research Unit, Department of Obstetrics and Gynaecology, Nnamdi Azikiwe University, Awka, Nigeria
11. Department of Community Medicine and Primary Healthcare, Nnamdi Azikiwe University Teaching Hospital, Nnewi, Nigeria
12. Wake Forest School of Medicine, North Carolina, USA
13. Department of Periodontics, School of Dentistry, College of Medical Sciences, University of Benin, Benin City, Nigeria
14. Provost's Group, Regent's University London, UK
15. Department of Pediatric Dentistry and Dental Public Health. Faculty of Dentistry, Alexandria University, Egypt.
16. Department of Child Dental Health, Obafemi Awolowo University, Ile-Ife, Nigeria

Corresponding author:
Adetayo Aborisade
Department of Oral Diagnostic Sciences,
Bayero University, Kano, Nigeria
adetayoaborisade@gmail.com

Clinical trial number: Not applicable

Abstract

Background: Oral hygiene is linked with dental caries experience. This systematic review and meta-analysis assessed the associations between oral hygiene status, the frequency of tooth brushing, and the prevalence of dental caries in Nigeria. Tools used for maintaining oral hygiene were also identified.

Methods: Registered with PROSPERO (CRD42022367763), this review searched PubMed, Web of Science, Scopus, African Journals Online, African Index Medicus, and Google Scholar in January 2026. Observational studies and clinical trials reporting baseline caries prevalence were included. There was no language restriction. Studies were excluded if they did not provide information on the sample size, had no study outcome data, or featured duplicate samples, and if they were review articles, systematic reviews and meta-analyses, case reports, case series, in vitro studies, commentaries/letters (editorials, opinion pieces) devoid of primary data. Pooled odds ratios (ORs) were estimated using random-effects models. Subgroup analyses were conducted by dentition type, geopolitical zone, study design, publication year, mean age, and sample size.

Results: Twenty-three cross-sectional studies were included, of which 20 (86.9%) were conducted in Southern Nigeria. After removing an influential outlier, poor oral hygiene was associated with a 38% reduction in caries odds (OR 0.62, 95% CI 0.46–0.84). Brushing at least twice daily was strongly associated with reduced caries (OR 0.01, 95% CI 0.00–0.01). No significant association was found between the type of cleaning device and caries prevalence. Subgroup analyses identified dentition type and publication year as significant moderators. Heterogeneity ranged from low to moderate, and no publication bias was detected for primary associations. The most common cleaning tool was a toothbrush with toothpaste, though chewing sticks, cotton wool, and other traditional tools were also reported.

Conclusion: Twice-daily tooth brushing is strongly associated with lower caries prevalence in Nigeria. Poor oral hygiene was linked to reduced caries odds in pooled analysis, a finding that may reflect socio-economic and dietary confounding. The type of cleaning tool was not significantly associated with caries risk, highlighting the importance of brushing frequency and technique over tool type. Future research should prioritize Northern Nigeria to address the geographic skewness of the data and improve national representativeness.

Keywords: Oral hygiene practices, dental caries, tooth brushing, Nigeria, meta-analysis

Introduction

Oral hygiene is essential for oral and general health. It encompasses a range of preventative practices that individuals adopt to maintain the cleanliness and overall health of the oral cavity. [1-3]. Key oral hygiene methods include regular tooth brushing with a toothbrush and toothpaste, which removes dental plaque and food particles, prevents periodontal diseases, and dental caries. [4]. Other practices, such as dental floss or interdental cleaning, antimicrobial rinses, and tongue cleaning, complement tooth brushing in controlling plaque accumulation and maintaining oral health. [5, 6]. .Furthermore, oral hygiene includes thorough cleaning of the dorsum of the tongue using a tongue scraper or a toothbrush, which helps to eliminate bacteria and debris from the oral cavity because the tongue can be a reservoir for oral bacteria [7-9].

The link between oral hygiene and dental caries is well-established, as dental plaque plays a central role in initiating and advancing dental caries lesions [10, 11], which suggests that efforts to control plaque would positively affect the prevention of dental caries [12]. Regular toothbrushing, fluoridated toothpaste, and dental floss help remove plaque and reduce the availability of fermentable carbohydrates for bacterial acid production. [6, 13-15]. Oral hygiene practices can reduce the risk of developing dental caries when combined with professional dental care. [11, 14, 16, 17]. Proper oral hygiene removes plaque from tooth surfaces. [10, 18], which prevents the initiation of enamel demineralization caused by acid produced by bacterial fermentation of carbohydrates in the oral cavity [3, 19].

Globally, untreated dental caries in permanent teeth remains the most prevalent health condition, affecting an estimated two billion people in 2022 [20]. Despite this burden, there is limited context-specific evidence from Nigeria. The most recent national survey on dental caries in Nigeria was conducted in 1995, and a national survey on oral hygiene practices was last conducted in 2014 [21] Since then, major socio-economic and demographic shifts, urbanization, dietary changes (including increased sugar consumption), and disparities in access to preventive oral health services have likely altered the relationship between oral hygiene practices and dental caries. Furthermore, regional diversity in cultural practices, such as the use of chewing sticks alongside toothbrushes, necessitates evidence that reflects both modern and traditional practices. Without updated, nationally representative evidence, policymakers and oral health professionals lack the

data needed to design effective, culturally relevant, and equitable interventions for dental caries prevention.

In the absence of national surveillance, a systematic review can address the critical evidence gap and offer insights to guide oral health policies, prevention strategies, and public health programs in line with the United Nations Sustainable Development Goal 3 on good health and well-being [22]. This systematic review and meta-analysis therefore aimed to synthesize available evidence on the association between oral hygiene practices and dental caries in Nigeria and to provide a national estimate from published observational studies.

Methods

Study protocol

This systematic review and meta-analysis were initially registered with PROSPERO (CRD42022367763) in 2022 and updated on the 24th of January 2026. The study was reported in accordance with the Preferred Reporting Items for Systematic Reviews and Meta-analyses (PRISMA) statement and checklist. [23]. Two blinded reviewers performed each review stage, and disagreements were resolved through discussions with a third reviewer.

Research questions

The following research questions guided the study: 1) Is there an association between oral hygiene status and dental caries? 2) Is there an association between the frequency of tooth brushing and the prevalence of dental caries? 3) What tools were used by participants for maintaining oral hygiene?

Search strategy

Five databases (PubMed, Web of Science, Scopus, African Journals Online, and African Index Medicus) and Google Scholar were searched for relevant articles published with no language restriction. The initial search syntax was developed for PubMed and later adapted to fulfill the unique search criteria of the other databases (Supplemental File 1).

To ensure a comprehensive search, grey literature was sought from the libraries of the National Postgraduate Medical College of Nigeria and the West African College of Surgeons. In addition,

the reference lists of all retrieved articles, systematic reviews, and meta-analyses were examined. To identify further sources, study investigators were contacted directly for inaccessible publications and for clarification on missing data, theses, and unpublished manuscripts.

Inclusion and Exclusion Criteria

All published and unpublished studies, including hand searches conducted in Nigeria between January 2001 and December 2025, that reported on associations between oral hygiene and the prevalence of dental caries were eligible for study inclusion. Study designs eligible for inclusion were cross-sectional, cohort, and case-control studies. Clinical trials that provided relevant baseline prevalence data were also included. Studies were also included if they presented available data for at least one of the primary outcomes indicated in Table 1, whose reported odds ratio (OR) and 95% confidence interval (CI) were analyzed by univariate or multivariate analyses, and studies whose data could be analyzed to generate variables for the current study.

Studies were excluded if they did not provide information on the sample size, had unavailable outcome data, or featured duplicate samples. Review articles, systematic reviews and meta-analyses, case reports or case series, in vitro studies, commentaries/letters (editorials, opinion pieces), devoid of primary data, were excluded. Studies with overlapping data from other included studies were also excluded. Excluded studies are reported in Supplemental File 2.

Selection of studies

Studies were screened using the PECOS framework (Population, Exposure Comparators, Outcomes, Time, Studies). Table 1 presents the PECOS framework used for this study. [24]. In this review, dental caries was the primary outcome of interest. Oral hygiene practices were considered the exposures, and different categories of practices served as comparators. This distinction was made explicit to ensure clarity in the analytic framework and to avoid misinterpretation of dental caries as an exposure or risk factor.

Table 1: Eligibility Criteria using PECOS (Population, Exposure, Comparisons, Outcomes, Studies) framework

Population	Individuals residing in Nigeria
Exposure	Oral hygiene practices (tooth brushing frequency and tooth cleaning methods - toothbrush, chewing stick, or other devices), and oral hygiene status.
Comparators	Variations in oral hygiene practices (e.g., brushing ≥2 times/day vs. once/day; good vs. poor hygiene; toothbrush vs. chewing stick).
Outcomes	Presence of dental caries.
Studies	Studies reporting prevalence data.

Three authors (AA, AMA, and CO) independently reviewed the titles and abstracts of each study that met the inclusion criteria after removing duplicates and downloading them to the reference management software EndNote 7.8. Studies that did not meet the inclusion criteria and those where the full text was unavailable were excluded. Two reviewers (AA and AMA) independently assessed the eligibility of the retrieved manuscripts, and any disagreements were resolved by discussion or recourse to a third reviewer (MOF).

Data Extraction

Four independent reviewers (AA, TAG, IEA, and ORA) used a pretested data extraction form prepared in Microsoft Excel to independently extract information related to the author's name and year of publication. In addition, specific details about the study design, location, and study setting were captured. Details about the study participants – sample size, age distribution, sex of participants, and other unique characteristics – were extracted. Information about participants' oral hygiene practices was also extracted. These details included measures of oral hygiene status, frequency and duration of hygiene practices, and the tools or methods employed for oral hygiene. Simultaneously, dental caries assessment data were cataloged, highlighting each study's tools or methods for assessing dental caries. The percentage of participants with dental caries was also extracted. Lastly, the results or association measures, whether in the form of prevalence or statistical ratios indicating the associations between oral hygiene practices and dental caries, were recorded. Any discrepancies were resolved by a fifth reviewer (MOF).

Quality and Risk of Bias Assessment

Four independent reviewers (AA, TAG, IEA, and ORA) assessed the methodological quality and risk of bias in the included studies, with discrepancies resolved by a fifth reviewer (FTA) using an adapted version of the risk of bias tool for prevalence studies according to the modified Joana Briggs Institute Assessment for Risk of Bias [25]. This risk of bias tool, designed for prevalence studies, was applied as the most appropriate quality assessment approach because the Odds Ratios (ORs) synthesized in the meta-analysis were calculated directly from raw prevalence counts (number of events and non-events) reported in the included cross-sectional studies. The validity of these ORs is fundamentally dependent on the methodological quality of the prevalence data. The total score ranged from 0 to 9, with the overall score categorized as follows: 0–3: "high risk," 4–6: "moderate risk," and 7–9: "low risk" of bias.

Certainty of evidence

The certainty of evidence for each outcome using the Grading of Recommendations Assessment, Development, and Evaluation (GRADE) approach was not conducted because, under this framework, cross-sectional studies are rated as low certainty evidence [26, 27]. The data included in this study were all cross-sectional in design.

Sensitivity analysis

A sensitivity analysis was planned to be performed with leave-one-out analysis to investigate the impact of risk of bias on the test of associations and to uncover a potential source of heterogeneity.

Assessment for publication bias

Symmetry of the funnel plot was planned to assess the publication bias of the articles included in the systematic review. Egger's regression test was also utilized to assess publication bias objectively with significance at $p<0.05$.

Statistical Analysis

All analyses were conducted using R and RStudio Version 4.5.1 ("Great Square Root"). A descriptive analysis was first undertaken to show the distribution of studies by year of publication, study design, geopolitical region, number of male and female participants, and type of dentition studied: primary (0 –5 years), mixed (6–11 years), and permanent (≥12 years). We also described

the number of studies reporting an association between oral hygiene practices and dental caries prevalence by geographic region and dentition type.

This meta-analysis applied strict inclusion criteria, selecting only studies that reported definitive, binary data on the presence or absence of dental caries. This ensured an objective and direct assessment of the relationship between oral hygiene practices and caries, aligning with standard epidemiological methods for evaluating disease prevalence and risk factors. Pooled estimates were generated using the Mantel-Haenszel method. Heterogeneity was evaluated using Cochran's Q test, the I^2 statistic, and inspection of forest plots. The independent variables were oral hygiene practices (tooth cleaning method, brushing frequency, and oral hygiene status), while the dependent variable was dental caries prevalence. Odds ratios (ORs) with 95% confidence intervals (CIs) were used as the summary measure, categorizing outcomes into dental caries present versus absent. These ORs were calculated from extracted frequencies and denominators reported in the studies, rather than adopting published effect sizes. Because the synthesis was based on prevalence data, a risk of bias tool designed for prevalence studies was applied as the most appropriate quality assessment approach. A p-value <0.05 for the Q test was considered statistically significant, and I^2 values above 50% indicated substantial heterogeneity. Negative I^2 values were treated as zero [28, 29], following Cochrane Handbook guidance [30, 31]. Studies with no events in either arm were excluded from the meta-analysis. [29-32].

Associations between oral hygiene practices and dental caries prevalence were evaluated using moderator analysis, including subgroup analysis and univariate meta-regression. When study heterogeneity is high, subgroup analyses were conducted based on the type of dentition (primary, mixed, permanent), geopolitical zone (South and Northern), and study design (population-based, school-based, or other). Univariate meta-regression examined the year of publication, study sample size, and participants' mean age in each study. Sensitivity analyses involved excluding studies with a high risk of bias and sequentially removing individual studies to assess their influence on pooled estimates. Publication bias was assessed using funnel plots and Egger's test when at least ten studies reported the same outcome [33, 34].

Results

Selection of studies

As shown in Figure 1, 1422 records were retrieved. After removing 414 duplicates, 1008 records remained for eligibility screening. Of these, 935 studies were excluded based on their titles and abstracts. After reviewing the full-text records, 23 met the inclusion criteria [5, 8, 15, 18, 35-53].

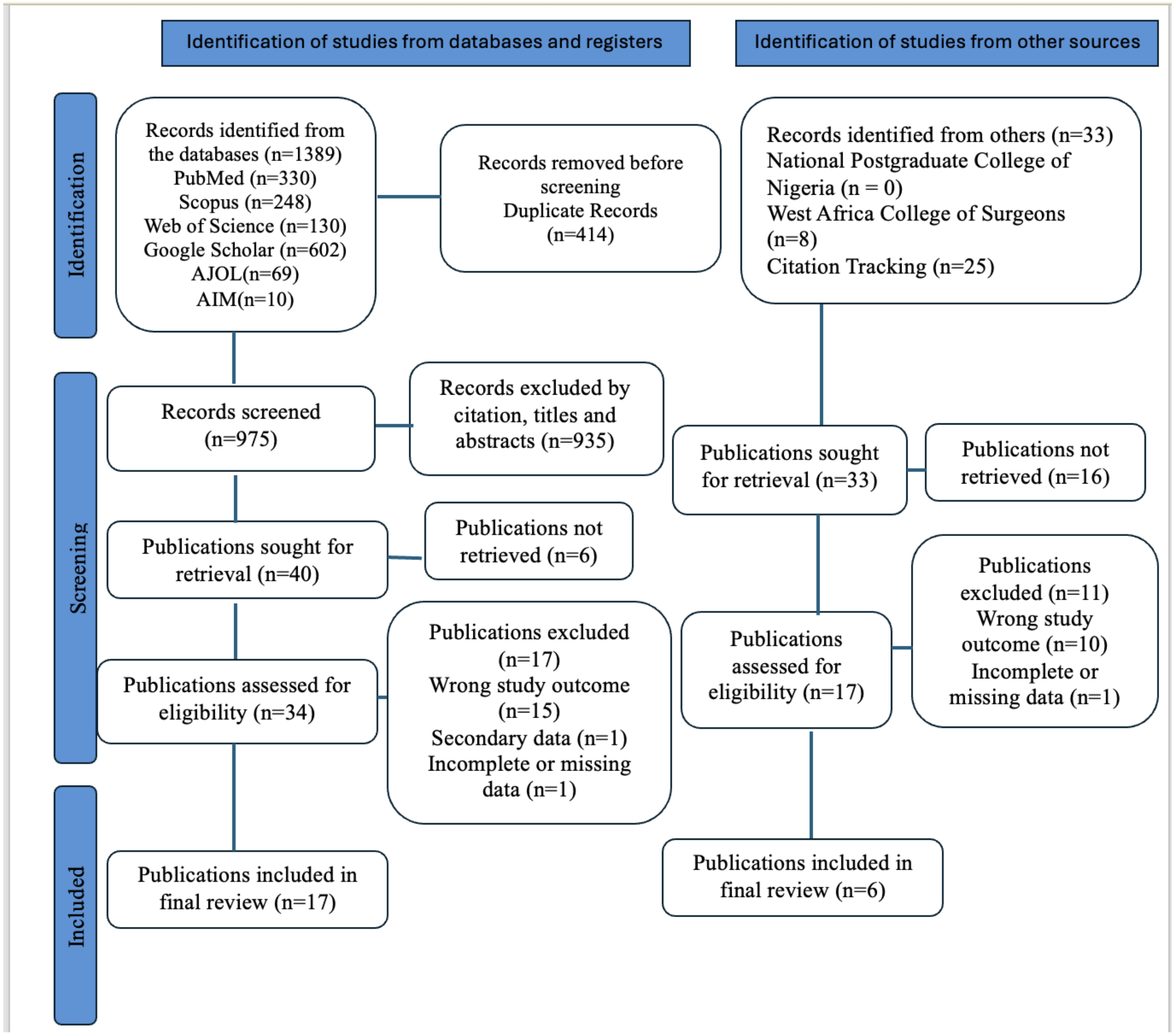

Figure 1: PRISMA flow diagram for the search results

Characteristics of included studies

The characteristics of the 23 included studies are detailed in Table 2. The participant population size in each study ranged from 124 to 2,107 individuals, culminating in a combined total of 15,797

participants. The aggregated sample comprised 7838 males and 7959 females with participants' ages ranging from six months to 64 years.

The publication timeline of the included studies spans 16 years, from 2007 to 2023, and can be categorized into distinct periods. (1) Early Period (2007-2015): Six studies (26.1%) were published during this foundational phase [8, 15, 35, 36, 37, 38]. (2) Middle Period (2016-2020): Ten studies (43.5%) were published, indicating an increase in research activity [5, 18, 39, 40, 41, 42, 43, 44, 45, 46]. (3) Recent Period (2021-2023): Seven studies (30.4%) were published in the most recent years, demonstrating sustained interest in the topic [47, 48, 49, 50, 51, 52, 53]. All 23 studies employed an observational design. Twenty-one studies (91.3%) were cross-sectional surveys [5, 8, 15, 18, 32- 42, 44-52], while two studies (8.7%) utilized a case-control design [43, 53].

Geographically, the studies showed a significant concentration in Southern Nigeria. Twenty studies (86.9%) were conducted in the southern region, compared to three studies (13.1%) from Northern Nigeria. Within Southern Nigeria, the Southwest zone was the most researched, with 16 studies (69.6% of the total). These were conducted across Lagos [15, 38, 41, 44, 46], Osun [5, 37, 40, 45, 47, 50, 51, 53], Ogun [18, 50], 52], and Oyo (Ibadan) [36] states. Three studies (13.0%) were conducted in the Southeast (Enugu) [35, 39, 48], and one study (4.3%) was from the South-South (Rivers) region [8]. In Northern Nigeria, two studies were from the Northeast (Bauchi and Yobe) [42, 43], and one study was from the Northcentral (Jos) zone [49].

Table 2: Characteristics of Included Studies

S/No	Study Reference	Title	Study Design	City/State	Region	Sample Size	Age Range	Gender Distribution (M/F)
1	Sowole et al., 2007 [15]	Dental Caries Pattern and Predisposing Oral Hygiene Related Factors in Nigerian Preschool Children	Cross-sectional	Lagos	Southwest	389	6-71 months	217 / 172
2	Okoye et al., 2011 [35]	Prevalence of Dental Caries in a Nigerian Rural Community: A Preliminary Local Survey	Cross-sectional	Enugu	Southeast	301	11-16 years	100 / 201
3	Ajayi and Abidemi-Solanke, 2014 [36]	Socio-behavioural risk factors of dental caries among selected adolescents in Ibadan, Nigeria	Cross-sectional	Ibadan	Southwest	914	10-19 years	451 / 463
4	Folayan et al., 2015 [37]	Prevalence And Early Childhood Caries Risk Indicators in Preschool Children in Suburban Nigeria	Cross-sectional	Osun	Southwest	497	6-71 months	266 / 231
5	Olatosi et al., 2015 [38]	The Prevalence of Early Childhood Caries and Its Associated Risk Factors Among Preschool Children Referred to a Tertiary Institution	Cross-sectional	Lagos	Southwest	202	6-71 months	44 / 158
6	Olabisi et al., 2015 [8]	Prevalence Of Dental Caries and Oral Hygiene Status Of A Screened Population in Port Harcourt, Rivers State, Nigeria	Cross-sectional	Rivers	South-South	288	15-64 years	133 / 155
7	Onyejaka and Amobi, 2016 [39]	Risk Factors of Early Childhood Caries among Children in Enugu, Nigeria	Cross-sectional	Enugu	Southeast	429	9 months - 5 years	226 / 203
8	Akinyamoju et al., 2018 [18]	Dental Caries and Oral Hygiene Status: Survey of Schoolchildren in Rural Communities, Southwest Nigeria	Cross-sectional	Ogun	Southwest	778	7-17 years	424 / 354
9	Oyedele et al., 2018 [40]	Impact of Oral Hygiene and Sociodemographic Factors on Dental Caries in a Suburban Population in Nigeria	Cross-sectional	Osun	Southwest	2,107	8-16 years	982 / 1125
10	Kolawole et al., 2019 [5]	Association Between Malocclusion, Caries, And Oral Hygiene in Children 6 To 12 Years Old Residents in Suburban Nigeria	Cross-sectional	Osun	Southwest	495	6-12 years	242 / 253
11	Abiola et al., 2019 [41]	Dental Caries Occurrence and Associated Oral Hygiene Practices Among Rural and Urban Nigerian Preschool Children	Cross-sectional	Lagos	Southwest	404	18-60 months	208 / 196

S/No	Study Reference	Title	Study Design	City/State	Region	Sample Size	Age Range	Gender Distribution (M/F)
12	Nwathor et al., 2019 [42]	Oral Hygiene Status, Practices, and Awareness of Medium Security Prison Inmates in Northeastern Nigeria	Cross-sectional	Bauchi	Northeast	280	<30->50 years	276 / 4
13	Disa et al., 2019 [43]	Predictors of dental caries among adults and adolescents in a dental clinic in North-eastern Nigeria	Case-control	Yobe	Northeast	124	15-63 years	59 / 65
14	Oyapero et al., 2020 [44]	Association Between Dental Caries, Odontogenic Infections, Oral Hygiene Status, and Anthropometric Measurements of Children in Lagos, Nigeria	Cross-sectional	Lagos	Southwest	278	1-15 years	132 / 146
15	Arowolo, 2020 [45]	Determination Of the Association Between Nutritional Status and Dental Caries In 6-16-Year-Old School Children In Ile – Ife	Cross-sectional	Osun	Southwest	1,502	6-16 years	689 / 813
16	Olatosi et al., 2020 [46]	Disparities in Caries Experience and Socio-Behavioural Risk Indicators Among Private School Children in Lagos, Nigeria	Cross-sectional	Lagos	Southwest	592	5-16 years	307 / 285
17	Folayan et al., 2021 [47]	Associations Between a History of Sexual Abuse and Dental Anxiety, Caries Experience, and Oral Hygiene Status Among Adolescents in Sub-Urban South West Nigeria	Cross-sectional	Osun	Southwest	1,056	10-19 years	598 / 458
18	Onyejaka et al., 2021 [48]	Prevalence and Associated Factors of Dental Caries Among Primary School Children in Southeast Nigeria	Cross-sectional	Enugu	Southeast	657	5-17 years	316 / 341
19	Idowu et al., 2021 [49]	Dental Caries Prevalence, Restorative Needs and Oral Hygiene Status in Adult Population: A Cross-sectional Study among Nurses in Jos University Teaching Hospital, Jos, Nigeria	Cross-sectional	Jos	Northcentral	251	9–64 years	77 / 174
20	Oyedele et al., 2021 [50]	Comparison of Dental Caries and Oral Hygiene Status of Children in Suburban areas with those in the Rural Population of Southwestern Nigeria	Cross-sectional	Osun/Ogun	Southwest	1,397	8-12 years	706 / 691
21	Folayan et al., 2021 [51]	Individual and familial factors associated with caries and gingivitis among adolescents resident in a semi-urban community in South Western Nigeria	Cross-sectional	Osun	Southwest	1,472	10-19 years	846 / 626

S/No	Study Reference	Title	Study Design	City/State	Region	Sample Size	Age Range	Gender Distribution (M/F)
22	Oyedele et al., 2022 [52]	Associations Between Dental Caries, Oral Hygiene Status, And Oral Health Practices of First-Year Undergraduates in A Private University in Nigeria	Cross-sectional	Ogun	Southwest	1,164	15-23 years	443 / 721
23	Afolabi, 2023 [53]	Comparative assessment of the oral health status of children aged 4-16 years with and without sickle cell anaemia resident in Ile-Ife and Ilesha, Osun State, Nigeria	Case-control	Osun	Southwest	220	6-16 years	96 / 124

Oral hygiene status, frequency, and devices used for oral hygiene

Oral Hygiene Status Assessment

Oral hygiene status was assessed in 14 (60.9%) of the 23 studies for association with the prevalence of dental caries. The Simplified Oral Hygiene Index (OHI-S) was the predominant tool, used in 12 (85.7%) of the studies [5, 15, 36, 37, 39, 40, 41, 48, 49, 50, 52, 53], and one study used the Plaque Index [51]. Most studies that used the OHI-S reported the distribution of participants across categories of good, fair, and poor oral hygiene. One study did not specify its tool for OHI measurement [40], while another presented mean OHI-S scores stratified by urban/rural setting, gender, and age [41].

Measures of Oral Hygiene Practices

Studies assessed various dimensions of oral hygiene behaviour:

1. **Frequency of Mouth Cleaning:** Reported in 17 studies [15, 18, 35 - 39, 41 - 43, 45 – 47, 49, 51 - 53]. The reported frequencies ranged from 'none' [15, 39], rarely [42], and 'occasionally' [18, 35] to once daily, twice daily, and more than twice daily [15, 18, 34, 35, 36, 37, 38, 40, 41, 42, 44, 45, 46, 48, 50, 51, 52].
2. **Devices Used for Mouth Cleaning:** Documented in 14 studies [15, 18, 35, 36, 38, 41, 42, 43, 45, 47, 49, 51, 52, 53]. A wide array of devices was reported, including toothbrushes with toothpaste (most common), chewing sticks, cotton wool, dental floss, gauze, fingers, herbs, and traditional agents like glycerine or ground glass.
3. **Other Behavioural Variables:** Several studies explored additional factors, including the individual responsible for cleaning (child or caregiver) [38], supervision of brushing [38, 41], age at onset of oral cleaning [38], timing of cleaning (e.g., before/after meals) [42, 49, 52], and the frequency of changing cleaning devices [18, 41, 43].

Dental Caries Assessment

All 23 studies used the DMFT/dmft indices to assess caries prevalence and severity. Two studies additionally employed the PUFA/pufa index to measure the clinical consequences of untreated caries [5, 43]. The assessed dentitions were: primary dentition (7 studies) [15, 37, 38, 39, 41, 45, 53], permanent dentition (9 studies) [8, 35, 42, 43, 47, 49, 51, 52, 53], and mixed dentition (8 studies) [5, 18, 36, 40, 44, 46, 48, 50]. The reported caries prevalence ranged widely from 2.2% to 79.1%.

Association between Oral Hygiene Status and Dental Caries

Of the 19 studies that assessed oral hygiene status, 14 (73.7%) investigated its association with caries [5, 15, 36, 37, 39, 40, 41, 44, 48, 49, 50, 51, 52, 53]. A consistent finding was that poor oral hygiene status was significantly associated with a higher prevalence or severity of caries in eight of these studies [5, 15, 39, 40, 44, 45, 52, 53]. For example, poor oral hygiene increased the odds of caries by factors ranging from 1.8 to

15.5 [5, 45, 52]. Conversely, good [48] and fair [39, 40, 48] oral hygiene were associated with a lower risk for caries in several studies. Fair oral hygiene was also associated with increased odds of caries [40, 52]. Some studies reported a dose-response relationship, where the risk of caries increased with higher (worse) OHI-S scores [5, 41]. Four studies found no significant association [36, 49, 51, 53].

Association between Oral Hygiene Practices and Dental Caries

The evidence for associations between specific oral hygiene behaviours and caries was less consistent:

- **Frequency of Cleaning:** Most studies (8/10) found no significant association between brushing frequency (e.g., once vs. twice daily) and caries prevalence [18, 35, 36, 42, 46, 47, 51, 53]. Two studies reported protective effects for brushing at least twice daily [43, 45].
- **Cleaning Devices:** The use of a toothbrush with fluoride toothpaste was generally associated with lower caries experience compared to traditional methods like using glycerine, which was linked to significantly higher odds of caries (OR: 17.7) [38]. One study found that chewing stick users had more caries than toothbrush users among males [35]. However, most studies found no significant association for the use of toothbrushes, chewing sticks, floss, or other specific devices when analyzed independently [18, 36, 42, 51].
- **Other behavioural Variables:** Factors such as the supervisor of brushing, age of onset of cleaning, timing of cleaning [42], and frequency of changing devices [18] were not associated with caries prevalence. One study found that not cleaning after every meal was protective [43].

Risk of Bias Assessment of Included Studies

All but one of the included studies [43] were determined to be of low risk of bias, scoring 7–9 on the quality scale [5, 8, 15, 18, 35-42, 44 - 53] (See Supplemental File 3)

Table 3: Summary of Oral Hygiene Practices and Associations with Dental Caries in Included Studies

Study Author, Year	Oral Hygiene Status (Tool)	Frequency of Mouth Cleaning	Types of Oral Hygiene Devices Used	Caries Prevalence (Assessment Tool)	Key Associations Reported
Sowole et al., 2007 [15]	OHI-S (Good: 78.6%, Fair: 10.5%, Poor: 10.9%)	None: 5%; Once: 71%; Twice: 22.7%; Thrice: 1.3%	Toothbrush (51.4%), Cotton wool (31.4%), Foam (9.6%), Nothing (4.7%), Hand towel (2.9%)	10.6% (dmft)	Higher OHI-S score increased the odds of caries (OR: 2.58).
Okoye et al., 2011 [35]	Not assessed	Occasionally: 7%; Once: 73.1%; Twice: 19.9%	Toothbrush/paste (55.8%), Chewing stick (44.2%)	35.5% (DMFT)	Chewing stick users (males) had more caries (P = 0.004). Fluoridated toothpaste users had less caries (P<0.0001). The frequency of cleaning, types of mouth cleaning devices are not associated with caries prevalence.
Ajayi & Abidemi, 2014 [36]	OHI-S (Good: 66%, Fair: 21.6%, Poor: 12.4%)	Once: 50.2%; Twice: 44.7%; Other: 5.1%	Toothbrush/paste (92.3%), Chewing stick/paste (5%)	10.6% (dmft/DMFT)	No association for frequency, device, or OH status.
Folayan et al., 2015 [37]	OHI-S (Good: 69.6%, Fair: 28.2%, Poor: 2.2%)	< Twice: 81.1%; > Twice: 8.9%	Not specified	6.6% (dmft)	Fair oral hygiene had a lower caries risk (AOR: 0.05).
Olatosi et al., 2015 [38]	Not disclosed	Once: 90.4%; Twice: 9.6%	Toothbrush/paste (51.3%), Salt water/cloth (18.9%), Ground glass (16.2%), Glycerin (13.6%)	21.2% (dmft/dmfs)	Glycerine use increased caries odds vs. fluoridated toothpaste (OR: 17.7).
Olabisi et al., 2015 [8]	OHI-S (Fair: 68.4%, Good: 12.5%, Poor: 19.1%)	Not specified	Not specified	35.1% (DMFT)	Not reported.
Onyejaka and Amobi, 2016 [39]	OHI-S (Fair: 86.2%, Good: 10.5%, Poor: 3.3%)	None: 0.5%; Once: 81.6%; Twice: 16.6%; >Thrice: 1.4%	Not specified	9.8% (dmft)	Poor OH (p > 0.001) was associated with caries.
Akinyamoju et al., 2018 [18]	OHI-S (Fair: 57.7%, Good: 34.8%, Poor: 7.5%)	Occasionally: 7.1%; Once: 74.1%; >Twice: 18.8%	Toothbrush/paste (65.8%), Both^ (24.8%), Chewing stick (6.2%), Other (3.2%)	2.2% (DMFT/dmft)	No association for frequency or device.
Oyedele et al., 2018 [40]	OHI-S (Poor: 44.7%, Fair:	Not specified	Not specified	12.2% (DMFT/dmft)	Fair (AOR: 1.73) and Poor (AOR: 2.13) OH increased caries odds.

Study Author, Year	Oral Hygiene Status (Tool)	Frequency of Mouth Cleaning	Types of Oral Hygiene Devices Used	Caries Prevalence (Assessment Tool)	Key Associations Reported
	38.1%, Good: 17.2%)				
Kolawole et al., 2019 [5]	OHI-S (Fair: 56.2%, Good: 39.4%, Poor: 4.4%)	Not specified	Not specified	14.9% (dmft/DMFT, PUFA/pufa)	Poor OH increased caries odds (OR: 1.83).
Abiola et al., 2019 [41]	OHI-S (Mean scores reported) OHI-S urban:1.20 OHI-S rural: 1.40 OHI-S male: 1.36 OHI-S female: 1.21 OHI-S <3years: 1.26 OHI-S ≥3years: 1.38	Once: 68.3%; Twice: 28.5%; Varies: 3.2%	Cotton wool/paste (45.5%), Toothbrush/paste (33.7%), Cotton wool (29.7%), Glycerine (5.4%)	10.9% (dmft)	Each unit increase in OHI-S score increased caries odds by 64%.
Nwhator et al., 2019 [42]	Not assessed	2-3x/day: 38.2%; >3x/day: 38.6%; Once: 13.2%; Rarely: 9.3%	Water/finger (36.1%), Chewing stick (27.1%), Toothbrush/paste (27.1%)	62.1% (DMFT)	No association for frequency, timing, or device.
Disa et al., 2019 [43]	Not assessed	>Twice: 65.3%; <Twice: 34.7%	Toothbrush/paste (81.5%), Chewing stick (15.3%)	47.6% (DMFT))	Cleaning ≥Twice daily (OR: 0.33) and not after every meal (OR: 0.28) were protective.
Oyapero et al., 2020 [44]	Poor OH: 26.6% (Tool not specified)	Not specified	Not specified	79.1% (dmft/DMFT, PUFA/pufa)	Positive correlation between poor OH and caries severity (ρ=0.307).
Arowolo, 2020 [45]	OHI-S (Fair: 82.7%, Good: 7.7%, Poor: 9.6%)	Twice: 49%; Once: 50.1%; Don't: 1.1%; >Twice: 0.8%	Toothbrush (97.5%), Chewing stick (1.4%)	6.9% (dmft)	Poor OH increased caries odds (AOR: 15.51). Brushing once daily was protective vs. not brushing (AOR: 0.05).
Olatosi et al., 2020 [46]	Not assessed	>Twice: 43.1%; Once: 47.3%; Not daily: 9.6%	Not specified	16.0% (dmft/DMFT)	No association for frequency.
Folayan et al., 2021 [47]	OHI-S (Good: 44.6%, Fair/Poor: 55.4%)	>Twice: 59.7%; <Once: 40.3%	Daily floss use: 11.6%	3.7% (DMFT)	Brushing frequency and floss use are not associated with caries.

Study Author, Year	Oral Hygiene Status (Tool)	Frequency of Mouth Cleaning	Types of Oral Hygiene Devices Used	Caries Prevalence (Assessment Tool)	Key Associations Reported
Onyejaka et al., 2021 [48]	OHI-S (Fair: 85.2%, Good: 12.6%, Poor: 2.6%)	Not specified	Not specified	22.7% (dmft/DMFT)	Good (OR: 0.18) and Fair (OR: 0.58) OH had lower caries odds.
Idowu et al., 2021 [49]	OHI-S (Categories reported)	Once: 68.1%; Twice: 31.9%	Floss use: 10.4%	43.8% (DMFT)	No association between OH status and caries.
Oyedele et al., 2021 [50]	OHI-S (Fair: 41.4%, Good: 34.9%, Poor: 23.7%)	Not specified	Not specified	17.8% (dmft/DMFT)	Poor (OR: 1.79) OH status is significantly associated with caries in suburban areas.
Folayan et al., 2021 [51]	Plaque Index	Once: 91.3%; ≥Twice: 8.7%	Daily floss use: 16.1%	3.4% (DMFT)	No association for brushing frequency, floss use, or OH status.
Oyedele et al., 2022 [52]	OHI-S (Fair: 58.9%, Good: 30.4%, Poor: 10.7%)	Once: 57.6%; Twice: 41.7%; Thrice: 0.5%	Never floss: 48.6%; Rarely floss: 36.9%	10.6% (DMFT)	Poor (OR: 2.5) and Fair (OR: 1.8) OH associated with caries.
Afolabi, 2023 [53]	OHI-S (Fair: 61.4%, Good: 36.4%, Poor: 2.3%)	Once: 94.7%; Twice: 4.9%; >Twice: 0.4%	Toothbrush/paste: 100%	Primary: 13.5%; Permanent: 5% (dmft/DMFT)	No association for OH status or frequency in either dentition.

Note: "Both" refers to the use of both toothbrush/paste and a chewing stick.
Abbreviations: OH: Oral Hygiene; OHI-S: Simplified Oral Hygiene Index; dmft: decayed, missing, filled teeth (primary); DMFT: decayed, missing, filled teeth (permanent); PUFA/pufa: pulpal involvement, ulceration, fistula, abscess index; OR: Odds Ratio; AOR: Adjusted Odds Ratio.

Association between oral hygiene status and dental caries

Pooled data from 10 studies initially suggested poor oral hygiene was associated with lower caries odds (OR 0.52, 95% CI: 0.32–0.85; p=0.01). After removing a study that was an influential outlier [47], the analysis showed a significant 38% decrease in caries prevalence associated with poor oral hygiene (OR 0.62, 95% CI 0.46–0.84; p = 0.006), with moderate heterogeneity (I^2 = 44.6%).

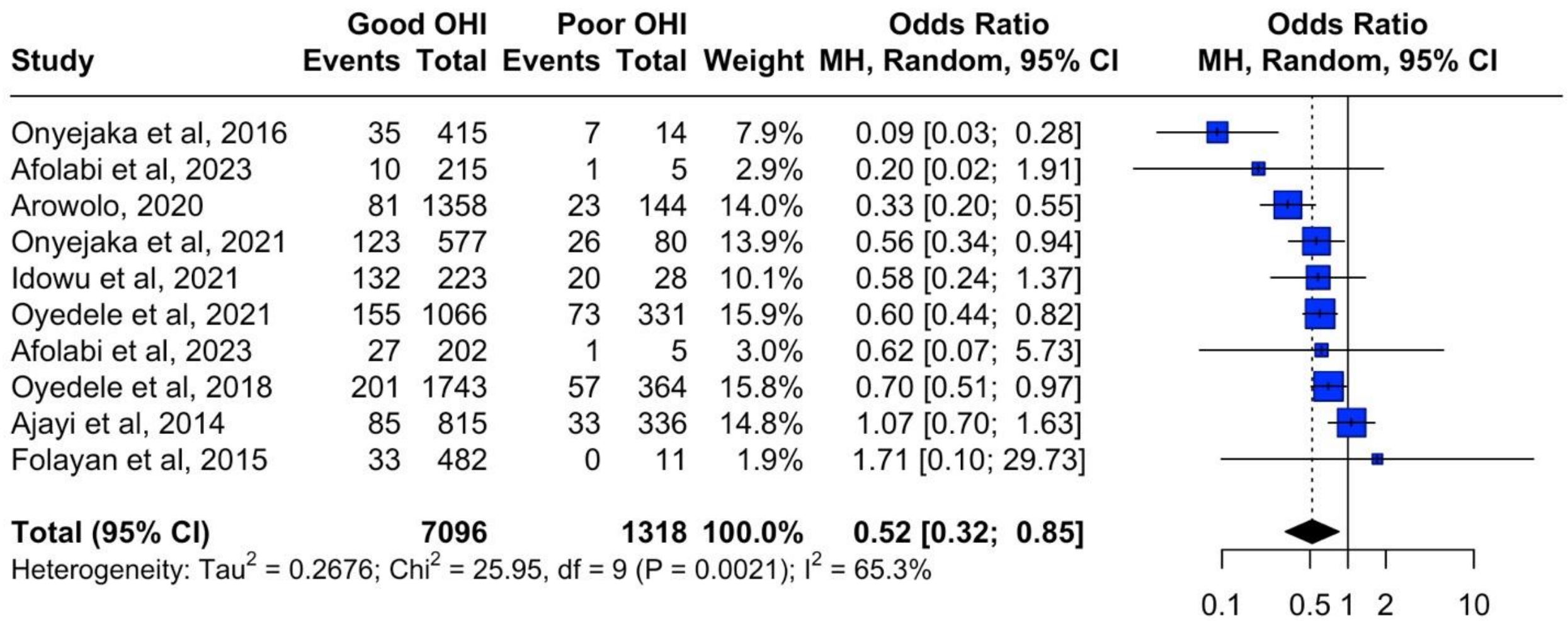

Figure 2: Forest plot showing the meta-analysis of oral hygiene status and caries prevalence

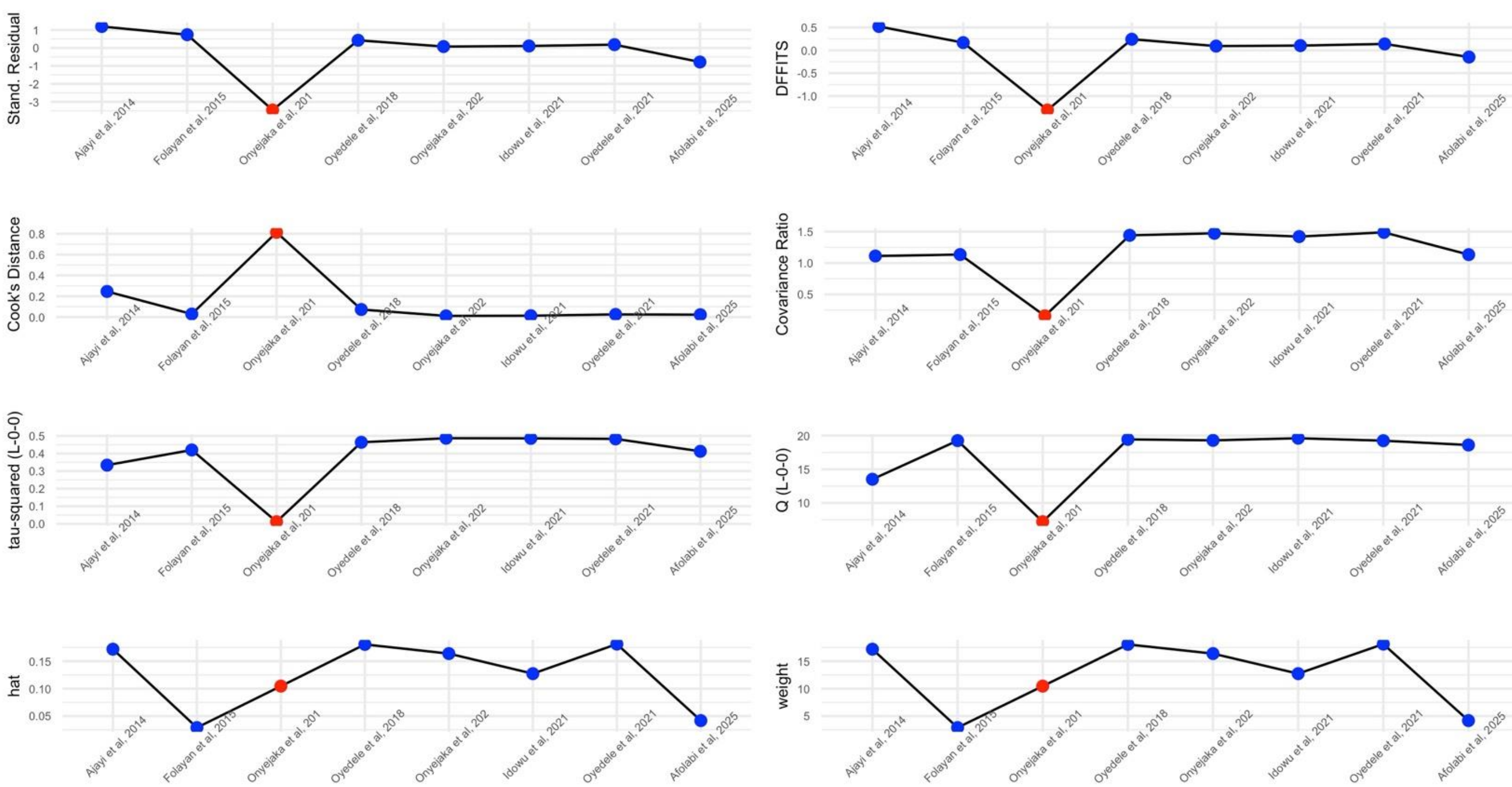

Figure 3: Influential plot showing Onyejaka et al. [47] was an influential study

Subgroup analyses

Subgroup analysis using the type of dentition showed that the type of dentition was a significant moderator in the association of caries prevalence with oral hygiene status (chi^2=7.46, p=0.02). Oral hygiene status was associated with a 64% reduction in odds for caries in the primary dentition and a 49% reduction in caries prevalence in the permanent dentition (Figure 4). Odds reduction observed with good oral hygiene in the mixed dentition was 39% for caries prevalence.

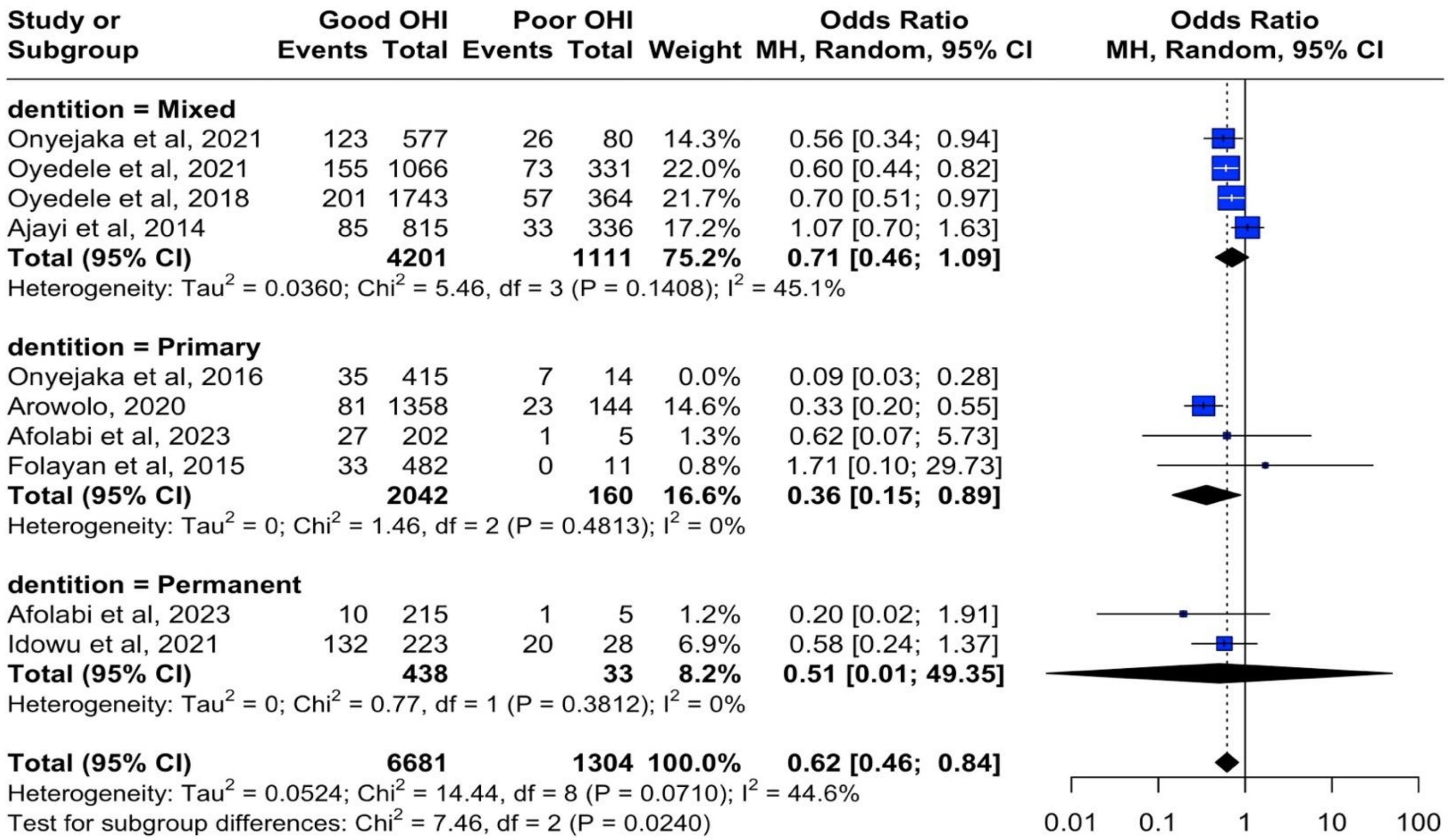

Figure 4: Forest plot showing subgroup analysis by type of dentition for pooled prevalence and OHI

Subgroup analysis with study design also shows nil significant moderating effect of study design (p=0.31). Subgroup analysis across geopolitical zone was not possible as only Southwest zone had sufficient study for a meta-analysis (OR 0.63, 95% CI: 0.41–0.96) as the Southeastern zone and the Northern region only had two and one studies each respectively.

Univariate meta-regression

Univariate meta-regression using the year of publication showed that the year of publication was significant in the association between oral hygiene status and caries with prevalence (p=0.02) Figure 5. R^2 showed that the year of publication accounts for 100% of all the variance in study heterogeneity.

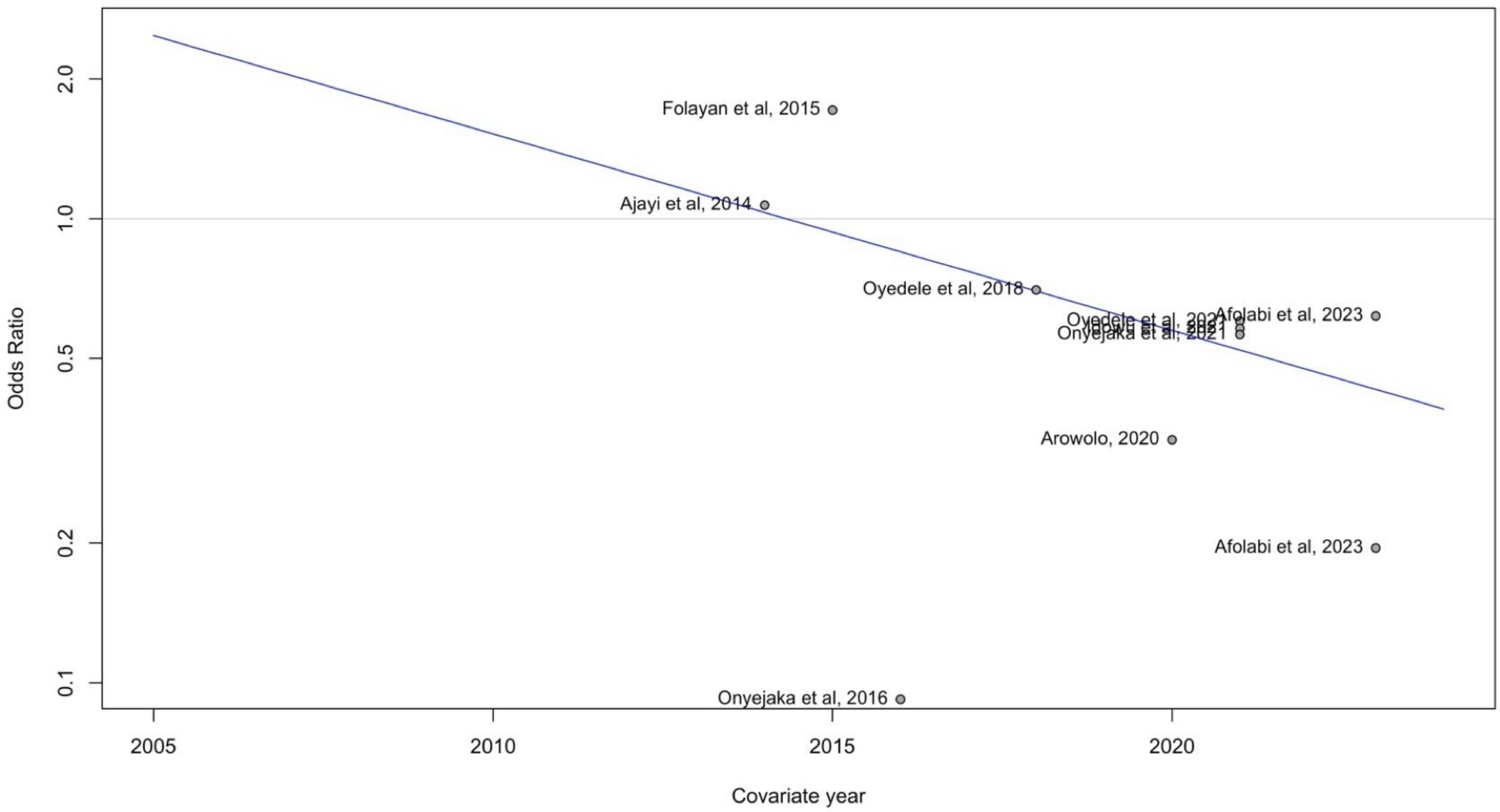

Figure 5: Bubble plot showing the year of publication and caries prevalence

Meta-regression also showed that the individual study sample size (p=0.31) and the mean age of participants (p=0.86) had no significant moderating effect on OH status and caries prevalence in included studies

Publication Bias

Because fewer than 10 included research studies were included, following the removal of the influential study, we could not evaluate the included studies' publication bias, as the lack of statistical power from the inclusion of a few studies would cause [42].

Association between frequency of tooth brushing and dental caries

Analysis of 16 studies showed that brushing teeth at least twice daily was associated with a 99% reduction in the odds of dental caries compared to brushing once daily (OR 0.01, 95% CI 0.00–0.01; $p < 0.001$). Results were consistent across studies ($I^2 = 0\%$).

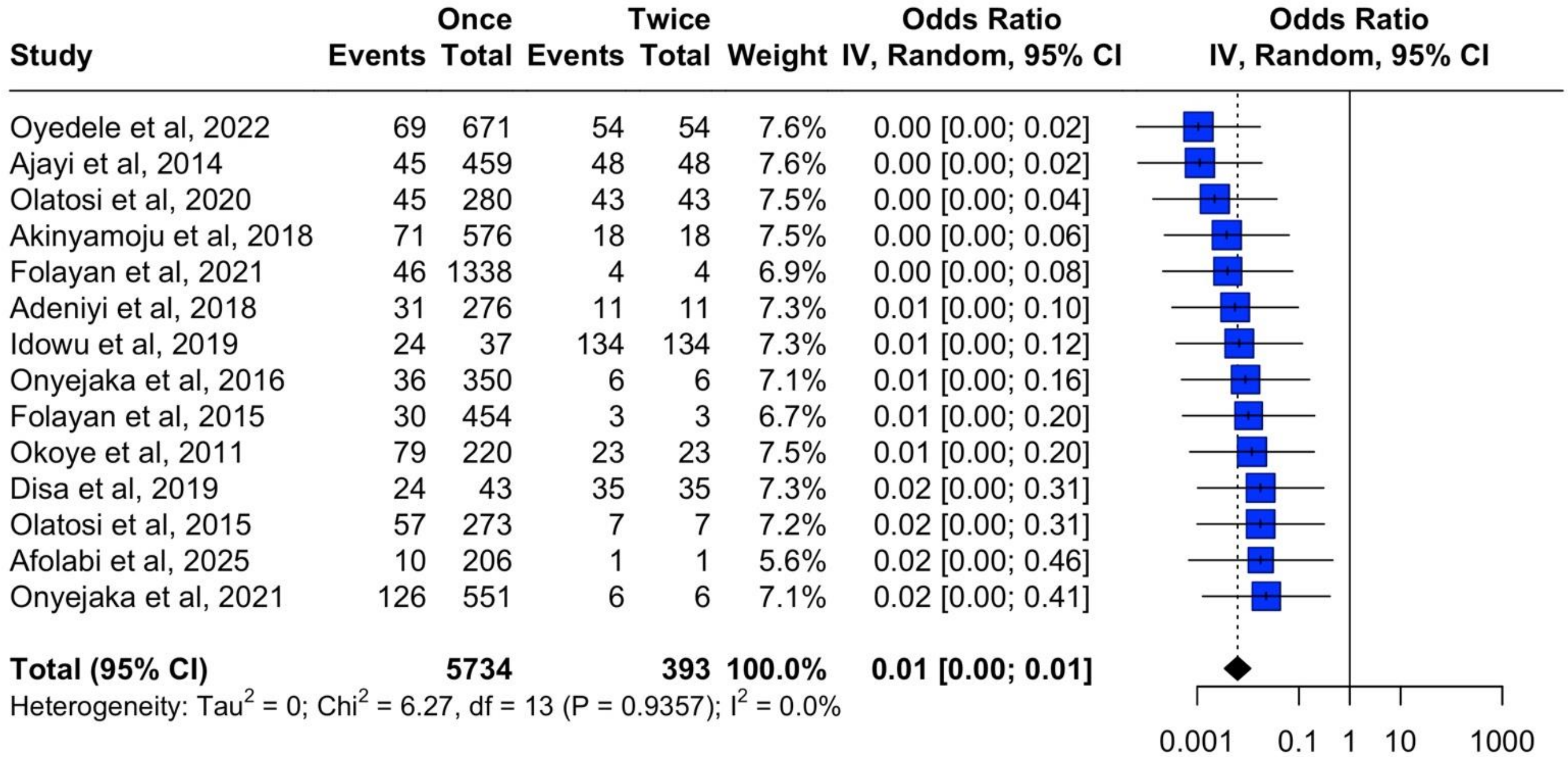

Figure 6: Meta-analysis showing the association of tooth brushing frequency and dental caries

Subgroup analysis

Subgroup analysis shows no significant differences in the pooled odds ratio for tooth cleaning frequency and caries prevalence across geopolitical zones (p = 0.11), type of dentition (p = 0.24) and study design (p = 0.83)

Publication bias

Funnel plot as shown in Figure 7 showed no funnel plot asymmetry to indicate publication bias; objectively, Egger's test also corroborated the absence of publication bias in studies reporting dental caries prevalence and frequency of tooth brushing.

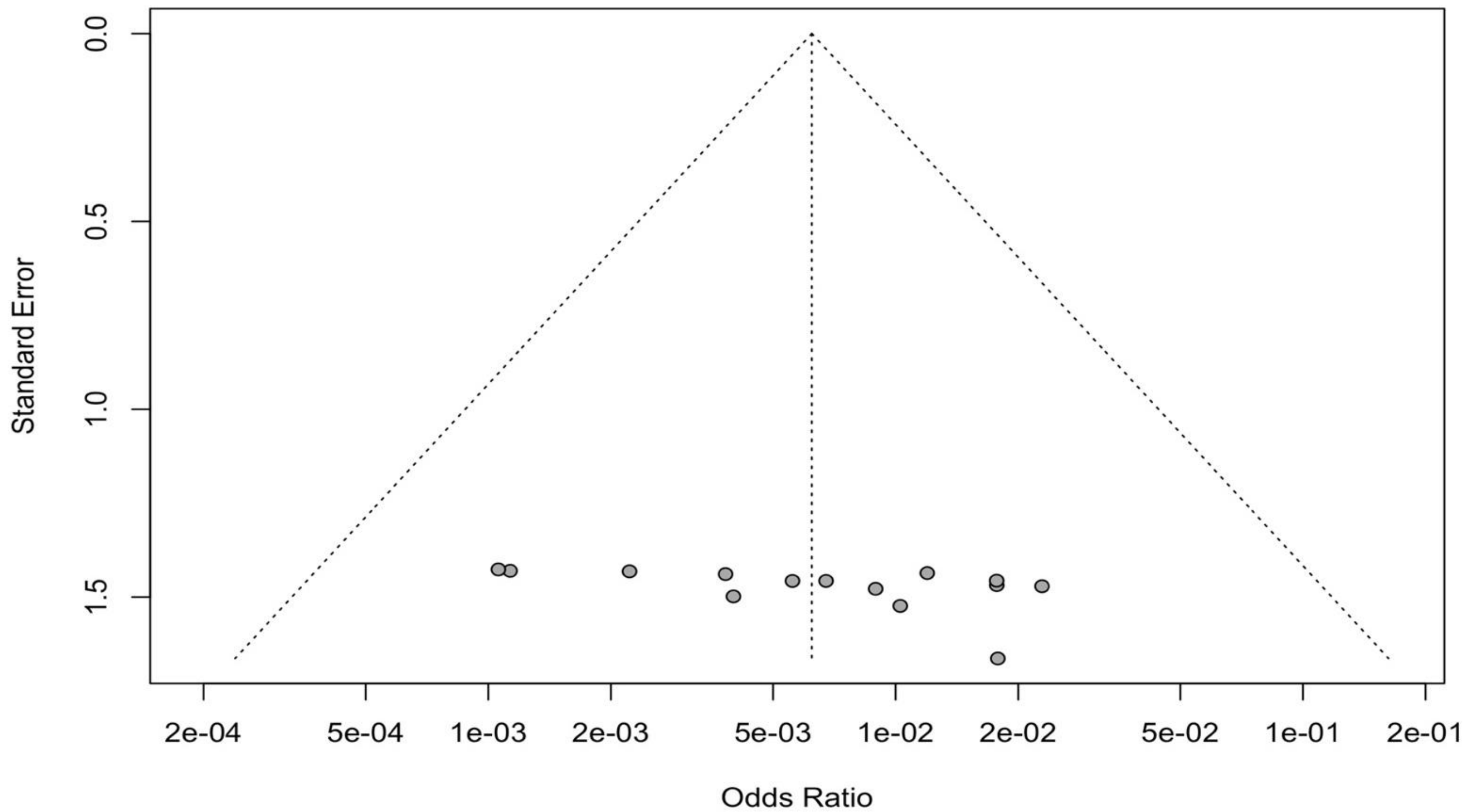

Figure 7: Funnel plot showing plot symmetry and lack of publication bias

Association between tooth cleaning methods and dental caries

Analysis of eight studies found no significant association between the type of tooth cleaning device (e.g., toothbrush) and caries prevalence (OR 1.02, 95% CI 0.52–2.00; p = 0.95). Heterogeneity was high ($I^2 = 79.5\%$). Sensitivity analysis indicated one influential study; its removal yielded a non-significant 19% reduction in caries odds (OR 0.81, 95% CI 0.42–1.54).

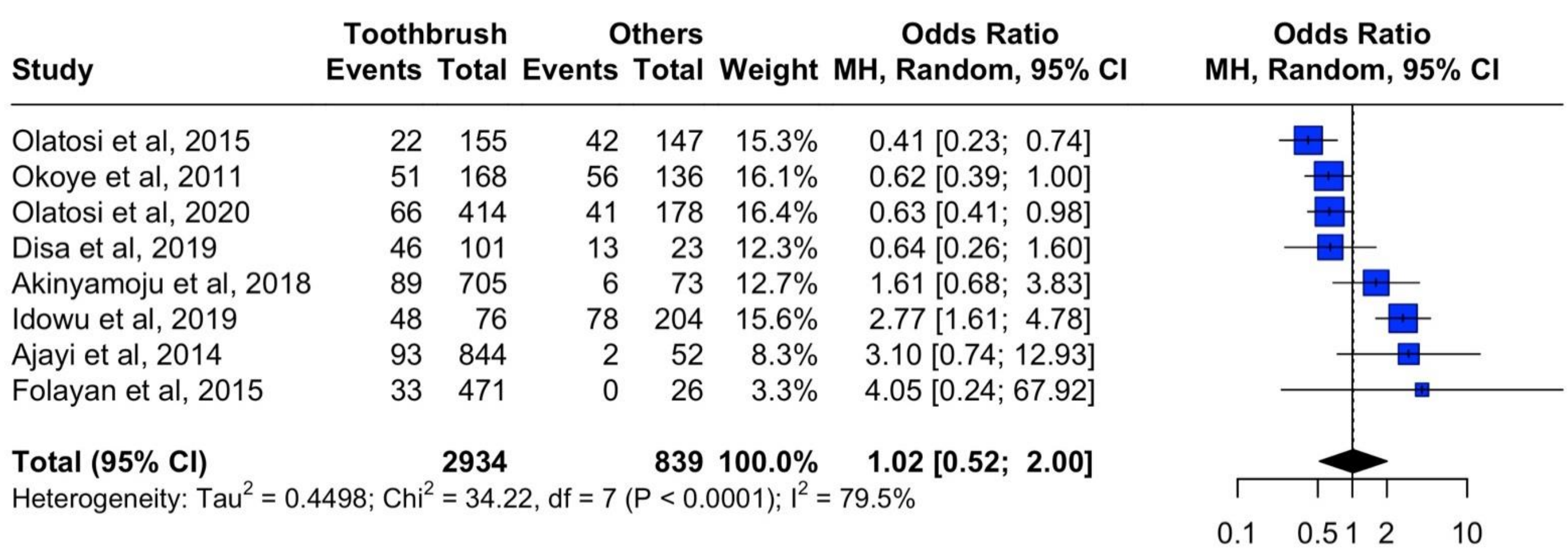

Figure 8: Meta-analysis showing the association of tooth brushing methods and dental caries

Subgroup analyses

Toothbrushing was significantly associated with reduced caries prevalence in primary (42% reduction) and permanent (36% reduction) dentition, but with a 92% increase in mixed dentition (p = 0.0015). The geopolitical zones (p = 0.58) and study design (p = 0.13) exerted no significant moderating effect on the association between pooled prevalence of dental caries and tooth cleaning methods.

Table 4: Subgroup analysis for dentition for Toothbrushing and Dental Caries: Results for subgroups (random effects model)

Dentition	OR	95%-CI	tau^2	tau	Q	I^2
**Permanent**	0.64	[0.26; 1.60]	--	--	--	--
**Mixed**	1.92	[0.05; 75.12]	0	0	0.58	0.0%
**Primary**	0.58	[0.34; 0.99]	0.02	0.15	3.39	11.6%
**Overall**	0.81	[0.42; 1.54]	0.28	0.53		53.1%

Univariate meta-regression using sample size (p=0.043) of individual studies showed that study sample sizes were a significant moderating factor in the association between tooth cleaning methods and caries prevalence (Figure 5). R^2 showed that sample sizes accounted for 79.8% of the study heterogeneity in the included studies.

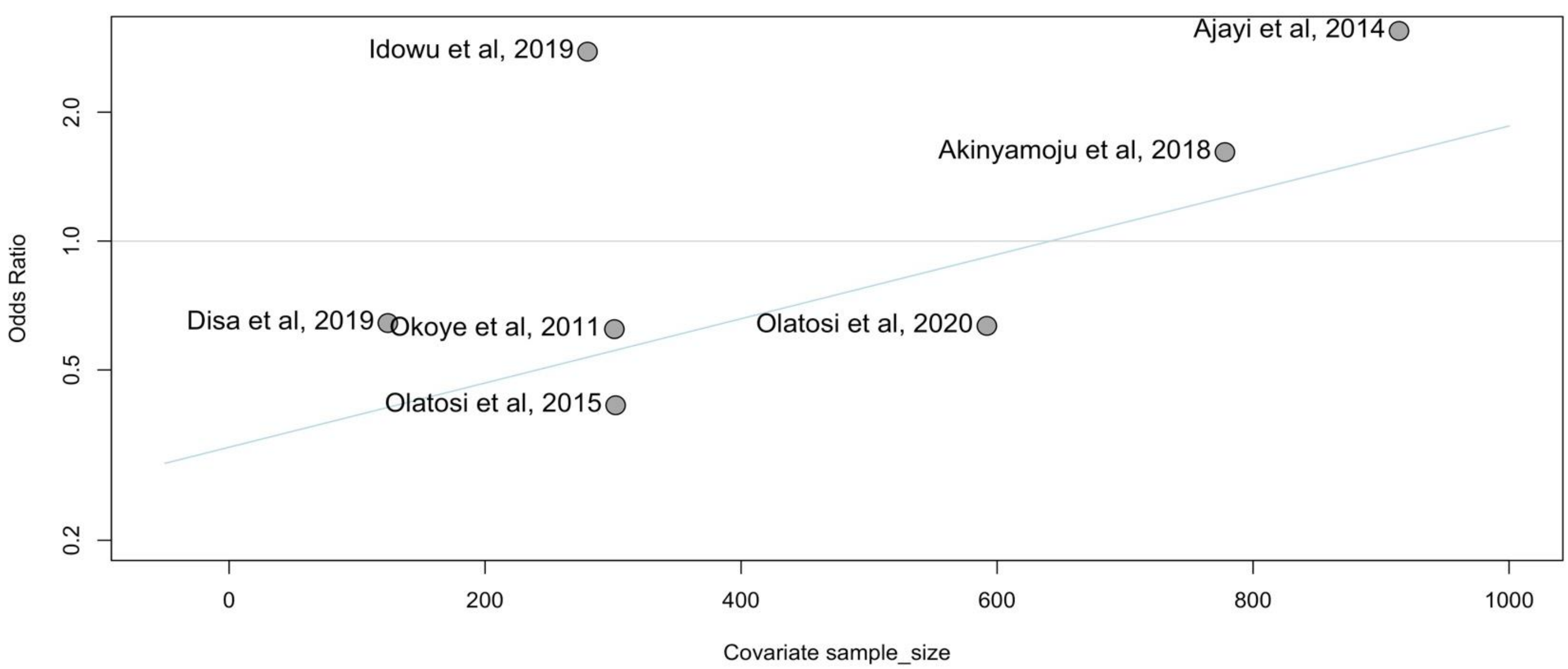

Figure 9: Bubble plot showing the association of tooth brushing methods and sample sizes of studies

The year of publication (p=0.30), and mean age of participants (p=0.88) had no significant moderating effect on caries prevalence and tooth cleaning methods

Discussion

This systematic review and meta-analysis present the first nationally estimated data on the associations between dental caries and oral hygiene practices. The study indicated that oral hygiene status was most commonly measured using the OHI-S, while the prevalence of caries was mainly assessed using the DMFT/dmft indices. However, the metrics for measuring oral hygiene practices were diverse, and indicators lacked uniformity across the included studies. The dental caries experience was higher when oral hygiene was poor, but this relationship was most significant in the primary dentition, and less so in the permanent or mixed dentition. In addition, toothbrushing twice daily or more was associated with a reduction in the odds of having dental caries compared to brushing once daily or less. However, there was no difference in the frequency of dental caries experience among individuals using toothbrushes and toothpaste compared to those using other tooth-cleaning devices.

A strength of the study is the methodological rigor and the transparency of the meta-analytic process, enhancing reproducibility. However, the review has several limitations that should inform the interpretation of findings and guide future research. The cross-sectional design of all included studies precludes causal inference. Unmeasured confounders such as dietary sugar intake, socioeconomic status, fluoride exposure, and access to dental care may influence the observed associations. In addition, there was a significant geographic imbalance, with most studies conducted in Southern Nigeria, particularly the Southwest. This limits the generalizability of findings to Northern Nigeria, where cultural, dietary, and socio-economic contexts may differ substantially. Future studies must prioritize this region to enable nationally representative conclusions. Despite these limitations, the study provides important information.

The first insightful finding from this systematic review and meta-analysis is the direct correlation between the prevalence of dental caries and twice-daily brushing. This contradicts simplistic biological models but becomes intelligible when considering the differential exposure to dietary sugars across socioeconomic groups, a key component of the broader caries etiology. In Nigeria, a low-middle-income country, a distinct socio-economic gradient influences oral health risk profiles: urban and higher-income populations are experiencing a nutrition transition, characterized by increased consumption of processed foods and sugary snacks and beverages [54] as seen in other transiting economies [55-59]. While these groups typically have better access to toothbrushes and toothpaste and better dental service utilisation (leading to better OHI-S scores), their high-frequency exposure to fermentable carbohydrates creates a persistent cariogenic challenge [60, 61, 62]. Here, even fair oral hygiene may be insufficient to counteract a high-sugar diet, leading to caries despite relatively better plaque scores.

On the other hand, rural and low-income populations may have more traditional, less processed diets with lower between-meal sugar intake [63]. However, they face barriers to accessing modern oral hygiene tools and preventive dental care [64]. Their oral hygiene status may be classified as poor due to plaque accumulation from less frequent or less effective cleaning methods. Yet, the lower dietary sugar exposure results in a less cariogenic oral environment, potentially leading to the observed lower caries odds. This aligns with the substrate limitation principle in cariology. Thus, the OHI-S index primarily captures plaque accumulation but not the diet's cariogenic potential. In a setting like Nigeria with stark socioeconomic disparities, this creates an ecological fallacy: the group with better hygiene (a proxy for higher SES and urbanization) carries a higher diet-mediated risk, while the group with poorer hygiene (a proxy for lower SES and more traditional living) has a lower diet-mediated risk. This finding needs to be studied further as the trend seems to be changing among adolescents in Nigeria, wherein poorer oral hygiene is associated with higher dental caries prevalence [65].

The significant association between poor oral hygiene, dental caries, and the primary dentition also needs to be explored further, as it is currently under-explored in its full complexity because of the lifelong consequences. Primary teeth are structurally more vulnerable to rapid caries progression than permanent teeth due to key anatomical differences. The broad contact points between molars trap food and plaque allow for early caries formation. Their enamel and dentine are thinner and less mineralized, enabling faster penetration by caries [66]. While most studies focus on S. *mutans* and caries aetiology, the broader plaque microbiome and its ecological shifts (dysbiosis) in response to hygiene and diet in early childhood are an evolving area of study [67]. Further exploration must move beyond reaffirming the link and focus on developing and implementing culturally sensitive, equitable, and evidence-based strategies that disrupt this association at the individual, family, and community levels. This is an investment in a child's immediate well-being and their long-term oral and general health trajectory.

The second insight from this systematic review and meta-analysis is the affirmation that twice-daily brushing is strongly associated with a reduced prevalence of dental caries in the study population, thereby highlighting the protective advantages of the frequency of tooth brushing in mitigating dental caries. Brushing teeth twice daily enhances plaque control by consistently disrupting the oral biofilm [68, 69]. This aligns with the conclusions made in the literature on the importance of regular brushing as a preventive measure against dental caries [68, 70]. The findings have important implications for practice, policy, and research. National oral health campaigns and school-based programs should reinforce messaging on twice-daily toothbrushing using methods identified for effectively communicating oral health [71].

The efficacy of twice-daily tooth brushing can be further enhanced by considering the optimal timing of brushing, either before or after meals, to eliminate food debris and minimize the impact of sucrose exposure through prompt cleaning after eating. [72]. In addition, using dental floss for interdental plaque removal and bacteriostatic mouthwashes can augment the effectiveness of brushing by chemically disrupting the oral biofilm. [73, 74, 75]. However, none of the studies included in this study reinforced the value of using adjuncts in reducing the caries experience, although none explored the relationship between the use of mouthwash and dental caries experience, thereby creating a knowledge gap. This gap can be bridged through future studies.

Also, although prior studies had indicated that the complementary use of fluoride-containing toothpaste with toothbrushing can further enhance the efficacy of twice-daily tooth brushing in caries control [76, 77], and local studies in the country had reinforced this [35, 78], the current study did not find such an association. This result should be interpreted cautiously and not as a contradiction of established evidence; rather, it may reflect contextual specificities or methodological limitations. The widespread use of fluoridated toothpaste in Nigeria, with over 90% of the population using it daily [67], likely created a ceiling effect in the primary studies, with such homogeneous exposure preventing the detection of a differential effect between comparison groups. These findings suggest that in a high-fluoride-toothpaste-use setting, the additional benefit of fluoride toothpaste may be obscured, and that other co-factors, such as dietary habits, may become more salient modifiers of caries risk [79].

A culturally significant finding of this meta-analysis is that toothbrushing with toothpaste was not superior to other devices in reducing the risk for caries. A population tooth cleaning tool in Nigeria is the chewing stick, drawn from plants such as *Salvadora persica* and Neem [80]. It holds deep cultural and religious significance and is traditionally believed to possess cleansing and medicinal properties [81]. The affordability and widespread availability of chewing sticks make them a sustainable alternative to toothbrushes in a resource-limited context. This study finding underscores that effective oral hygiene does not require expensive tools but rather relies on technique and consistency. A well-used chewing stick may clean more effectively than a poorly used toothbrush. This finding raises an important hypothesis that needs to be tested in future studies, challenging the longstanding assumption that modern tools are inherently superior and shifting the discussion toward a more evidence-based and culturally competent understanding of oral hygiene practices in Nigeria.

For clinicians, this finding may necessitate a shift in counselling. Rather than dismissing traditional practices, oral health professionals should engage patients with respect, asking what they use, how often, and demonstrate effective techniques. The counselling message should emphasize that the goal is efficient plaque removal,

achievable with both toothbrushes and other tooth cleaning tools, while reinforcing the importance of brushing twice daily. For patients reliant on other tooth cleaning tools like chewing sticks, clinicians can encourage proper preparation and use, while complementing them with fluoride exposure through alternatives such as fluoridated mouthwash, where feasible [82]. At the community and public health level, the study evidence supports the need for campaigns that empower rather than prescribe. Messages can build on existing practices rather than attempting to replace them. This culturally sensitive approach acknowledges traditional methods while amplifying the most important public health message about frequency and thoroughness rather than the tool itself, which resonates with cultural realities and socioeconomic conditions. Further studies on using culturally appropriate oral hygiene practices are warranted to enhance our understanding of effective strategies for caries prevention in diverse populations.

In addition, the diversity observed in the array of measures employed to assess oral hygiene practices in the studies included in this systematic review may have contributed to the substantial outcome heterogeneity. It is, however, essential to study these measures to develop context-specific and relevant instruments for caries risk assessment that enable laypersons and healthcare practitioners to screen individuals at risk for caries. This gap highlights the need to develop standardized, validated tools for oral hygiene assessment in future research conducted in Nigeria.

On the other hand, we observed that the studies included in this systematic review and meta-analysis assessed dental caries using the DMFT/dmft indices. [83], a reliable and user-friendly tool for capturing dental caries experiences, with some limitations to its use [84, 85], one of which is its inability to detect enamel caries (caries in their reversible form) [86, 87]. Thus, the prevalence of caries would likely be underestimated in the studies included in this meta-analysis. Future research endeavors in Nigeria should consider transitioning to tools capable of identifying enamel caries, such as ICDAS, Cardiogram, and CAMBRIA, among others. [88].

In conclusion, this study offers a nuanced understanding of oral hygiene practices and their association with the prevalence of dental caries in Nigeria. This review synthesizes a growing body of Nigerian research to provide evidence that is both globally coherent and locally nuanced. It affirms that twice-daily brushing and good oral hygiene status are significantly associated with lower caries prevalence. It also provides data suggesting that, within the Nigerian context, the efficacy of oral hygiene may depend more on the frequency and quality of the practice than on a specific cleaning device. These findings advocate for public health strategies that promote behavioral consistency and effective techniques, while being respectful of cultural practices, to effectively reduce the burden of dental caries in Nigeria.

Acknowledgments

The authors thank the Nigerian Institute for Medical Research for supporting the study.

Data Sharing

All datasets generated and analyzed, including the study protocol, search strategy, list of included and excluded studies, data extracted, analysis plans, and quality assessment, are available in the article and upon request from the corresponding author.

Funding

Grant Number: 5NM-ADJGT-22-0082 of $130.00 from the Nigerian Institute for Medical Research.

Conflict of Interest

The authors declared no conflict of interest.

Declaration of Competing Interest

The authors declare that they have no known competing financial interests or personal relationships that could have appeared to influence the work reported in this paper.

Authors Contributions

MOF developed the idea. TG, AS, IA, MO, OA, MOF, GUE, and FTA wrote and registered the protocol on PROSPERO. UN, AOE, JL, GUE, and FTA undertook the literature searches. AA, AMA, and CO screened titles, abstracts, and full-text articles, extracted all data, and performed the initial data analysis. AA and FTA undertook a meta-analysis. MET, OCE, MOF, GUE, AA, and FTA wrote the first draft of the manuscript. All authors contributed to the writing of each manuscript draft and have approved the final submitted version.

Ethical approval

Ethical approval was not required for this systematic review as the research was based on information retrieved from published studies.

References

1. Akpata ES. Oral health in Nigeria. Int Dent J. 2004;54:361-366.
2. Nyvad B. The role of oral hygiene. In: Fejerskov O, Nyvad B, Kidd E, eds. Dental Caries: The Disease and Its Clinical Management. 3rd ed. Wiley Blackwell; 2015:171-176.

3. Sampson V. Oral hygiene risk factor. Br Dent J. 2020;228(8):569. doi:10.1038/s41415-020-1545-3
4. Adeniyi AA, Sofola OO, Kalliecharan RV. An appraisal of the oral health care system in Nigeria. Int Dent J. 2012;62(6):292-300.
5. Kolawole KA, Folayan MO. Association between malocclusion, caries, and oral hygiene in children 6 to 12 years old resident in suburban Nigeria. BMC Oral Health. 2019;19(1):66.
6. Stein C, Santos NML, Hilgert JB, Hugo FN. Effectiveness of oral health education on oral hygiene and dental caries in schoolchildren: systematic review and meta-analysis. Community Dent Oral Epidemiol. 2018;46(1):30-37.
7. Hujoel PP, Hujoel MLA, Kotsakis GA. Personal oral hygiene and dental caries: a systematic review of randomized controlled trials. Gerodontology. 2018;35(4):282-289.
8. Olabisi AA, Udo UA, Ehimen UG, et al. Prevalence of dental caries and oral hygiene status of a screened population in Port Harcourt, Rivers State, Nigeria. J Int Soc Prev Community Dent. 2015;5(1):59-63.
9. Quadri MFA, Shubayr MA, Hattan AH, Wafi SA, Jafer AH. Oral hygiene practices among Saudi Arabian children and its relation to their dental caries status. Int J Dent. 2018;2018:1-7.
10. Folayan MO, Khami MR, Folaranmi N, et al. Determinants of preventive oral health behaviour among senior dental students in Nigeria. BMC Oral Health. 2013;13:28.
11. Uguru N, Onwujekwe O, Ogu UU, Uguru C. Access to oral health care: a focus on dental caries treatment provision in Enugu, Nigeria. BMC Oral Health. 2020;20(1):121.
12. World Health Organization. Oral health – fact sheets. 2019. Accessed August 25, 2025. https://www.who.int/news-room/fact-sheets/detail/oral-health
13. Feldens CA, Kramer PF, Vargas-Ferreira F. The role of diet and oral hygiene in dental caries. In: Pediatric Restorative Dentistry. Springer; 2019:31-55.
14. Folayan MO, Chukwumah NM, Onyejaka N, Adeniyi AA, Olatosi OO. Appraisal of the national response to the caries epidemic in children in Nigeria. BMC Oral Health. 2014;14:76.
15. Sowole A, Sote E, Folayan MO. Dental caries pattern and predisposing oral hygiene-related factors in Nigerian preschool children. Eur Arch Paediatr Dent. 2007;8:206-210.
16. Okoye L, Ekwueme O. Prevalence of dental caries in a Nigerian rural community: a preliminary local survey. Ann Med Health Sci Res. 2011;1(2):187-196.
17. Sofola OO. Implications of low oral health awareness in Nigeria. Niger Med J. 2010;51(3):131-133.
18. Akinyamoju CA, Dairo DM, Adeoye IA, Akinyamoju AO. Dental caries and oral hygiene status: survey of schoolchildren in rural communities, Southwest Nigeria. Niger Postgrad Med J. 2018;25(4):239-245.
19. Okeigbemen SA. The prevalence of dental caries among 12 to 15-year-old school children in Nigeria: report of a local survey and campaign. Oral Health Prev Dent. 2004;2(1):27-31.

20. Wen PYF, Chen MX, Zhong YJ, Dong QQ, Wong HM. Global burden and inequality of dental caries, 1990 to 2019. J Dent Res. 2022;101(4):392-399.
21. Olusile AO, Adeniyi AA, Orebanjo O. Self-rated oral health status, oral health service utilization, and oral hygiene practices among adult Nigerians. BMC Oral Health. 2014;14:140. doi:10.1186/1472-6831-14-140
22. World Dental Federation. No Health Without Oral Health: How the Dental Community Can Leverage the NCD Agenda to Deliver on the 2030 Sustainable Development Goals. Proceedings of the FDI–NCD Alliance Joint Session; 2017. Accessed August 25, 2025. https://www.fdiworlddental.org/sites/default/files/2020-11/fdi-wdc2017_ncd_session_proceedings.pdf
23. Page MJ, McKenzie JE, Bossuyt PM, et al. The PRISMA 2020 statement: an updated guideline for reporting systematic reviews. Int J Surg. 2021;88:105906. doi:10.1016/j.ijsu.2021.105906
24. Brown D. A review of the PubMed PICO tool: using evidence-based practice in health education. Health Promot Pract. 2020;21(4):496-498.
25. Munn Z, Tufanaru C, Aromataris E. The development of software to support multiple systematic review types: the Joanna Briggs Institute System for the Unified Management, Assessment and Review of Information (JBI SUMARI). JBI Evid Implement. 2019;17(1):36-43.
26. Chandler J, Cumpston M, Li T, Page MJ, Welch V, eds. Cochrane Handbook for Systematic Reviews of Interventions. 2nd ed. Wiley; 2019. doi:10.1002/14651858
27. Atkins D, Best D, Briss PA, et al. Systems for grading the quality of evidence and the strength of recommendations I: critical appraisal of existing approaches. BMC Health Serv Res. 2004;4:38.
28. Higgins, J. P. T., & Thompson, S. G. (2002). Quantifying heterogeneity in a meta-analysis. *Statistics in Medicine*, 21(11), 1539-1558.
29. Huedo-Medina, T. B., Sánchez-Meca, J., Marín-Martínez, F., & Botella, J. (2006). Assessing heterogeneity in meta-analysis: Q statistic or I^2 index?. *Psychological Methods*, 11(2), 193–206.
30. Chandler J, Cumpston M, Li T, Page MJ, Welch V, eds. Cochrane Handbook for Systematic Reviews of Interventions. 2nd ed. Wiley; 2019. doi:10.1002/14651858
31. Higgins JPT, Altman DG, Sterne JAC. Assessing risk of bias in included studies. In: Higgins JPT, Green S, eds. Cochrane Handbook for Systematic Reviews of Interventions. Version 5.0.0. The Cochrane Collaboration; 2011.
32. Higgins JPT, Thompson SG, Deeks JJ, Altman DG. Measuring inconsistency in meta-analyses. BMJ. 2003;327(7414):557-560.
33. Hoy D, Brooks P, Woolf A, et al. Assessing risk of bias in prevalence studies: modification of an existing tool and evidence of interrater agreement. J Clin Epidemiol. 2012;65(9):934-939. doi:10.1016/j.jclinepi.2011.11.014

34. Atkins D, Best D, Briss PA, et al. Systems for grading the quality of evidence and the strength of recommendations I: critical appraisal of existing approaches. BMC Health Serv Res. 2004;4:38.
35. Okoye L, Ekwueme O. Prevalence of dental caries in a Nigerian rural community: a preliminary local survey. Ann Med Health Sci Res. 2011 Jul;1(2):187-95. PMID: 23209974; PMCID: PMC3507107.
36. Ajayi DM, Abiodun-Solanke IF. Sociobehavioural risk factors of dental caries among selected adolescents in Ibadan, Nigeria. *Pediatric Dental Journal*. 2014;24(1):33-38.
37. Folayan MO, Kolawole KA, Oziegbe EO, et al. Prevalence and early childhood caries risk indicators in preschool children in suburban Nigeria. BMC Oral Health. 2015;15(1):72. doi:10.1186/s12903-015-0069-4
38. Olatosi O, Inem V, Sofola O, Prakash P, Sote E. The prevalence of early childhood caries and its associated risk factors among preschool children referred to a tertiary care institution. Niger J Clin Pract. 2015;18(4):493-501.
39. Onyejaka NK, Amobi EO. Risk factors of early childhood caries among children in Enugu, Nigeria. Braz Res Pediatr Dent Integr Clin. 2016;16(1):381-391. doi:10.4034/PBOCI.2016.161.40
40. Oyedele TA, Fadeju AD, Adeyemo YI, Nzomiwu CL, Ladeji AM. Impact of oral hygiene and socio-demographic factors on dental caries in a suburban population in Nigeria. Eur Arch Paediatr Dent. 2018 Jun;19(3):155-161. doi: 10.1007/s40368-018-0342-z.
41. Abiola AA, Eyitope OO, Sonny OJ, Oyinkan OS. Dental caries occurrence and associated oral hygiene practices among rural and urban Nigerian preschool children. J Dent Oral Hyg. 2009;1(5):64-70.
42. Nwhator SO, Idowu EA, Afolabi AO. Oral Hygiene Status, Practices, and Awareness of Medium Security Prison Inmates in Northeastern Nigeria. EC Dental Science 18.3 (2019): 491-501.
43. Disa HA, Balami AB, Mala AK, Fusami MA, Gimba UM. Predictors of dental caries among adults and adolescents in a dental clinic in north-eastern Nigeria. Int J Public Health Clin Sci. 2019;6(5):123-?. https://doi.org/10.32827/ijphcs.6.5.123
44. Oyapero A, Adenaike A, Edomwonyi A, Adeniyi A, Olatosi O. Association between dental caries, odontogenic infections, oral hygiene status, and anthropometric measurements of children in Lagos, Nigeria. Braz J Oral Sci. 2020;19:e201431.
45. Arowolo OTE. Determination Of the Association Between Nutritional Status and Dental Caries In 6-16-Year-Old School Children in Ile – Ife. A Dissertation Submitted to the Faculty of Dental Surgery, West African College of Surgeons, in Partial Fulfillment Of The Requirements for the Award of Fellowship of the College in Paediatric Dentistry. October 2020
46. Olatosi OO, Oyapero A, Onyejaka NK. (2020). Disparities in caries experience and socio-behavioural risk indicators among private school children in Lagos, Nigeria.Pesquisa Brasileira Em Odontopediatria E Clínica Integrada, 20:e0023. https://doi.org/10.1590/pboci.2020.102

47. Folayan MO, Oginni O, Adekoya-Sofowora CA, et al. Associations between a history of sexual abuse and dental anxiety, caries experience, and oral hygiene status among adolescents in suburban South West Nigeria. BMC Oral Health. 2021;21(1):229. doi:10.1186/s12903-021-01587-7
48. Onyejaka NK, Olatosi OO, Ndukwe NA, Amobi EO, Okoye LO, Nwamba NP. Prevalence and Associated Factors of Dental Caries among Primary School Children in South-East Nigeria. Niger J Clin Pract. 2021 Sep;24(9):1300-1306. doi: 10.4103/njcp.njcp_633_20.
49. Idowu AE, Fakuade BO, Taiwo OO, Majekodunmi JO, Alufohai OO, Sandabe FK. Dental caries prevalence, restorative needs, and oral hygiene status in the adult population: a cross-sectional study among nurses in Jos University Teaching Hospital, Jos, Nigeria. *Niger J Basic Clin Sci*. 2021;18(1):35-41. doi:10.4103/njbcs.njbcs_32_20.
50. Oyedele T, Fadeju A, Adeyemo Y, Nzomiwu C, Ladeji A. Impact of oral hygiene and socio-demographic factors on dental caries in a suburban population in Nigeria. Eur Arch Paediatr Dent. 2018;19(3):155-161.
51. Folayan MO, El Tantawi M, Chukwumah NM, Alade M, Oginni O, Mapayi B, Arowolo O, Sam-Agudu NA. Individual and familial factors associated with caries and gingivitis among adolescents resident in a semi-urban community in South-Western Nigeria. BMC Oral Health. 2021 Mar 27;21(1):166. doi: 10.1186/s12903-021-01527-x.
52. Oyedele TA, Jegede AT, Adeyemo YI, Ladeji AM, Adetayo AM, Oladapo O. Associations between dental caries, oral hygiene status, and oral health practices of first-year undergraduates in a private university in Nigeria. J Paediatr Dent Res Pract. 2022;3(1-2):22-33.
53. Afolabi JO. Comparative assessment of the oral health status of children aged 4-16 years with and without sickle cell anaemia resident in Ile-Ife and Ilesha, Osun State, Nigeria. A dissertation submitted to the Faculty of Dental Surgery, West African College of Surgeons, in partial fulfilment of the requirements for the award of Fellowship of the College in Paediatric Dentistry. April 2023
54. Petrikova I, Bhattacharjee R, Fraser PD. The ‘Nigerian Diet’ and Its Evolution: Review of the Existing Literature and Household Survey Data. *Foods*. 2023; 12(3):443. https://doi.org/10.3390/foods12030443
55. Popkin BM, Adair LS, Ng SW. Global nutrition transition and the pandemic of obesity in developing countries. Nutr Rev. 2012 Jan;70(1):3-21. doi: 10.1111/j.1753-4887.2011.00456.x.
56. Kapoor V. Dental Caries and the modern world. Indian J Dent. 2014 Oct;5(4):171. doi: 10.4103/0975-962X.144716.
57. Ronto, R.; Wu, J.H.; Singh, G.M. The global nutrition transition: Trends, disease burdens and policy interventions. Public Health Nutr. 2018, 21, 2267–2270. =
58. Popkin, B.M. Global nutrition dynamics: The world is shifting rapidly toward a diet linked with noncommunicable diseases. Am. J. Clin. Nutr. 2006, 84, 289–298.

59. Hawkes, C. Uneven dietary development: Linking the policies and processes of globalization with the nutrition transition, obesity and diet-related chronic diseases. Glob. Health 2006, 2, 4.
60. Egbunah UP, Sofola OO, Uti OG. The Role of Socioeconomic Class on Oral Healthcare Practices and Oral Health Status of Secondary School Students in Lagos, Nigeria. Nig J Dent Res 2023; 8(1):30-39. https://dx.doi.org/10.4314/njdr.v8i1.6
61. Oyedele TA, Folayan MO, Chukwumah NM, Onyejaka NK. Social predictors of oral hygiene status in school children from suburban Nigeria. Braz Oral Res. 2019 Jul 1;33:e022. doi: 10.1590/1807-3107bor-2019.vol33.0022. Erratum in: Braz Oral Res. 2019 Sep 09;33:e022err. doi: 10.1590/1807-3107bor-2019.vol33.0022erratum. PMID: 31269111.
62. Foláyan MO, Zuñiga RAA, Mohebbi SZ and Khami MR (2025) Association between early childhood caries and parental educational status among children in Ile-Ife, Nigeria. Front. Oral Health 6:1581589. doi: 10.3389/froh.2025.1581589
63. Dolislager M, Liverpool-Tasie LSO Mason NM, Reardon T, Tschirley D. 2022. Consumption of healthy and unhealthy foods by the African poor: Evidence from Nigeria, Tanzania, and Uganda. Agricultural Economics, International Association of Agricultural Economists, vol. 53(6), pages 870-894. DOI: 10.1111/agec.12738
64. Folorunsho S, Ajayi V, Sanmori M, Suleiman M, Abdullateef R, Abdulganiyu A. Access to and utilization of dental care services by older adults in Nigeria: barriers and facilitators. Special Care Dent. 2025. doi:10.1111/scd.70040
65. Afolabi AO, Ehizele AO, Nnawuihe UC, Nwaokorie FO, Nwaozuru U, Owotade F, Lusher J, Eleje GU, Alade OT, Akinsolu FT, Ezechi OC, Foláyan MO. Dental caries prevalence, preventive behaviour and related factors among adolescents in Nigeria: a 10-year systematic review and meta-analysis. BMC Oral Health. 2025 Jul 10;25(1):1138. doi: 10.1186/s12903-025-06504-2.
66. Acs G, Shulman R, Ng MW, Chussid S. The effect of dental rehabilitation on the body weight of children with early childhood caries. Pediatr Dent. 1999 Mar-Apr;21(2):109-13. PMID: 10197335.
67. Rosier B. T., Marsh P. D., Mira A. (2018). Resilience of the oral microbiota in health: mechanisms that prevent dysbiosis. J. Dent. Res. 97, 371–380. doi: 10.1177/0022034517742139
68. Attin T, Hornecker E. Tooth brushing and oral health: how frequently and when should tooth brushing be performed? Oral Health Prev Dent. 2005;3(3):135-40. PMID: 16355646.
69. Hayasakia H, Saitoh I, Nakakura-Ohshima K, Hanasaki M, Nogami Y, Nakajima T, et al. Tooth brushing for oral prophylaxis. *Jpn Dent Sci Rev*. 2014. doi:10.1016/j.jdsr.2014.04.001
70. Iba, B. & Adamu, V. E. (2021). Tooth brushing: An effective oral hygiene measure. Orapuh Journal, 2(2), e811.

71. Chukwuemeka AN, Oveh R, Dabar A, Afolabi O, Ibraheem SA, Akinsolu FT, Salako AO, Alade OT, Owotade F, Eleje GU, Ezechi O, Lusher J, Foláyan MO. A scoping review of school-based oral health interventions among adolescents in Nigeria. Front Oral Health. 2025 Jul 29;6:1577753. doi: 10.3389/froh.2025.1577753.
72. Sulastri, S., ., S., & Sulistyani, H. (2022). The effect of tooth brushing before and after eating on salivary pH of elementary school students. *International Journal Of Community Medicine And Public Health*, *9*(3), 1237–1241. https://doi.org/10.18203/2394-6040.ijcmph20220680
73. Takenaka S, Sotozono M, Ohkura N, Noiri Y. Evidence on the Use of Mouthwash for the Control of Supragingival Biofilm and Its Potential Adverse Effects. Antibiotics (Basel). 2022 May 28;11(6):727. doi: 10.3390/antibiotics11060727.
74. Takenaka S, Ohsumi T, Noiri Y. Evidence-based strategy for dental biofilms: Current evidence of mouthwashes on dental biofilm and gingivitis. Jpn Dent Sci Rev. 2019 Nov;55(1):33-40. doi: 10.1016/j.jdsr.2018.07.001.
75. Mythri H, Ananda S, Prashant G, Reddy VS, Chandu G. The efficacy of antiseptic mouth rinses in comparison with dental floss in controlling interproximal gingivitis. J Int Soc Prev Community Dent. 2011;1(1):31-35.
76. Marinho VCC, Higgins J, Logan S, Sheiham A. Fluoride toothpastes for preventing dental caries in children and adolescents. Cochrane Database Syst Rev. 2003;(1): CD002278.
77. Walsh T, Worthington HV, Glenny AM, Marinho VC, Jeroncic A. Fluoride toothpastes of different concentrations for preventing dental caries. Cochrane Database Syst Rev. 2019 Mar 4;3(3): CD007868. doi: 10.1002/14651858.CD007868.pub3.
78. Folayan MO, Adeniyi A, Oziegbe EO, et al. Use of caries prevention tools and associated caries risk in a suburban population of children in Nigeria. Eur Arch Paediatr Dent. 2016;17(3):187-193.
79. Foláyan MO, Zuñiga RAA, Mohebbi SZ, Khami MR. Associations between the use of caries preventive methods and ECC experience in Ile-Ife, Nigeria: a cross-sectional study. BMC Oral Health. 2025 Sep 29;25(1):1477. doi: 10.1186/s12903-025-06721-9.
80. Almas K. The antimicrobial effects of extracts of Azadirachta indica (Neem) and Salvadora persica (Arak) chewing sticks. Indian J Dent Res. 1999 Jan-Mar;10(1):23-6. PMID: 10865390.
81. Saha S, Mohammad S, Saha S, Samadi F. Efficiency of traditional chewing stick (miswak) as an oral hygiene aid among Muslim school children in Lucknow: A cross-sectional study. J Oral Biol Craniofac Res. 2012 Sep-Dec;2(3):176-80. doi: 10.1016/j.jobcr.2012.10.009.
82. Hujoel P. P, Hujoel M. L. A., Kotsakis G. A. (2018). Personal oral hygiene and dental caries: a systematic review of randomised controlled trials. Gerodontology 35, 282–289. 10.1111/ger.12331
83. Roland E, Gueguen G, Longis MJ, Boiselle J. (1994). Validation of the reproducibility of the dmf index used in bucco-dental epidemiology and evaluation of its 2 clinical forms. World Health Stat Q, 47(2): 44–61.

84. Anaise JZ. Measurement of dental caries experience--modification of the DMFT index. Community Dent Oral Epidemiol. 1984 Feb;12(1):43-6. doi: 10.1111/j.1600-0528.1984.tb01408.x.
85. Broadbent JM, Thomson WM. (2005). For debate: Problems with the dmf index pertinent to dental caries data analysis. Community Dent Oral Epidemiol, 33(6): 400–9.
86. Gudipaneni RK, Alkuwaykibi AS, Ganji KK, Bandela V, Karobari MI, Hsiao CY, Kulkarni S, Thambar S. Assessment of caries diagnostic thresholds of DMFT, ICDAS II, and CAST in the estimation of caries prevalence rate in first permanent molars in early permanent dentition-a cross-sectional study. BMC Oral Health. 2022 Apr 20;22(1):133. doi: 10.1186/s12903-022-02134-0.
87. Abhishek M. Comprehensive review of caries assessment systems developed over the last decade. RSBO Revista Sul-Brasileira de Odontologia. 2012; 9(3): 316-321. Available at: https://www.redalyc.org/articulo.oa?id=153023729015
88. Featherstone JDB, Fontana M, Wolff M, et al. A comparison of four caries risk assessment methods. Front Oral Health. 2021;2:656558.

Click here to access/download
Supporting Information - Compressed/ZIP File Archive
Supplemental Files.zip
