# Supplementary material for: Oral hygiene practices and dental caries experience in Nigeria: A systematic review and meta-analysis
Source: PLoS One. 2026 Aug 11;21(8):e0342631. doi: 10.1371/journal.pone.0342631 (PMC13460621; doi:10.1371/journal.pone.0342631)
Supplement: Supplementary file 5 [file pone.0342631.s006.docx]

The manuscript “Oral Hygiene Practices and Dental Caries Experience in Nigeria: A Systematic Review and Meta-Analysis” is a systematic review and meta-analysis examining the relationship between oral hygiene practices and dental caries in Nigeria. It included 23 studies, mostly cross-sectional, and found that tooth brushing at least twice daily was associated with lower caries prevalence, while the type of cleaning device was not significantly related to caries risk. The review also reported a surprising pooled finding that poor oral hygiene was associated with lower odds of caries, which the authors suggest may reflect confounding factors such as socio-economic status or diet. Most included studies came from Southern Nigeria, highlighting limited national representativeness and the need for more research from Northern regions. Overall, the paper addresses an important public health issue and provides a useful synthesis of existing Nigerian evidence on oral hygiene and dental caries. For this reason, I can recommend this manuscript for publication in PLoS One. However, I would like to ask the authors to address some major/minor issues that I expose below.

- The pooled finding that poor oral hygiene is associated with *lower* caries odds is counterintuitive and conflicts with much of the included study-level evidence. The authors should clarify exposure coding, data extraction, and whether any studies were directionally misclassified.
- The reported protective effect of brushing at least twice daily (OR 0.01) appears implausibly large for observational data. The authors should verify the calculation, report contributing studies and event counts, and perform additional robustness checks.
- The review’s national conclusions are limited by strong geographic imbalance, since most included studies were from Southern Nigeria and nearly all were cross-sectional. The claims should be framed more cautiously.
- The reporting of sensitivity analysis and certainty assessment needs improvement. The manuscript should more clearly show all planned robustness checks and justify the omission of formal certainty grading.
- Some reporting details need revision, especially the ethics, funding, and data availability statements, to improve clarity and align with journal requirements.
- I believe that including some additional relevant reference would help strengthen the manuscript and better support the methodology section. For example: 10.59400/jam.v1i3.91
