# Supplementary material for: Oral hygiene practices and dental caries experience in Nigeria: A systematic review and meta-analysis
Source: PLoS One. 2026 Aug 11;21(8):e0342631. doi: 10.1371/journal.pone.0342631 (PMC13460621; doi:10.1371/journal.pone.0342631)
Supplement: Peer review history

# Peer review: Decision Letter 0

**DOI:** 10.1371/journal.pone.0342631.r001

**Authors:** Ayodeji Oginni

15 May 2026

Dear Dr. Aborisade,

However, I recommend that you carefully consider all reviewers’ comments and address them as fully as possible, using your responses to improve the manuscript’s quality. I also want to emphasize that you adhere to the style and format of PLOS One. Your abstract should not exceed 300 words, as it describes the main objective(s) of the study, explains how the study was done, without methodological detail, and summarizes the most important results and their significance

plosone@plos.org. When you’re ready to submit your revision, log on to https://www.editorialmanager.com/pone/ and select the ‘Submissions Needing Revision’ folder to locate your manuscript file.

- A letter that responds to each point raised by the academic editor and reviewer(s). You should upload this letter as a separate file labeled 'Response to Reviewers'.
- A marked-up copy of your manuscript that highlights changes made to the original version. You should upload this as a separate file labeled 'Revised Manuscript with Track Changes'.
- An unmarked version of your revised paper without tracked changes. You should upload this as a separate file labeled 'Manuscript'.

If you would like to make changes to your financial disclosure, please include your updated statement in your cover letter. Guidelines for resubmitting your figure files are available below the reviewer comments at the end of this letter.

If applicable, we recommend that you deposit your laboratory protocols in protocols.io to enhance the reproducibility of your results. Protocols.io assigns your protocol its own identifier (DOI) so that it can be cited independently in the future. For instructions see: https://journals.plos.org/plosone/s/submission-guidelines#loc-laboratory-protocols. Additionally, PLOS ONE offers an option for publishing peer-reviewed Lab Protocol articles, which describe protocols hosted on protocols.io. Read more information on sharing protocols at https://plos.org/protocols?utm_medium=editorial-email&utm_source=authorletters&utm_campaign=protocols.

As the corresponding author, your ORCID iD is verified in the submission system and will appear in the published article. PLOS supports the use of ORCID, and we encourage all coauthors to register for an ORCID iD and use it as well. Please encourage your coauthors to verify their ORCID iD within the submission system before final acceptance, as unverified ORCID iDs will not appear in the published article. *Only*  the individual author can complete the verification step; PLOS staff *cannot*  verify ORCID iDs on behalf of authors.

We look forward to receiving your revised manuscript.

Kind regards,

Ayodeji Babatunde Oginni

Academic Editor

PLOS One

Journal Requirements:

When submitting your revision, we need you to address these additional requirements.

1. Please ensure that your manuscript meets PLOS ONE’s style requirements, including those for file naming. The PLOS ONE style templates can be found at

https://journals.plos.org/plosone/s/file?id=wjVg/PLOSOne_formatting_sample_main_body.pdf and https://journals.plos.org/plosone/s/file?id=ba62/PLOSOne_formatting_sample_title_authors_affiliations.pdf

2. Your ethics statement should only appear in the Methods section of your manuscript. If your ethics statement is written in any section besides the Methods, please delete it from any other section.

3. We note that you have indicated that there are restrictions to data sharing for this study. PLOS only allows data to be available upon request if there are legal or ethical restrictions on sharing data publicly. For more information on unacceptable data access restrictions, please see http://journals.plos.org/plosone/s/data-availability#loc-unacceptable-data-access-restrictions.

Before we proceed with your manuscript, please address the following prompts:

a) If there are ethical or legal restrictions on sharing a de-identified data set, please explain them in detail (e.g., data contain potentially identifying or sensitive patient information, data are owned by a third-party organization, etc.) and who has imposed them (e.g., a Research Ethics Committee or Institutional Review Board, etc.). Please also provide contact information for a data access committee, ethics committee, or other institutional body to which data requests may be sent.

b) If there are no restrictions, please upload the minimal anonymized data set necessary to replicate your study findings to a stable, public repository and provide us with the relevant URLs, DOIs, or accession numbers. For a list of recommended repositories, please see

https://journals.plos.org/plosone/s/recommended-repositories. You also have the option of uploading the data as Supporting Information files, but we would recommend depositing data directly to a data repository if possible.

We will update your Data Availability statement on your behalf to reflect the information you provide.

4. In the online submission form, you indicated that all datasets generated and analyzed, including the study protocol, search strategy, list of included and excluded studies, data extracted, analysis plans, and quality assessment, are available in the article and upon request from the corresponding author.

All PLOS journals now require all data underlying the findings described in their manuscript to be freely available to other researchers, either 1. In a public repository, 2. Within the manuscript itself, or 3. Uploaded as supplementary information.

This policy applies to all data except where public deposition would breach compliance with the protocol approved by your research ethics board. If your data cannot be made publicly available for ethical or legal reasons (e.g., public availability would compromise patient privacy), please explain your reasons on resubmission and your exemption request will be escalated for approval.

5. Please update your submission to use the PLOS LaTeX template. The template and more information on our requirements for LaTeX submissions can be found at http://journals.plos.org/plosone/s/latex.

6. Please amend the manuscript submission data (via Edit Submission) to include author Oliver Chukwujekwu Ezechi.

7. Please ensure that you refer to Figure 2, 3, 6, 8, 9 in your text as, if accepted, production will need this reference to link the reader to the figure.

8. We note you have included a table to which you do not refer in the text of your manuscript. Please ensure that you refer to Table 3, 4 in your text; if accepted, production will need this reference to link the reader to the Table."

9. Please include captions for your Supporting Information files at the end of your manuscript, and update any in-text citations to match accordingly. Please see our Supporting Information guidelines for more information: http://journals.plos.org/plosone/s/supporting-information.

10. If the reviewer comments include a recommendation to cite specific previously published works, please review and evaluate these publications to determine whether they are relevant and should be cited. There is no requirement to cite these works unless the editor has indicated otherwise.

[Note: HTML markup is below. Please do not edit.]

Reviewers' comments:

Reviewer’s Responses to Questions

**Comments to the Author**

1. Is the manuscript technically sound, and do the data support the conclusions?

Reviewer #1: Yes

Reviewer #2: Yes

Reviewer #3: Yes

Reviewer #4: Yes

Reviewer #5: Yes

Reviewer #6: Yes

**********

2. Has the statistical analysis been performed appropriately and rigorously?

Reviewer #1: Yes

Reviewer #2: Yes

Reviewer #3: Yes

Reviewer #4: Yes

Reviewer #5: Yes

Reviewer #6: Yes

**********

3. Have the authors made all data underlying the findings in their manuscript fully available?

The PLOS Data policy

Reviewer #1: Yes

Reviewer #2: Yes

Reviewer #3: Yes

Reviewer #4: No

Reviewer #5: Yes

Reviewer #6: Yes

**********

4. Is the manuscript presented in an intelligible fashion and written in standard English?

Reviewer #1: Yes

Reviewer #2: Yes

Reviewer #3: Yes

Reviewer #4: Yes

Reviewer #5: Yes

Reviewer #6: Yes

**********

Reviewer #1: Financial disclosure statement: revise the statement ‘The funders play any role in the study design, data collection and analysis, decision to publish, or preparation of the manuscript?’

Abstract:

1) bit odd to put 0.00, better <0.01

2) poor oral hygiene was associated with a 38% reduction in caries odds: isn’t this supposed to be good oral hygiene associated with lower caries odds.

3) Unsure if needed to put the most common cleaning tool if reported no significant association between type of cleaning device and caries prevalence

4) Unsure why highlight the technique, as this was not primarily assessed in the study

Introduction

5) Key oral hygiene methods include regular tooth brushing with a toothbrush and toothpaste, which removes dental plaque and food particles, prevents periodontal diseases, and dental caries. ; perhaps could revise to… as a prevention from dental caries – this paper is solely about dental caries, therefore, I don’t see a need to emphasise on periodontal disease as well.

6) Other practices, such as dental floss or interdental cleaning, antimicrobial rinses, and tongue cleaning, complement tooth brushing in controlling plaque accumulation and maintaining oral health – perhaps could revise to …Besides toothbrushing, the use of dental floss, interdental cleaning, antimicrobial rinses, and tongue cleaning, controls plaque accumulation. � saying maintaining oral health is repeating what the second sentence of introduction entails. All of these actions are maintenance of oral health

7) Furthermore, oral hygiene includes thorough cleaning of the dorsum of the tongue using a tongue scraper or a toothbrush, which helps to eliminate bacteria and debris from the oral cavity because the tongue can be a reservoir for oral bacteria – perhaps could revise to…Furthermore, cleaning of the dorsum of the tongue using a tongue scraper or a toothbrush helps to eliminate debris that can be oral bacteria reservoir.

8) The link between oral hygiene and dental caries is well-established, as dental plaque plays a central role in initiating and advancing dental caries lesions [10, 11], which suggests that efforts to control plaque would positively affect the prevention of dental caries [12]. – perhaps could revise to… The link between oral hygiene and dental caries is well-established, as dental plaque initiates and advances dental caries lesions [10, 11], therefore, efforts to control plaque would positively affect the prevention of dental caries [12].

9) Regular toothbrushing, fluoridated toothpaste, - this should be regular toothbrushing with fluoridated toothpaste as these two combinations is the recommended.

10) Overall, please check the full stop placement either needed before the reference’s numbers or not. A lot of inconsistencies as some are put before and after, and others are only after. If I wasn’t mistaken, only after references was needed.

11) … when combined with professional dental care – do you mean here in terms of preventive treatment or more like scaling and polishing, how so professional dental care helps reduce caries

12) Is there a specific reason or challenges related to oral health systems on why the last national survey on Nigeria was conducted for quite some time. could help readers get a sense of understanding that this systematic reviews help to inform policymakers using existing evidence, if limitations exist to conduct periodic national oral health surveys.

13) Reading from abstracts, noted that there were subgroup analyses of moderation of dentition type and relevance of region of studies conducted. Would be nice in the introduction if readers get a sense of how the differences exist and why does it matter to assess the moderation effect based on these two categories. Noted that in the method that geopolitical zone South and Northern was used. Perhaps could add a bit in literature review about regional differences is it due to locality of capital in which region, or is it about the geography of the location causing inaccessibility to healthcare and lower resources. On that note, maybe the aim should also include this as this is mentioned in the abstract as main findings.

Method

14) Noted that search duration in inclusion criteria is from January 2001 to December 2025, this should be placed in explicit in the abstract as for now, looks like it includes everything until January 2026 (gives meaning that studies before 2001 included too).

15) Any specific reasons the libraries of the National Postgraduate Medical College of Nigeria and the West African College of Surgeons were used for grey literature. Is this the biggest library with huge sources or anything related to authors locality?

16) Excluded studies are reported in Supplemental File 2 – should be ‘were’ reported

17) this review, dental caries was the primary outcome of interest – is there any secondary outcome of interest. Assuming not, then maybe avoid using primary here, outcome of interest is sufficient.

Results

18) Noted the word as shown in Figure 1 about the 1422 records but couldn’t find this number in the figure. Understood that this is combination of from databases and other sources but could be confusing to make that conclusion if the number is not exactly shown in the figure or written in the text that it is combination of both. Same goes to the 23 met inclusion criteria, understood this is the combination of both sources, perhaps could include this number in the figure with arrows showing the combined studies from both as this is the exact figure that will be used throughout the results and discussion.

19) Dental caries assessment. All 23 studies…. – perhaps could change the order so it says primary � mixed � permanent dentition, as you write in the method so it is more consistent

20) Association between Oral Hygiene Practices and Dental Caries….(OR: 17.7) – perhaps could add the 95%CI (if exists) from the study. On the same subheading,… supervisor of brushing – perhaps the term could fit better with brushing supervision

21) Table 3: in general, there are no supporting 95%CI included for all the OR and AOR, is there a specific reason why is this is done. If not, perhaps could find back from respective studies and include 95%CI.

22) Subgroup analyses - in this section the sequence is mixed, primary, permanent. Unsure why is that, as usually we would wanna see how it differs from primary, mixed, permanent as to get a general sense of how partly from the age factor, caries and dentition changed. Also on that note, the 95%CI should be included after the 49% reduction, especially because such wide confidence interval from only 2 studies with small sample.

23) Figure 2 and 3 were not written in the texts, in which texts or paragraphs does this figure fit into. On that note, as Figure 3 is specifically about oral hygiene status and caries prevalence, this needs to be specified in the caption. Would be extra easier for each figure regarding this measure: oral hygiene status has the number of studies included in the caption (n=10). Figure 4 now is about OHI, perhaps could standardised: either OHI throughout or oral hygiene status. Also, OHI was not mentioned in the text about the abbreviation, can see OHI-S. is it saying the same thing

24) Subgroup analysis across geopolitical zone was not possible as only Southwest zone had sufficient study for a meta-analysis (OR 0.63, 95% CI: 0.41–0.96) as the Southeastern zone and the Northern region only had two and one studies each respectively. – this sentence is quite confusing, perhaps could say that due to limited study in Southeastern (n=2) and Northern (n=1) region, subgroup analyses across geopolitical zone was not possible to be conducted.

25) Because fewer than 10 included research studies were included, following the removal of the influential study, we could not evaluate the included studies’ publication bias, as the lack of statistical power from the inclusion of a few studies would cause [42]. – this sentence is a bit confusing. Firsly, where does these 10 included research number comes from. Isn’t 23 the included studies combining the two large resources, this confusing section happens because of have many measures and many subheadings. It’s a bit hard to follow and trace back if all are writing continuously without subheading numbering. Secondly, ‘the removal of influential study’, does it mean the total is 9 included studies now. Also, even if without removal of that one study, would 10 studies be enough to do publication bias assessment. Do you mean in general: … A lack of statistical power from the small number of included studies limits the ability to assess publication bias for oral hygiene status and caries prevalence.

26) Figure 6 - in general there is no such number of 0.00 odds ratio, understand this is a round off to two decimal points, but what you can say is <0.01. saying zero implied it is exactly zero, while based on your figure, it is actually ranging from <0.01 to <0.001

27) Figure 7 caption, again could be specify that this figure is related to the toothbrushing and dental caries

28) Association between frequency of tooth brushing and dental caries, in the subgroup analysis, the sentence missed full stop at the end of the sentence.

29) Association between tooth cleaning methods and dental caries - … (e.g toothbrush) – don’t think e.g is needed here as you are including every method under this first section of analyses.

30) Subgroup analyses for tooth cleaning methods. First word of the paragraph is ‘Toothbrushing’ – unsure in this subgroup analyses only studies where cleaning methods toothbrushing were assessed here. I thought this section was about all tooth cleaning methods. Need to be consistent in terms of terminology used, either tooth cleaning methods all the way, and only for toothbrush if the analysis is about toothbrushing only. Another point, here is where 95%Ci needed in the text as saying 92% increase is quite a high number, but with such wide CI, cautious writing must be done in making such claim. Also, using the word ‘increase’ here might not be suitable as the word increase implied a comparison of before or after, where may be more suitable for cohort analyses. As your results were all observational, perhaps ‘higher prevalence’ fits better.

Discussion

31) Would suggest to reorder the strength and limitation right before conclusion as the main discussion could be centred around the findings first

32) Suggestion: the whole order of discussion could be more easier to understand if focus on the findings results, then what others additional elements that could provide more to the narrative. I don’t quite understand why you start your discussion with dietary intake as this was not at all observed in your study, while writing about the second insight about twice daily toothbrushing. The second insight should be the main point as this is what is shown from your study. The …contradicts simplistic biological models is supplementary to paint the bigger picture so it fits in the later part.

33) The significant association between poor oral hygiene, dental caries, and the primary dentition also needs to be explored further, as it is currently under-explored in its full complexity because of the lifelong consequences. – sentence quite confusing, as what do you mean by full complexity & lifelong consequences. Do you think more cohort studies can address this, or is it need to be more community approach or how

34) In this paragraph…The efficacy of twice-daily tooth brushing, the last part was confusing. You start by saying using dental floss can augment effectiveness, which is correct, and also true that from your included study, this finding was not substantial. But then you suggest mouthwash out of nowhere, which the narrative comes out of the sudden and implying that mouthwash could gives a different result. Understood the logic, but argument is a bit weak in terms of bridging this gap with future studies could bring more light just by adding mouthwash.

35) A culturally significant finding of this meta-analysis is that toothbrushing with toothpaste was not superior to other devices in reducing the risk for caries – correct me if I am wrong, but my impression based on your study is that you were looking at the meta analyses of all the cleaning methods and the prevalence of dental caries. Am I missing subgroup analyses where you compare the odds of using toothbrush w toothpaste vs other devices,. The points written after that makes sense, but the first part was quite confusing. Do you mean in general, it doesn’t matter what kind of cleaning methods you use, the caries risk will be reduced anyway. Perhaps rewording to the emphasis on cleaning itself. On the same paragraph: on technique and consistency. Agreed on consistency cause you assess twice daily toothbrushing having reduced caries, but unsure how technique fits here as you don’t assess this.

36) This sentence ‘A well-used chewing stick may clean more effectively than a poorly used toothbrush. This finding raises an important hypothesis that needs to be tested in future studies,’. A bit confusing here, one is that are you referring to your study finding that well used chewing stick may clean more effectively (could not find the evidence in your result or one of your aim in assessing either different cleaning methods give different reduction of caries risk). Two, is this the hypothesis that should be tested, which in that case, fair enough, it is an interesting research question to be addressed

37) For this part - For clinicians, this finding may necessitate a shift in counselling. Rather than dismissing traditional practices, oral health professionals should engage patients with respect, asking what they use, how often, and demonstrate effective techniques. – what is the traditional practices here, unsure what do you mean by that, are you implying the traditional practice did the opposite of engaging patient with respect…etc.. also what is the shift from, what is not currently practice.

38) At the community and public health level, the study evidence supports the need for campaigns that empower rather than prescribe. – this part is contradicting than the first part where you suggest counselling message is about doing this and that. Why is that at community and public health should empower rather than prescribe. Is there any supporting policy that could provide resources to the respective department in community engagement. What do you mean here not prescribing that is ‘bad’

Reviewer #2: The systematic review was scientifically sound and in compliance with the standard guidelines for writing such reviews. The title of the manuscript appears pertinent to the Nigerian context, where both individuals and the government neglect oral health status. The review findings were systematically presented, appropriately linked to the aim of the manuscript, and logically concluded for a global reference. The discussion section was well articulated, relating to existing research findings and proposed future interventions. Furthermore, the study limitations were well highlighted, while suggestions for future studies were noted.

Reviewer #3: The comments to the author are in the text. There is need to structure the paper according to the PRIMSA 2020 checklist. Also, the research question in a systematic review is classically defined using a framework, thereafter, study objectives are given.

Reviewer #4: The manuscript “Oral Hygiene Practices and Dental Caries Experience in Nigeria: A Systematic Review and Meta-Analysis” is a systematic review and meta-analysis examining the relationship between oral hygiene practices and dental caries in Nigeria. It included 23 studies, mostly cross-sectional, and found that tooth brushing at least twice daily was associated with lower caries prevalence, while the type of cleaning device was not significantly related to caries risk. The review also reported a surprising pooled finding that poor oral hygiene was associated with lower odds of caries, which the authors suggest may reflect confounding factors such as socio-economic status or diet. Most included studies came from Southern Nigeria, highlighting limited national representativeness and the need for more research from Northern regions. Overall, the paper addresses an important public health issue and provides a useful synthesis of existing Nigerian evidence on oral hygiene and dental caries. For this reason, I can recommend this manuscript for publication in PLoS One. However, I would like to ask the authors to address some major/minor issues that I expose below.

�The pooled finding that poor oral hygiene is associated with lower caries odds is counterintuitive and conflicts with much of the included study-level evidence. The authors should clarify exposure coding, data extraction, and whether any studies were directionally misclassified.

�The reported protective effect of brushing at least twice daily (OR 0.01) appears implausibly large for observational data. The authors should verify the calculation, report contributing studies and event counts, and perform additional robustness checks.

�The review’s national conclusions are limited by strong geographic imbalance, since most included studies were from Southern Nigeria and nearly all were cross-sectional. The claims should be framed more cautiously.

�The reporting of sensitivity analysis and certainty assessment needs improvement. The manuscript should more clearly show all planned robustness checks and justify the omission of formal certainty grading.

�Some reporting details need revision, especially the ethics, funding, and data availability statements, to improve clarity and align with journal requirements.

�I believe that including some additional relevant reference would help strengthen the manuscript and better support the methodology section. For example: 10.59400/jam.v1i3.91

Reviewer #5: This manuscript presents a timely and well-executed systematic review and meta-analysis examining the association between oral hygiene practices and dental caries prevalence in Nigeria. The study is clearly written, methodologically sound, and addresses an important public health issue, particularly in low- and middle-income settings.

The authors demonstrate rigor through comprehensive database searches, PROSPERO registration, and clearly defined inclusion/exclusion criteria. The use of random-effects models, subgroup analyses, and assessment of heterogeneity and publication bias strengthens the credibility of the findings. The interpretation of results is generally balanced and acknowledges unexpected findings, such as the inverse association between poor oral hygiene and caries, which is appropriately discussed in the context of potential confounding factors.

The results are informative and contribute meaningful insights, particularly the strong protective association of twice-daily tooth brushing and the limited role of cleaning tools compared to brushing behavior. The identification of geographic gaps in the literature (notably the underrepresentation of Northern Nigeria) is an important contribution and provides direction for future research.

Minor suggestions for improvement:

Clarify the unexpected finding regarding poor oral hygiene and reduced caries odds, possibly expanding discussion on residual confounding or measurement variability.

Provide a brief comment on the extremely small OR (0.01) for brushing frequency, as this may raise questions about effect size plausibility or data scaling.

Consider adding a short note on policy or public health implications for Nigeria.

Overall, this is a high-quality manuscript that makes a valuable contribution to the literature. I recommend acceptance after minor revisions.

Reviewer #6: The authors have explored “Oral Hygiene Practices and Dental Caries Experience in Nigeria: A Systematic Review and Meta-Analysis ”. The work is publishable in this journal after addressing the following minor comments.

1. Explain Thorium Radioactivity Concentrations?

2. Why is this study carried out?

3. How this study becomes novel?

4. How this study can be helpful to the prosperity of the country?

5. Highlight the main finding the in the abstract.

6. What are the ranges of the parameters involved in the study?

7. Write down the main finding in the conclusion.

8. Check whole manuscript regarding spell and punctions.

**********

what does this mean?). If published, this will include your full peer review and any attached files.

If you choose “no”, your identity will remain anonymous but your review may still be made public.

**Do you want your identity to be public for this peer review?**  For information about this choice, including consent withdrawal, please see our Privacy Policy

Reviewer #1: **Yes:** Abdul Azim Abdul Razak

Reviewer #2: No

Reviewer #3: No

Reviewer #4: No

Reviewer #5: **Yes:** Dr Nasir Abbas

Reviewer #6: No

**********

[NOTE: If reviewer comments were submitted as an attachment file, they will be attached to this email and accessible via the submission site. Please log into your account, locate the manuscript record, and check for the action link "View Attachments". If this link does not appear, there are no attachment files.]

To ensure your figures meet our technical requirements, please review our figure guidelines: https://journals.plos.org/plosone/s/figures

You may also use PLOS’s free figure tool, NAAS, to help you prepare publication quality figures: https://journals.plos.org/plosone/s/figures#loc-tools-for-figure-preparation.

NAAS will assess whether your figures meet our technical requirements by comparing each figure against our figure specifications.

Attachment Submitted filename: PONE-D-26-04211_reviewer.pdf

Attachment Submitted filename: Revision PONE-D-26-04211.docx

# Author comment: Author response to Decision Letter 1

**DOI:** 10.1371/journal.pone.0342631.r002

6 Jul 2026

Journal: PLOS One

Manuscript ID: PONE-D-26-04211

Manuscript title: Oral Hygiene Practices and Dental Caries Experience in Nigeria: A Systematic Review and Meta-Analysis

Date of revision: 28th May 2026

We thank the reviewers for the constructive feedback. This has helped strengthen the manuscript. Below is a point-by-point response to the reviewers’ comments.

Reviewer #1: Financial disclosure statement: revise the statement ‘The funders play any role in the study design, data collection and analysis, decision to publish, or preparation of the manuscript?’

Response: We have revised the financial disclosure statement. The funders played no role in the study design, data collection and analysis, decision to publish, or preparation of the manuscript

Abstract:

1) bit odd to put 0.00, better <0.01

Response: We have adjusted it to <0.01 as rightly suggested. We wrote: Brushing at least twice daily was strongly associated with reduced caries (OR 0.01, 95% CI: <0.00–0.01).

2) Poor oral hygiene was associated with a 38% reduction in caries odds: isn’t this supposed to be good oral hygiene associated with lower caries odds?

Response: Thanks for the comments. An extensive review of the literature was on and we identified an error in the coding of one of the included studies. The re-computation now shows that poor OH was associated with 76% increase in caries odds. Poor oral hygiene was associated with a 76% increase in the odds for dental caries (OR 1.76, 95% CI: 1.41–2.83, p<0.001).

3) Unsure if needed to put the most common cleaning tool, as reported no significant association between the type of cleaning device and caries prevalence

Response: Thanks for picking this. We have deleted the reference to significant association but retained the details on the oral cleaning tools, as this was part of the study objectives.

4) Unsure why highlight the technique, as this was not primarily assessed in the study

Response: Thanks for highlighting this. The statement has been removed.

Introduction

5) Key oral hygiene methods include regular tooth brushing with a toothbrush and toothpaste, which removes dental plaque and food particles, prevents periodontal diseases, and dental caries. Perhaps could revise to… as a prevention from dental caries – this paper is solely about dental caries, therefore, I don’t see a need to emphasise periodontal disease as well.

Response: We agree with the comment and have removed the reference to periodontal diseases

6) Other practices, such as dental floss or interdental cleaning, antimicrobial rinses, and tongue cleaning, complement tooth brushing in controlling plaque accumulation and maintaining oral health – perhaps could revise to …Besides toothbrushing, the use of dental floss, interdental cleaning, antimicrobial rinses, and tongue cleaning controls plaque accumulation. � Saying that maintaining oral health is repeating what the second sentence of the introduction entails. All of these actions are maintenance of oral health

Response: We have revised the statement as recommended

7) Furthermore, oral hygiene includes thorough cleaning of the dorsum of the tongue using a tongue scraper or a toothbrush, which helps to eliminate bacteria and debris from the oral cavity because the tongue can be a reservoir for oral bacteria – perhaps could revise to…Furthermore, cleaning of the dorsum of the tongue using a tongue scraper or a toothbrush helps to eliminate debris that can be an oral bacterial reservoir.

Response: Thanks for the suggestion. We have revised as suggested.

8) The link between oral hygiene and dental caries is well-established, as dental plaque plays a central role in initiating and advancing dental caries lesions [10, 11], which suggests that efforts to control plaque would positively affect the prevention of dental caries [12]. – perhaps could revise to… The link between oral hygiene and dental caries is well-established, as dental plaque initiates and advances dental caries lesions [10, 11]; therefore, efforts to control plaque would positively affect the prevention of dental caries [12].

Response: Revised as recommended.

9) Regular toothbrushing, fluoridated toothpaste - this should be regular toothbrushing with fluoridated toothpaste, as these two combinations are recommended.

Response: Revised as recommended.

10) Overall, please check the full stop placement, either needed before the reference numbers or not. A lot of inconsistencies, as some are put before and after, and others are only after. If I wasn’t mistaken, only after references were needed.

Response: Thanks for picking this. We have corrected the placement of full stops throughout the manuscript; the in-text citations have been placed before the full stops.

11) … when combined with professional dental care – do you mean here in terms of preventive treatment or more like scaling and polishing, how does professional dental care help reduce caries

Response: This has now been revised to give an example of professional dental care, such as scaling and polishing.

12) Is there a specific reason or challenges related to oral health systems that the last national survey in Nigeria was conducted quite some time ago? could help readers get a sense of understanding that these systematic reviews help to inform policymakers using existing evidence, if limitations exist in conducting periodic national oral health surveys.

Response: Thanks for highlighting this. Actually, no national surveys by the government have been conducted. The surveys are individual researcher initiatives. We had noted that there is no national surveillance system for oral health. A longer explanation will be challenging as we have no reference.

13) Reading from abstracts, noted that there were subgroup analyses of the moderation of dentition type and the relevance of the region of studies conducted. Would be nice in the introduction if readers get a sense of how the differences exist and why they matter to assess the moderation effect based on these two categories. Noted that in the method that geopolitical zones South and North were used. Perhaps could add a bit in the literature review about regional differences, whether it is due to the locality of the capital in which region, or is it about the geography of the location, causing inaccessibility to healthcare and lower resources. On that note, maybe the aim should also include this, as this is mentioned in the abstract as a main finding.

Response: Thanks for the suggestion. We have included a statement on regional differences. We wrote: The evidence also needs to support planning for northern and southern Nigeria, where differences in health expenses and disease burden, oral hygiene behaviors, service availability, and sociodemographic conditions demand region specific strategies. We have also included the conduct of a geopolitical zone analysis in the study objective.

14) Oral hygiene is linked with dental caries experience. This systematic review and meta-analysis assessed the associations between oral hygiene status, the frequency of tooth brushing, and the prevalence of dental caries in Nigeria.

Response: We agree with this comment, and we have revised the Background to reflect the suggested edit.

Methods

14) Noted that search duration in inclusion criteria is from January 2001 to December 2025; this should be made explicit in the abstract, as for now, it looks like it includes everything until January 2026 (gives the impression that studies before 2001 are included too).

Response: We have included the duration of the study in the abstract

15) Any specific reasons the libraries of the National Postgraduate Medical College of Nigeria and the West African College of Surgeons were used for grey literature? Is this the biggest library with huge sources or anything related to the authors' locality?

Response: The libraries are the largest local source of postgraduate dissertations for oral health. PhD programs for oral health in Nigeria only emerged in the last 5 years. For this reason, it was a veritable source of grey literature in Nigeria. We wrote: The libraries of the National Postgraduate Medical College of Nigeria and the West African College of Surgeons provide the biggest local library of theses and postgraduate dissertations.

16) Excluded studies are reported in Supplemental File 2 – should be ‘were’ reported

Response: Thanks for the guidance. The statement has been revised.

17) In this review, dental caries was the primary outcome of interest. Is there any secondary outcome of interest? Assuming not, then maybe avoid using primary here, the outcome of interest is sufficient.

Response: Thanks for the guidance. The statement has been corrected as suggested

Results

18) Noted the word as shown in Figure 1 about the 1422 records, but couldn’t find this number in the figure. Understood that this is a combination of databases and other sources, but it could be confusing to make that conclusion if the number is not exactly shown in the figure or written in the text that it is a combination of both. The same goes for the 23 met inclusion criteria. Understanding this is the combination of both sources, perhaps could include this number in the figure with arrows showing the combined studies from both, as this is the exact figure that will be used throughout the results and discussion.

Response: The figures have now been reflected in the PRISMA flow diagram

19) Dental caries assessment. All 23 studies…. – perhaps could change the order so it says primary � mixed � permanent dentition, as you write in the method, so it is more consistent

Response: Thanks for the guidance. The order has been revised as suggested.

20) Association between Oral Hygiene Practices and Dental Caries….(OR: 17.7) – Perhaps could add the 95%CI (if exists) from the study. On the same subheading,… supervisor of brushing – perhaps the term could fit better with brushing supervision

Response: Thanks for the guidance. We have now reported on the confidence intervals for the data, and the edit has been effected as suggested.

21) Table 3: In general, there are no supporting 95%CI included for all the OR and AOR. Is there a specific reason why this is done? If not, perhaps we could go back to our respective studies and include 95% CI.

Response: Table 3 has been correctly revised to report the 95% confidence intervals reported for the studies

22) Subgroup analyses - in this section, the sequence is mixed, primary, permanent. Unsure why that is, as usually we would wanna see how it differs from primary, mixed, and permanent as to get a general sense of how partly from the age factor, caries, and dentition changed. Also on that note, the 95%CI should be included after the 49% reduction, especially because such a wide confidence interval is from only 2 studies with a small sample.

Response: The 95 CI has been included, and the order of reporting the findings for each type of dentition has been revised.

23) Figures 2 and 3 were not written in the texts; in which texts or paragraphs does this figure fit? On that note, as Figure 3 is specifically about oral hygiene status and caries prevalence, this needs to be specified in the caption. Would be extra easier for each figure regarding this measure: oral hygiene status has the number of studies included in the caption (n=10). Figure 4 now is about OHI, which could be standardised: either OHI throughout or oral hygiene status. Also, OHI was not mentioned in the text about the abbreviation, but OHI-S can be seen. Is it saying the same thing

Response: Thanks for highlighting this error. Figure 2 has been referred to in the text, and Figure 3 has now been reported. We have used oral hygiene status throughout the manuscript. Figure 4 has now been labelled properly.

24) Subgroup analysis across geopolitical zones was not possible as only the Southwest zone had sufficient studies for a meta-analysis (OR 0.63, 95% CI: 0.41–0.96), as the Southeastern zone and the Northern region only had two and one study each, respectively. – This sentence is quite confusing; perhaps you could say that due to limited study in Southeastern (n=2) and Northern (n=1) regions, subgroup analyses across geopolitical zones could not be conducted.

Response: We agree with the suggested revision for clarity, and we have revised the statement as suggested.

25) Because fewer than 10 included research studies were included, following the removal of the influential study, we could not evaluate the included studies’ publication bias, as the lack of statistical power from the inclusion of a few studies would cause [42]. – This sentence is a bit confusing. Firstly, where do these 10 included research numbers come from? Isn’t 23 the number of included studies combining the two large resources? This confusing section happens because of have many measures and many subheadings. It’s a bit hard to follow and trace back if all are writing continuously without subheading numbering. Secondly, ‘the removal of influential study’, does it mean the total is 9 included studies now? Also, even without the removal of that one study, would 10 studies be enough to do a publication bias assessment? Do you mean in general: … A lack of statistical power from the small number of included studies limits the ability to assess publication bias for oral hygiene status and caries prevalence.

Response: You appropriately represented the message we tried to convey. We have edited the section using the suggestions you raised. We are grateful for the support.

26) Figure 6 - In general, there is no such number of 0.00 odds ratios. Understand this is a round off to two decimal points, but what you can say is <0.01. saying zero implied it is exactly zero, while based on your figure, it is actually ranging from <0.01 to <0.001

Response: We have revised to <0.01 as recommended

27) Figure 7 caption, again, could specify that this figure is related to toothbrushing and dental caries

Response: We have revised the caption to show its relation to toothbrushing and dental caries

28) Association between frequency of tooth brushing and dental caries, in the subgroup analysis, the sentence is missing a full stop at the end of the sentence.

Response: Thank you for picking this. We have included the full stop.

29) Association between tooth cleaning methods and dental caries - … (e.g., toothbrush) – don’t think e.g is needed here as you are including every method under this first section of analyses.

Response: … (e.g., toothbrush) has been removed

30) Subgroup analyses for tooth cleaning methods. First word of the paragraph is ‘Toothbrushing’ – unsure in this subgroup analysis, only studies where cleaning methods, such as toothbrushing, were assessed here. I thought this section was about all tooth-cleaning methods. Need to be consistent in terms of terminology used, either for all tooth cleaning methods, or only for the toothbrush, if the analysis is about toothbrushing only. Another point, here is where 95%Ci needed in the text, as saying 92% increase is quite a high number, but with such a wide CI, cautious writing must be done in making such a claim. Also, using the word ‘increase’ here might not be suitable as the word increase ' implies a comparison of before or after, which may be more suitable for cohort analyses. As your results were all observational, perhaps ‘higher prevalence’ fits better.

Response: The association assessed here was for all cleaning methods and the prevalence of dental caries, and we tested the difference between toothbrushing and other cleaning devices in caries prevalence. As suggested, we have used higher prevalence instead of increase. We have also revised the report of the odds ratio as suggested.

Discussion

31) Would suggest reordering the strengths and limitations right before the conclusion, as the main discussion could be centred around the findings first

Response: Thanks for the suggestion. We have retained the reporting on the strengths and limitations in its current position in line with the order of the research reporting guidelines. Study limitations are now reported early to enable readers to identify possible study bias early and read the discussion with th

# Editor report: Decision Letter 1

**DOI:** 10.1371/journal.pone.0342631.r003

**Authors:** Ayodeji Oginni

19 Jul 2026

Dear Dr. Aborisade,

Thank you for submitting your manuscript to PLOS ONE. After careful consideration, we feel that it has merit but does not fully meet PLOS ONE’s publication criteria as it currently stands. Therefore, we invite you to submit a revised version of the manuscript that addresses the points raised during the review process.

Please submit your revised manuscript by Sep 02 2026 11:59PM. If you will need more time than this to complete your revisions, please reply to this message or contact the journal office at plosone@plos.org. When you're ready to submit your revision, log on to https://www.editorialmanager.com/pone/ and select the 'Submissions Needing Revision' folder to locate your manuscript file.

- A letter that responds to each point raised by the academic editor and reviewer(s). You should upload this letter as a separate file labeled 'Response to Reviewers'.
- A marked-up copy of your manuscript that highlights changes made to the original version. You should upload this as a separate file labeled 'Revised Manuscript with Track Changes'.
- An unmarked version of your revised paper without tracked changes. You should upload this as a separate file labeled 'Manuscript'.

If you would like to make changes to your financial disclosure, please include your updated statement in your cover letter. Guidelines for resubmitting your figure files are available below the reviewer comments at the end of this letter.

If applicable, we recommend that you deposit your laboratory protocols in protocols.io to enhance the reproducibility of your results. Protocols.io assigns your protocol its own identifier (DOI) so that it can be cited independently in the future. For instructions see: https://journals.plos.org/plosone/s/submission-guidelines#loc-laboratory-protocols. Additionally, PLOS ONE offers an option for publishing peer-reviewed Lab Protocol articles, which describe protocols hosted on protocols.io. Read more information on sharing protocols at https://plos.org/protocols?utm_medium=editorial-email&utm_source=authorletters&utm_campaign=protocols.

As the corresponding author, your ORCID iD is verified in the submission system and will appear in the published article. PLOS supports the use of ORCID, and we encourage all coauthors to register for an ORCID iD and use it as well. Please encourage your coauthors to verify their ORCID iD within the submission system before final acceptance, as unverified ORCID iDs will not appear in the published article. *Only*  the individual author can complete the verification step; PLOS staff *cannot*  verify ORCID iDs on behalf of authors.

We look forward to receiving your revised manuscript.

Kind regards,

Ayodeji Babatunde Oginni

Academic Editor

PLOS One

Journal Requirements:

If the reviewer comments include a recommendation to cite specific previously published works, please review and evaluate these publications to determine whether they are relevant and should be cited. There is no requirement to cite these works unless the editor has indicated otherwise.

Please review your reference list to ensure that it is complete and correct. If you have cited papers that have been retracted, please include the rationale for doing so in the manuscript text, or remove these references and replace them with relevant current references. Any changes to the reference list should be mentioned in the rebuttal letter that accompanies your revised manuscript. If you need to cite a retracted article, indicate the article’s retracted status in the References list and also include a citation and full reference for the retraction notice.

[Note: HTML markup is below. Please do not edit.]

[NOTE: If reviewer comments were submitted as an attachment file, they will be attached to this email and accessible via the submission site. Please log into your account, locate the manuscript record, and check for the action link "View Attachments". If this link does not appear, there are no attachment files.]

To ensure your figures meet our technical requirements, please review our figure guidelines: https://journals.plos.org/plosone/s/figures

You may also use PLOS’s free figure tool, NAAS, to help you prepare publication quality figures: https://journals.plos.org/plosone/s/figures#loc-tools-for-figure-preparation.

NAAS will assess whether your figures meet our technical requirements by comparing each figure against our figure specifications.

# Author comment: Author response to Decision Letter 2

**DOI:** 10.1371/journal.pone.0342631.r004

20 Jul 2026

Journal: PLOS One

Manuscript ID: PONE-D-26-04211R1

Manuscript title: Oral Hygiene Practices and Dental Caries Experience in Nigeria: A Systematic Review and Meta-Analysis

Date of revision: 20th July 2026

We thank the editor for picking the incomplete sentence, we have rectified the statement.

Besides toothbrushing, the use of dental floss, interdental cleaning, antimicrobial rinses, and tongue cleaning controls plaque accumulation.

# Editor report: Decision Letter 2

**DOI:** 10.1371/journal.pone.0342631.r005

**Authors:** Ayodeji Oginni

23 Jul 2026

Oral Hygiene Practices and Dental Caries Experience in Nigeria: A Systematic Review and Meta-Analysis

PONE-D-26-04211R2

Dear Dr. Aborisade,

We’re pleased to inform you that your manuscript has been judged scientifically suitable for publication and will be formally accepted for publication once it meets all outstanding technical requirements.

Within one week, you’ll receive an e-mail detailing the required amendments. When these have been addressed, you’ll receive a formal acceptance letter and your manuscript will be scheduled for publication.

An invoice will be generated when your article is formally accepted. Please note, if your institution has a publishing partnership with PLOS and your article meets the relevant criteria, all or part of your publication costs will be covered. Please make sure your user information is up-to-date by logging into Editorial Manager at Editorial Manager® and clicking the ‘Update My Information' link at the top of the page. For questions related to billing, please contact billing support.

If your institution or institutions have a press office, please notify them about your upcoming paper to help maximize its impact. If they’ll be preparing press materials, please inform our press team as soon as possible -- no later than 48 hours after receiving the formal acceptance. Your manuscript will remain under strict press embargo until 2 pm Eastern Time on the date of publication. For more information, please contact onepress@plos.org.

Kind regards,

Ayodeji Babatunde Oginni

Academic Editor

PLOS One

Additional Editor Comments (optional):

Reviewers' comments:

# Editor report: Acceptance letter

**DOI:** 10.1371/journal.pone.0342631.r006

**Authors:** Ayodeji Oginni

PONE-D-26-04211R2

PLOS One

Dear Dr. Aborisade,

I'm pleased to inform you that your manuscript has been deemed suitable for publication in PLOS One. Congratulations! Your manuscript is now being handed over to our production team.

At this stage, our production department will prepare your paper for publication. This includes ensuring the following:

* All references, tables, and figures are properly cited

* All relevant supporting information is included in the manuscript submission,

* There are no issues that prevent the paper from being properly typeset

You will receive further instructions from the production team, including instructions on how to review your proof when it is ready. Please keep in mind that we are working through a large volume of accepted articles, so please give us a few days to review your paper and let you know the next and final steps.

Lastly, if your institution or institutions have a press office, please let them know about your upcoming paper now to help maximize its impact. If they'll be preparing press materials, please inform our press team within the next 48 hours. Your manuscript will remain under strict press embargo until 2 pm Eastern Time on the date of publication. For more information, please contact onepress@plos.org.

You will receive an invoice from PLOS for your publication fee after your manuscript has reached the completed accept phase. If you receive an email requesting payment before acceptance or for any other service, this may be a phishing scheme. Learn how to identify phishing emails and protect your accounts at https://explore.plos.org/phishing.

If we can help with anything else, please email us at customercare@plos.org.

Thank you for submitting your work to PLOS ONE and supporting open access.

Kind regards,

PLOS ONE Editorial Office Staff

on behalf of

Ayodeji Babatunde Oginni

Academic Editor

PLOS One
